# Enrichment of Candidate Formate and Acetaldehyde-Producing Ruminal Bacteria in Response to Corn-Based Diets

**DOI:** 10.3390/microorganisms14102134

**Published:** 2026-09-22

**Authors:** Reid F. Anema, Forest L. Francis, Zachary K. Smith, Benoit St-Pierre

**Affiliations:** Department of Animal Science, South Dakota State University, Animal Science Complex, Box 2170, Brookings, SD 57007, USA; reid.anema@sdstate.edu (R.F.A.); francis.forest@yahoo.com (F.L.F.); zachary.smith@sdstate.edu (Z.K.S.)

**Keywords:** sheep, rumen, microbiome, microbiota, 16S rRNA gene, metagenomics, starch, formate, acetaldehyde, ethanol

## Abstract

In ruminant production systems, feeding diets with easily digestible and energy-dense compounds during the finishing phase is a common strategy to ensure optimal growth and performance. As ruminal microbial communities are responsible for fermenting feed into nutrients that the animal host can absorb and use, concentrate-based diets favor specialists that can utilize starch, as well as other cross-feeding species that benefit from their metabolic activities and their end products. Currently, a gradual increase in the proportion of concentrate in rations remains the best strategy towards developing resilience to undesirable end products, thus mitigating their adverse effects on the host. In an effort to gain further insights into the mechanisms involved in the adaptation of ruminal microbial communities to starch-rich diets, we used a 16S rRNA gene-based approach to identify eight highly represented species-level Operational Taxonomic Units (OTUs) that were more abundant in the rumen of finishing lambs fed a whole corn-based diet for 56 (D56, *n* = 7) or 63 days (D63, *n* = 8) compared to samples collected from lambs on a forage diet (*p* < 0.05) that were harvested prior to the dietary transition (D0, *n* = 4). To assess their metabolic capabilities, a metagenomics-based approach was used for OTUs corresponding to uncharacterized bacterial species, while publicly available genomes were analyzed as a proxy for OTUs closely related to known bacterial species. As anticipated, the potential to hydrolyze starch and metabolize glucose into pyruvate was found in all datasets analyzed, but more diverse capabilities were observed for other functions, such as the types of end products synthesized. For instance, lactate was not the only undesirable microbial end product predicted, as the potential to generate formate, acetaldehyde and ethanol was also found across datasets. While the impact of these compounds on the rumen remains poorly explored, ongoing research on how they can affect gut health in humans indicates that they may have undesirable effects. Together, these and other results from this study provide further insight into how ruminal bacterial communities in finishing lambs adapt to starch-based ingredients and how they can in turn affect the ruminal environment.

## 1. Introduction

As they continue to be an essential source of animal protein-based products for human use and consumption, sheep have remained an important livestock species ever since their domestication several millennia ago [1]. The versatility of these ruminants, which can produce edible goods such as meat and milk, as well as fiber, is well reflected in the more than 200 breeds that are recognized worldwide [2]; these are the cumulative result of a wide range of selection strategies spanning several generations that targeted various traits, such as improved performance for single or dual production, as well as optimized management and production practices. Sheep production is not only vital to the economy of global leaders in the industry, such as China, India and Australia [3], it is also an important contributor to the agriculture sector in many other countries. For instance, the American sheep industry has an estimated $1.4 billion in economic impacts in the United States [4], providing animal protein-based products for both domestic and export markets [5].

In ruminant production systems, the main strategies consist of feeding either forage or concentrate as primary dietary components. In the case of the former, animals can graze on pasture or be fed high levels of hay in enclosed areas. While higher plant fiber intake benefits rumen health and function, it can result in slower growth rates, potentially impacting market weight or requiring additional days on feed to meet desired outputs [6]. The concentrate-based strategy aims to promote faster growth during the finishing phase by feeding diets with higher available energy content, which can be supplied through ingredients that are more easily digestible, such as corn or other types of grain [7,8,9]. As these commodities tend to be readily available in many regions of the United States, feeding concentrate-based diets during the finishing phase is a common practice among ruminant livestock producers in this country. Another incentive to feed corn or grain-based diets is consumer preference; indeed, high forage intake can increase linoleic acid content in sheep muscle, a fatty acid prone to oxidation that can increase the risks of off-flavors [10,11,12].

The ability of ruminants to transform inedible plant biomass into protein-based products for human consumption and use is primarily due to the combined metabolic activities of symbiotic microorganisms that reside in the rumen compartment of their four-chambered stomach [13]. These include a broad array of bacterial, archaeal, protozoal, and fungal species that become organized into microbial communities by forming intricate cross-feeding networks [14]. Through mutually beneficial trophic relationships, ruminal microorganisms can collectively metabolize plant tissue and other feed compounds into nutrients that can be utilized by the host animal [15]. Among microbial products generated from ruminal fermentation of feed are short-chain fatty acids (SCFAs) [16], primarily consisting of acetate, propionate and butyrate, which can provide as much as 70% of the energy requirement of their ruminant host [17]. Other contributions come in the form of proteins synthesized during microbial growth in the rumen; as a result of host digestion and absorption during their downstream transit in the gastrointestinal tract, microbial proteins act as a major source of amino acids for ruminants [18].

As grazers, ruminants such as sheep have evolved to feed on plant biomass, and to obtain nutrients by digesting plant cell wall polymers [19]. Since each ruminal microbial species is a metabolic specialist, diet formulation has a profound impact on species composition [20]. Feeding high-concentrate diets effectively applies selective pressure that promotes proliferation of starch utilizers, a major component of corn and grains, while it concurrently lowers the abundance of microorganisms that can only ferment cellulose or other structural polysaccharides. Microbial breakdown of starch and other easily fermentable compounds facilitates higher production of SCFAs, which favors optimal animal performance; however, this also results in increased concentrations of other end products such as lactate, which in combination with SCFAs can lead to a reduction in ruminal pH [21]. A continued state of low pH in the rumen may then progress into subacute ruminal acidosis (SARA) or acidosis, depending on the severity of the condition. Sustained reduced ruminal pH can affect the chemical environment, the physiology of host epithelial cells, as well as the composition and metabolic activities of symbiotic communities [22,23].

The impact of high-grain diets on ruminal bacterial composition has been well documented in cattle [24,25,26,27,28,29,30,31,32,33,34,35,36,37]. Ruminal microbial communities in other ruminants have similarly been found to be affected by high-concentrate diets, such as in goats [38], yak [39], bison [40], and sheep [41,42,43,44,45,46,47,48,49,50]. Overall, these studies have also indicated that changes in rumen bacterial community composition in response to feeding concentrate-based diets are at least in part due to the increase in the abundance of unknown or uncharacterized bacterial species from poorly defined phylogenetic lineages. This is consistent with previously published estimates that the majority of ruminal bacterial species have yet to be isolated or cultured [51,52]. Since their functions or metabolic capabilities remain unknown or poorly characterized, further investigations of novel ruminal microbial species that are favored under high-concentrate diets would yield valuable insights into the mechanisms involved in the adaptation of the rumen to easily fermentable diets.

In this context, this current report describes a comparative analysis of ruminal bacterial communities between lambs maintained on a forage-based diet and lambs that were fed a diet with a high inclusion of whole corn. Consistent with previously published studies from other groups, bacterial composition profiles were very different between the two treatments, and their respective microbial communities included distinct sets of uncharacterized species among their most abundant members. Using a metagenomics-based approach, the metabolic capabilities of three different groups of bacteria more prominently represented in concentrate-fed animals were further analyzed. As expected, the potential to hydrolyze starch and metabolize glucose into pyruvate was found in all three groups; however, each showed very distinct capabilities for other functions, such as the types of end products generated, the capacity for amino acid synthesis or transport, as well as the ability to use alternative substrates. Notably, the potential to produce formate and acetaldehyde indicated that lactate may not have been the only undesirable microbial end product generated by the most abundant bacterial species under these conditions. In contrast to humans, where microbial metabolism of acetaldehyde and ethanol is being actively investigated in the context of gut health, the impact of these microbial end products on the ruminal environment has yet to be explored, and it may represent an additional line of future inquiry into determining the impact of concentrate diets in ruminants. Together, the results from this study provide further insight into how ruminal bacterial communities in finishing lambs adapt to starch-based ingredients, and how they can in turn affect the rumen environment.

## 2. Materials and Methods

### 2.1. Feeding Trial and Sample Collections

Each rumen sample analyzed in this report was collected from an individual animal as part of a previously published sister study [53], with all procedures involving the use of animals approved by the South Dakota State University Institutional Animal Care and Use Committee (IACUC; Protocol #2101-004E). While the aim of the original trial was to investigate the potential effects of a feed additive on the performance of lambs during a ‘dietary grain challenge’, only rumen samples collected from control (non-supplemented) lambs were analyzed for bacterial composition in this study. Briefly, after 89 days on a high-forage-based diet, polypay wethers (castrated male sheep) were transitioned to a high corn-based diet for 63 days (‘dietary grain challenge’), with lambs allotted to either treatment or control pens; each dietary treatment consisted of 16 pens, with 5 wethers per pen. For the forage-based diet, the main dietary ingredients were oat hay (57.5%), grower pellets (28.6%; commercial formulation), and DDGS (15%). The main dietary ingredients in the high grain challenge diet consisted of whole corn (65.5%), grower pellets (28.6%; commercial formulation), pelleted soy hulls (5.0%) and soy oil (0.9%). Additionally, decoquinate was supplemented to the ration to prevent coccidiosis (Deccox^®^, 77.22 mg/kg; Zoetis Services LLC, Parsippany, NJ, USA) [53].

For the research described in this report, rumen samples collected before the diet transition (D = 0, *n* = 4; individuals selected were closest to the median weight of all lambs), as well as from the control group at days 56 (D56; *n* = 7) and 63 (D63; *n* = 8), were analyzed for bacterial composition. For sample collections, the digestive tract was removed post-slaughter from each carcass, and the rumen was separated to collect 50 mL aliquots of unfiltered contents, which were then frozen and stored at −80 °C until processing.

### 2.2. Microbial DNA Isolation and Sequencing of 16S rRNA Gene Amplicons

Microbial genomic DNA was isolated from individual rumen samples using the repeated bead beating plus column method, as previously described [54], with the QIAamp DNA Stool Kit (QIAGEN, Hilden, Germany) used for DNA purification. Purified microbial genomic DNA samples were subsequently submitted to Molecular Research DNA (MRDNA, Shallowater, TX, USA) for V1–V3 amplification of the bacterial 16S rRNA gene, followed by amplicon sequencing using an Illumina Miseq (2 × 300) platform to generate overlapping paired-end reads. The universal forward 27F-5′AGAGTTTGATCMTGCTCAG and reverse 519R-5′GWATTACCGCGCGCGCTG primers were used to target the V1–V3 region of the bacterial 16S rRNA gene; they represent optimized versions of the original 27F (5′AGAGTTTGATCMTGGCTCAG; [55]) and 519R (5′GWATTACCGCGGCKGCTG; [56]) primers.

### 2.3. Bacterial Composition Analyses

The bioinformatics procedure for bacterial composition analysis consisted of an in-house developed set of custom-written Perl scripts (Supplementary File S1), in combination with publicly available software, such as MOTHUR (v.1.44.3) [57] and BLASTn (v.2.14.1+) [58,59]. Using the command ‘make.contigs’ in MOTHUR, overlapping Miseq (2 × 300) paired-end reads generated from the same flow cell clusters were assembled into contigs to generate the sequences of V1–V3 amplicons that had been amplified from ruminal bacterial 16S rRNA genes. The resulting V1–V3 contigs were then screened to meet the following criteria: presence of both intact 27F (forward) and 519R (reverse) primer nucleotide sequences, a length between 400 and 580 nt, and an average Phred quality score of at least Q33. After quality screening, V1–V3 sequences were aligned using the conserved regions as reference points; this strategy was employed to ensure consistent positioning of variable regions. Aligned datasets were then clustered into Operational Taxonomic Units (OTUs) at a 4% sequence dissimilarity cutoff, using the most abundant sequence reads as initializing centroids (‘greedy’ approach). While 3% is most commonly used as a threshold for 16S rRNA gene clustering analyses, a standard that was originally determined with full-length 16S rRNA sequences (V1–V9), published reports by other groups have indicated that a higher percentage is more suitable for V1–V3 when assessing the extent of sequence variation within this subregion [60,61].

OTUs were then screened for artifacts using three independent approaches [62]. Chimeric sequences were first identified with the commands ‘chimera.uchime’ [63] and ‘chimera.slayer’ [64] in MOTHUR (v.1.44.3) [57]. Next, the 5′ and 3′ ends of OTUs were assessed using a database alignment search-based approach; OTUs were designated as artifacts if they were missing more than 5 nucleotides from the 5′ or 3′ end of their respective alignments when compared to their closest match of equal or longer length from the NCBI ‘nt’ database, as determined by BLASTn (v.2.14.1+); this threshold represents approximately 1% of the V1–V3 length. For OTUs with only one or two assigned reads, an additional screen was performed, where only sequences with a perfect or near-perfect match (maximum 1% of dissimilar nucleotides) to a sequence in the NCBI ‘nt’ database were considered valid. All OTUs and their assigned reads that were flagged as artifacts as a result of these screens were removed from further analysis.

Taxonomic assignment of curated OTUs to phylum and family-level affiliations was performed using ‘classify.seqs’, a MOTHUR implementation of RDP Classifier [65]. Closest valid relatives for select OTUs were identified by searches with BLASTn against the ‘refseq_rna’ database [58]. The alpha diversity indices ‘Observed OTUs’, ‘Ace’, ‘Chao’, ‘Shannon’, ‘Simpson’, and ‘Sobs’ were calculated using the ‘summary.single’ command in MOTHUR (v.1.44.3) [57]. For beta diversity, Bray-Curtis distances were first calculated using the ‘summary.shared’ command, followed by ‘pcoa’ for Principal Coordinate Analysis (PCoA) (MOTHUR, v.1.44.3). For both alpha and beta diversity analyses, rarefied OTU tables of 6400 sequences per sample were created by subsampling from the original datasets using custom Perl scripts.

### 2.4. Metagenomic Assembly and Analyses

Purified microbial genomic DNA preparations from three lambs fed the high corn inclusion diet (RA2, RA6 and RA8; Supplementary File S2) were used as templates for ‘shotgun’ sequencing with an Illumina Miseq (2 × 250) platform (Molecular Research DNA, Shallowater, TX, USA). These samples were selected based on their OTU composition, as high representation of select OTUs of interest would favor increased sequencing coverage and assembly of longer contigs. Unless specified, metagenomic contig assembly steps and analyses were performed using a set of in-house developed Perl scripts (Supplementary File S1). Individual raw datasets were filtered to select sequence reads with a length of at least 200 bp, which were subsequently used to build contigs [66]. Assembled contigs were then annotated with the online implementation of the ‘Rapid Annotations using Subsystem Technology’ (RAST; [67]) to identify putative coding sequences, their corresponding amino acid sequences, as well as their respective predicted functions. A custom BLASTp-based approach was subsequently used to cluster contigs into bins according to the taxonomic affiliation of their coding sequences. Briefly, the translated amino acid sequences encoded in each contig were compared by BLASTp to the proteomes of representative valid species affiliated to 16 bacterial families that are commonly identified in rumen and gut environments (Supplementary File S3). As species affiliated to the same phylogenetic lineage would be expected to have a higher number of homologous genes in common compared to species of unrelated taxonomic groups, individual contigs were assigned to a family-level bin according to the number of homologous genes that they shared with representative species of that family. Coding sequences were deemed homologs if they met thresholds of 50% for amino acid sequence identity and 80% for alignment length [66]. Bins with at least 1500 coding sequences were further analyzed by assessing their metabolic capabilities through identification of putative pathways and the potential for carbohydrate utilization. The potential for encoded metabolic pathways was assessed by matching predicted enzymes from RAST annotations to reference pathways available from the Kyoto Encyclopedia of Genes and Genomes (KEGG) database [68]. Putative enzymes involved in carbohydrate utilization (Carbohydrate-Active enZymes or CAZy) were identified using the online implementation of the ‘automated carbohydrate-active enzyme and substrate annotation’ (dbCan3; https://pro.unl.edu/dbCAN2/ accessed on 6 July 2026) [69]; a hit was deemed valid if it was independently identified as a positive match by all available tools (HMMER: e-value < 1 × 10^−15^, coverage > 0.35; dbCAN-sub: e-value < 1 × 10^−15^, coverage > 0.35; DIAMOND: e-value < 1 × 10^−102^).

### 2.5. Statistical Analyses

Statistical tests were conducted using R (version R-4.3.0). For alpha diversity indices, comparisons across groups were performed using ANOVA (command ‘aov’), followed by Tukey’s range test for multiple comparisons (command ‘TukeyHSD’); normality of distribution for alpha indices was confirmed using the Shapiro–Wilk normality test (command ‘shapiro.test’). For statistical testing of taxonomic groups and the most abundant OTUs, the non-parametric test Kruskal–Wallis (command ‘kruskal.test’) was used, followed by the Wilcoxon test (command ‘pairwise.wilcox.test’), which included the Benjamini–Hochberg correction for controlling the false discovery rate for multiple pairwise comparisons. To control the proportion of false positives in multiple statistical testing, adjusted *p* values for false discovery rate were calculated using the Benjamini-Hochberg procedure (command ‘p.adjust’). For all statistical tests, a threshold of *p* ≤ 0.05 was considered significant.

### 2.6. Next Generation Sequencing Data Accessibility

Raw sequence data are available from the NCBI Sequence Read Archive under project PRJNA1499784.

## 3. Results

### 3.1. Taxonomic Composition Analysis

The bacterial profiles of rumen samples collected from lambs fed a forage-based diet (*n* = 4) or a high corn diet at two different timepoints (D56, *n* = 7; D63, *n* = 8) were determined. Across 19 samples, a combined total of 607,856 high-quality sequence reads were generated for this analysis, with a median of 32,118 sequence reads per sample (Supplementary File S2). A taxonomy-based analysis identified *Bacillota* as the most abundant phylum across all samples, with eight of its affiliated families found to be highly represented (Table 1). While the two most abundant *Bacillota* families, *Lachnospiraceae* and *Oscillospiraceae*, were not found to be statistically different across the different treatment groups, the respective profiles of the remaining six prominent families were found to vary (*p* < 0.05). Notably, *Erysipelotrichaceae*, *Lactobacillaceae* and *Eubacteriaceae* were found to be highest in both corn-fed groups compared to the high forage-fed group, while members of the *Acidaminococcaceae* family showed an opposite abundance profile (*p* < 0.05). Of the other well-represented phyla that were analyzed, *Planctomycetota* and *Chloroflexota* were found to be higher in the rumen samples collected from forage-fed lambs (*p* < 0.05).

### 3.2. Alpha and Beta Diversity Analyses

As taxonomy-based profiles indicated major differences in ruminal bacterial composition between forage- and corn-fed lambs, alpha and beta diversity analyses were conducted to gain more insight. Ruminal bacterial diversity, as assessed with the indices ‘observed OTUs’, ‘ace’ and ‘chao’, was found to be higher in samples from the forage-fed group (*p* < 0.05; Table 2). Evenness, an indicator of community structure that was assessed by the Shannon and Simpson indices, was similarly found to be different between the respective samples from the forage-fed group and the corn-fed lambs (*p* < 0.05; Table 2). Accordingly, analysis of beta diversity supported major differences at the community level between forage and corn-based diets, with clear clustering and separation of samples into distinct groups by PCoA (Figure 1).

### 3.3. OTU Composition Analysis

To further investigate the differences in ruminal bacterial community structure from feeding the diets under study, an OTU-level composition analysis was performed. A total of 8986 Operational Taxonomic Units (OTUs) were identified across all samples (Supplementary File S2), from which the most abundant (defined as represented by a median of at least 1.0% in at least one group) were analyzed on an individual basis (Table 3). Overall, the profiles of abundant OTUs also supported major differences in ruminal bacterial community structure between forage-fed and corn-fed lambs, with 14 of these OTUs found at different levels between the two diets. More specifically, six of the most abundant OTUs (Oa-00024, Oa-00044, Oa-00049, Oa-00053, Oa-00056 and Oa-00062) were more highly represented in forage-fed lambs (*p* < 0.05), while eight other abundant OTUs (Oa-00003, Oa-00004, Oa-00005, Oa-00011, Oa-00012, Oa-00015, Oa-00016 and Oa-00018) were at higher levels in corn-fed lambs (*p* < 0.05).

### 3.4. Investigating the Metabolic Potential of Uncharacterized Ruminal Bacterial Species from Sheep

Further analyses revealed that five of the most highly represented OTUs identified in this study showed at least 97% or greater sequence identity to the 16S rRNA gene of a valid bacterial species (Table 3), indicating that they may have been strains of known microbial species. Conversely, the remaining 15 OTUs likely corresponded to unknown or novel species, as their V1–V3 regions were very distant from their respective closest valid relatives, with nucleotide sequence identities ranging between 81.6% and 95.4% (Table 3). As uncharacterized bacterial species may possess metabolic capabilities that can help further understand how the rumen environment of lambs adjusts to high corn diets, a metagenomics approach was used to assess their genetic potential. To this end, samples from corn-fed lambs were selected based on their respective OTU profiles for the assembly of partial bacterial genomes of uncharacterized ruminal bacterial species in sheep. From the contigs generated using this approach, three different sets defined based on their respective taxonomic affiliations were analyzed in further detail.

#### 3.4.1. Metabolic Capability Predictions for Oa-00002 (*Oscillospiraceae*)-Affiliated Contigs

This contig set was assembled primarily from sequence data generated from a sample in which the most highly represented OTU, Oa-00002 (36.5%), was affiliated with *Oscillospiraceae*. It was inferred that this contig set belonged to Oa-00002, since the abundance of this OTU was 27.5X greater than the levels of the next most abundant OTU affiliated with *Oscillospiraceae* in this sample (Oa-00016, 1.3%). A total of 2284 unique coding sequences were identified, of which 688 could be annotated to known protein functions, and 1021 were identified as ‘hypothetical’. Based on the number of ribosomal proteins found in the contig set, genome completion was estimated at 92.6% (small subunit: 18/21 identified, missing S6p, S18p and S21p; large subunit: 32/33 identified, missing L25p). Notably, this contig set also included a full-length copy (1488 bp) of a 16S rRNA gene corresponding to Oa-00002, as its V1–V3 region was a perfect match (100% sequence identity) to this OTU (Supplementary File S2).

The bacterial species corresponding to Oa-00002 was predicted to specialize in the utilization of starch, with the identification of a set of hydrolytic enzymes whose combined activities would result in the release of glucose from amylose and/or amylopectin (Figure 2). From a survey of carbohydrate-active enzymes, no other type of polysaccharides or glucans could be identified as additional potential substrates (Supplementary File S3). Predicted coding sequences for subunits of a Phosphoenolpyruvate-phosphotransferase system (PTS; EC 2.7.3.9) indicated the capacity to acquire and phosphorylate glucose. The resulting intracellular glucose-6P could then be metabolized into pyruvate by glycolysis (Embden-Meyerhof-Parnas pathway), based on the identification of coding sequences for the majority of enzymes needed to catalyze the expected steps of the pathway (7/9), with only fructose-1,6-bisphosphate aldolase (EC 4.1.2.13) and enolase (EC 4.2.1.11) not found to be encoded in the contig set. Coding sequences for pyruvate formate lyase (EC 2.3.1.54) and its activating enzyme (EC 1.97.1.4) indicated that the main expected end products from breaking down pyruvate would be acetyl-CoA and formate [70] (Figure 2). Acetyl-CoA could be further metabolized into acetate, one of the major SCFAs produced from the ruminal fermentation of feed that can be utilized by the host animal as a source of energy (Figure 2). A likely outcome for formate would be transport to the extracellular environment, through the activity of a predicted FocA-type formate efflux transporter (Figure 2).

With regard to the regeneration of NADH produced during glycolysis into NAD^+^, three potential mechanisms were predicted from the Oa-00002/*Oscillospiraceae* contig set (Figure 2; Supplementary File S3). One would involve production of H_2_ by hydrogenase–ferredoxin complexes, while another would consist of generating acetaldehyde through reduction of acetyl-CoA by acetaldehyde dehydrogenase (EC 1.2.1.10). A third mechanism could involve the activity of an Rnf complex, as five of the six necessary subunits were identified in the Oa-00002 contig set; RnfG was the only subunit that was not found. Based on results from other studies, NADH oxidation by the Rnf complex could be coupled to the export of Na^+^ ions [71,72], which would result in an extracellular gradient that could be used as an ion motive force. Based on gene annotation analysis of the contig set, possible functions of a Na^+^ gradient could be for phosphate transport (ABC transporter TC 3.A.1.7.1; all subunits identified: PstA, PstB, PstC and PstS), as well as to act as a source of energy for ATP production by ATP synthase; coding sequences for all expected nine subunits of a V-type ATP synthase were identified. Other predicted functions of interest included the transport of iron, which is necessary for the activity of hydrogenases and the Rnf complex.

The potential of Oa-00002 to either synthesize amino acids or acquire them from the extracellular environment was also investigated. Based on gene annotation, predicted transport capabilities were found for glutamine (three subunits of a glutamine/basic amino acid ABC transporter), methionine (three subunits of a methionine ABC transporter), and branched-chain amino acids (subunits LivF, LivG, LivH, LivJ and LivM of a TC 3.A.1.4.1-type ABC transporter) (Supplementary File S3). L-glutamine could be used as a starting substrate for the synthesis of L-glutamate, L-proline, and L-arginine, with coding sequences found for all but two enzymes predicted to be needed for these functions (Supplementary File S4). L-aspartate, a required precursor for the synthesis of L-asparagine, L-lysine and L-threonine by this candidate bacterial species (Supplementary File S4) was predicted to be generated by the activity of aspartate aminotransferase (EC 2.6.1.1) using glutamate and oxaloacetate as substrates, with the latter potentially produced from the glycolysis intermediate phosphoenol pyruvate (PEP). Similarly, the amino acids L-cysteine, L-serine, L-glycine and L-tryptophan could be synthesized by a pathway starting with the glycolysis intermediate 3-phospho-D-glycerate, and a downstream step that would involve a phosphoserine aminotransferase (EC 2.6.1.52), with glutamate as an amino group donor (Supplementary File S4). As the Oa-00002/*Oscillospiraceae*-affiliated contig set included enzymes of the shikimate pathway, as well as enzymes required to generate L-phenylalanine (1/2 enzymes) and L-tyrosine (2/2 enzymes) from prephenate, Oa-00002 would also have the metabolic capability to synthesize these aromatic amino acids (Supplementary File S4). The contig set did not, however, provide sufficient information to determine how Oa-00002 would synthesize and/or acquire L-alanine and L-histidine, respectively.

#### 3.4.2. Metabolic Capability Predictions for *Lachnospiraceae*-Affiliated Contigs

From the *Lachnospiraceae* contig set, a total of 1621 protein coding sequences were identified (Supplementary File S3). Specific contigs could not be reliably assigned to distinct OTUs, because the samples analyzed by metagenomics included multiple *Lachnospiraceae* OTUs with similar abundance levels, with the six most highly represented OTUs from this group ranging between 1.1 and 4.6% (Oa-00004, Oa-00007, Oa-00018, Oa-00020, Oa-00040 and Oa-00041). Since functions could not be assigned to specific OTUs with sufficient accuracy for this contig set, predicted metabolic capabilities were analyzed as a group.

Based on an assessment of CAZy enzymes encoded in this dataset, starch was predicted to be the main substrate for bacterial species of this group, with potentially the capacity to also utilize pectin, as a gene encoding a putative α-L-arabinofuranosidase was identified. In contrast to Oa-00002 (*Oscillospiraceae*), also a candidate starch utilizer as described above, *Lachnospiraceae*-affiliated bacteria would have the potential to transport maltodextrin through an ABC-type transporter, after being released from amylose by the hydrolytic activity of neopullulanases. While coding sequences for only three enzymes from glycolysis were found, this pathway is presumed to be active in species represented in the *Lachnospiraceae* contig set, as two enzymes that can metabolize pyruvate, the end product of glucose fermentation by glycolysis, were identified. One possible outcome could involve the transformation of pyruvate into formate and acetyl-CoA by pyruvate formate lyase (EC 2.3.1.54), as was described for Oa-00002. Coding sequences for acetyl-CoA synthetase (EC 6.2.1.1) indicated the potential use of acetyl-CoA as a substrate to generate ATP from AMP and pyrophosphate [73]; this reaction would also be beneficial to the host, as it would generate acetate as an end product. Notably, the identification of coding sequences for pyruvate phosphate dikinase (EC 2.7.9.1) in this contig set provided an additional potential mechanism for ATP production from AMP by substrate-level phosphorylation (Supplementary File S4). The other predicted outcome for pyruvate would be its transformation into D-lactate by D-lactate dehydrogenase (EC 1.1.1.28), which would result in oxidation of NADH into NAD^+^, an essential mechanism for regeneration of reduction potential needed to maintain the activity of the glycolytic pathway. Identification of genes encoding subunits of a putative hydrogenase complex (HypA, HypB, HypD, HypE) provided an alternative mechanism for oxidation of NADH. Intriguingly, the *Lachnospiraceae* contig set also included a putative gene encoding an alcohol dehydrogenase, whose predicted function would be to transform acetaldehyde into ethanol, thereby also contributing to the regeneration of NADH into NAD^+^. Other notable metabolic capabilities of the *Lachnospiraceae* contig set included coding sequences for an ABC-type oligopeptide transporter (subunits OppA, OppB, OppC, OppD, OppF), suggesting that bacterial species from this group may acquire amino acids through proteolysis of proteins from feed or other sources that would be available in the rumen environment.

#### 3.4.3. Metabolic Capability Predictions for *Prevotellaceae*-Affiliated Contigs

From the *Prevotellaceae*-affiliated contigs, 397 of the 1508 coding sequences identified could be assigned to known metabolic functions (Supplementary File S3). Since the four most abundant *Prevotellaceae*-affiliated OTUs were present within a narrow abundance range (1.4–6.0%; Oa-00005, Oa-00011, Oa-00012, and Oa-00019), contigs from this set could not accurately be assigned to specific OTUs. Overall, the main distinctive feature of this contig set compared to *Oscillospiraceae* and *Lachnospiraceae*-affiliated bacteria identified in this study was a much greater diversity of coding sequences for CAZy, indicating the potential to metabolize a broad array of substrates in addition to starch. Indeed, *Prevotellaceae*-affiliated ruminal bacteria represented by this contig set were predicted to utilize different types of hemicellulose, such as xylans and mannan, as well as pectins and host glycans (Supplementary File S3). In addition, coding sequences for complexes involved in maintaining ion gradients were also identified, including a Na^+^-translocating NADH-quinone reductase (EC 1.6.5.8; all subunits, A-F, identified), as well as a NADH ubiquinone oxidoreductase (subunits A-C and subunits H-N were found, but coding sequences for subunits D-G could not be identified in the contig set). Ion gradients could be used for functions such as energy metabolism and transport, as would be required by a Na^+^/H^+^ dicarboxylate symporter that was predicted from gene annotation of this contig set.

## 4. Discussion

In ruminant production systems, a common strategy consists of feeding concentrate-based diets during the finishing phase. These typically contain elevated levels of corn, grain or other ingredients with a high content of easily digestible polysaccharides such as starch. As their conversion into SCFAs is faster, they allow faster gains and increased animal performance. Since ruminant grazers such as sheep have evolved to digest plant biomass that consists primarily of structural polysaccharides, feeding concentrate-based diets can result in health issues, inadequate rumen development, and slow growth, which can impact their future performance overall [74]. In sheep, poor diet formulations are thought to be responsible for various metabolic disorders, including fatty liver and ketosis, in addition to SARA and acidosis [75]. As problems tend to be more severe during the transition from forage to concentrate diets, a common strategy consists of gradually increasing the proportion of grain in the diet over an adaptation period, rather than abruptly transitioning from high fiber to high concentrate. While this strategy mitigates the risks of health problems, it requires animals to be on feed for a longer period, which delays when market weight or peak performance can be reached, thereby incurring additional costs. In this context, developing effective strategies to eliminate the need for gradual dietary transitions or reduce the adaptation time would be very beneficial to producers.

In contrast to abiotic anoxic environments, where biomass can be completely degraded into CO_2_ and methane, fermentation of organic substrates from feed peaks with the production of SCFA because of the faster turnover rates in gut environments [76,77]. This is of great benefit to animal hosts, as they can absorb and utilize these nutrients before they are further metabolized by microbial communities [17]. While SCFA production contributes to lowering gut pH, the accumulation of other microbial end products with a lower pKa, such as formate or lactate [78], can have a greater impact. Indeed, as these other end products are not readily absorbed or utilized by the host animal, alternative mechanisms, such as cross-feeding, are needed to keep their levels under control. Well-documented examples of microbial species that can use undesirable end products as substrates include *Megasphaera elsdenii*, a lactate utilizer that can produce propionate [79,80], as well as methanogens, which can metabolize formate into methane. As microbial species specialized in utilizing starch are more likely to generate lactate or formate compared to symbionts that metabolize structural polysaccharides, feeding high-concentrate diets favors synthesis and higher accumulation of these end products. In this context, a benefit of step-wise increases in starch content when transitioning from forage to concentrate is to allow populations of cross-feeding species to proliferate, ultimately building the capacity to control the levels of low pKa microbial end products that are generated when full concentrate diets are fed. Considering that most ruminal bacterial species are unknown and have yet to be characterized [51,52], sustained research efforts are required to uncover the range of metabolic capabilities that are favored in the adaptation of ruminal microbial communities to starch-rich diets.

### 4.1. Formate and Acetaldehyde Production by Ruminal Bacterial Starch Utilizers in Finishing Lambs

To this end, the study described in this report identified previously uncharacterized ruminal bacterial species that were enriched when a concentrate-based diet with a high inclusion of whole corn was fed to finishing lambs. While the design of the study did not allow for separation of the respective potential impact of other factors such as age, time on feed or physiological status, the predicted metabolic capabilities of prominent bacteria indicated that diet composition had an important effect on bacterial composition. For instance, the capacity to hydrolyze starch was a shared feature among the main bacterial groups that were further analyzed by metagenomics. Notably, coding sequences for Neopullulanase (EC 3.2.1.135) were found in all three contig sets analyzed; as this CAZy can hydrolyze both α-1,4 and α-1,6 glycosidic bonds [81,82], it would provide the necessary enzymatic activity to utilize amylose and amylopectin polymers stored as starch in whole corn. Each contig set also included glycolysis enzymes, supporting the potential to metabolize glucose through this pathway. While not all expected enzymes were identified in the contig sets (Oa-00002: 7/9; *Lachnospiraceae*: 3/9), the capacity to metabolize glucose by glycolysis was assumed to be encoded in the respective genomes of the corresponding species, because each contig set included at least one enzyme predicted to metabolize pyruvate, the end product of glycolysis. However, future research will be required to determine whether the changes in bacterial composition were a direct effect of the high corn diet; the results presented in this report should therefore be interpreted as associations with the transition to and subsequent exposure to the high-corn diet.

Two main potential outcomes were predicted for pyruvate. One would be the production of lactate, as supported by the identification of coding sequences for lactate dehydrogenase enzymes in the *Lachnospiraceae* and *Prevotellaceae* contig sets; this is consistent with this type of enzyme being present in bacterial genomes across different phylogenetic lineages [83]. The other predicted outcome for pyruvate would be its transformation into acetyl-CoA and formate; this capability was found in the Oa-00002 (*Oscillospiraceae*) and *Lachnospiraceae* contig sets, but not in the partial genome assemblies generated from *Prevotellaceae*. Acetyl-CoA could be further metabolized into acetate (Oa-00002 and *Lachnospiraceae*) or acetaldehyde (Oa-00002). Since coding sequences for acetate transporters or permeases were not identified, passive diffusion of acetic acid (generated from protonation of acetate) would be the most likely mechanism for acetate to be released into the rumen [84,85]. As a small uncharged molecule with a slight polarity, acetaldehyde could also be transferred directly into the rumen environment by passive diffusion through bacterial membranes. In contrast, two potential cell-mediated mechanisms were identified for formate. Expression of an efflux transporter encoded in the Oa-00002 genome assemblies would allow export of this compound to the rumen environment, likely at rates anticipated to be greater than by passive diffusion [86,87,88,89], while a *Lachnospiraceae*-encoded formate dehydrogenase enzyme could metabolize formate into CO2, providing a mechanism to eliminate this compound from the system.

### 4.2. Putative Cross-Feeding Mechanisms for Undesirable Microbial End Products

As described in earlier sections, undesirable microbial end products, which include compounds that are not effectively utilized by the host or that can be detrimental to its health, need to be eliminated or neutralized by other mechanisms. Lactate is likely the most studied end product in this category, as its accumulation in the rumen is associated with severe acute lactic acidosis. Based on the predicted metabolic potential of their corresponding bacterial species, OTUs Oa-00004, Oa-00009, Oa-00011, and Oa-00015 represent potential starch utilizers and lactate producers (Table 3; Supplementary File S5), as supported by the identification of putative coding sequences for both Neopullulanase (EC 3.2.1.135) and lactate dehydrogenase enzymes in each respective genome. Considering the extent of previously reported research spanning more than a decade on *M. elsdenii*, this species is considered one of the best-characterized lactate utilizers in gut environments. Indeed, promoting a higher abundance of this microorganism is a well-documented strategy to mitigate or control lactate accumulation through cross-feeding [90], as is the development of strains for use as probiotics; these topics have recently been reviewed in greater detail [79,80]. With a level of sequence identity higher than 99%, Oa-00045 was the most highly represented OTU found to be closely affiliated with *M. elsdenii* in this current study. Oa-00045 levels varied extensively across concentrate-fed lambs (Supplementary File S2), with a range of 0.003–2.28% in abundance. Compared to Oa-00045 levels in forage-fed lambs (0.005–0.013%), which had a comparatively limited intake of starch, it appeared that levels of *M. elsdenii* did not increase consistently across lambs that were fed the same diet with a high inclusion of whole corn. Future research will be required to determine whether these differences in *M. elsdenii* abundance were due to varying responses to high-starch diets among ruminal communities or if other lactate utilizers may have been involved, such as *Pseudoselenomonas ruminantium var. lactilytica* [91] or uncharacterized species [66,92].

Despite having been identified early as an end product of ruminal fermentation [93], formate has received considerably less attention than lactate. Considering that its pKa is lower than the pKa of lactate (3.5 vs 3.9 [94]), formate may represent a greater risk of acidifying ruminal pH. In this study, coding sequences for pyruvate formate lyase were identified in two experimental contig sets (Oa-00002 and *Lachnospiraceae*; Supplementary File S3), as well as in the genomes of bacterial species matched to OTUs that were abundant in the rumen of concentrate-fed lambs (Oa-00004, Oa-00009, and Oa-00011; Supplementary File S5). Further gene annotation analyses revealed the potential of Oa-00002 and Oa-00004 to express FocA efflux transporters (Supplementary Files S3 and S5), which would be expected to exacerbate this effect by increasing the transfer of formate to the extracellular environment [88,89]. In rumen environments, the main mechanism known to metabolize formate is through cross-feeding with methanogens, which they can use as a substrate to produce methane [16,93]. While acetogens represent another well-characterized group of formate utilizers in gut environments [95], they appear to be more prominent in the hindgut [96] compared to the rumen, where proposed candidate acetogens were reported to also have the capacity to produce formate as an end product of polysaccharide breakdown [97].

As of submission of this report, very limited information is available on the potential effects of acetaldehyde, a predicted end product of Oa-00002, on rumen function. In contrast, the impact of this compound on human health has made it a focus of very active research in humans, not just in host organs but also in gut environments, because of its association with diabetes and alcohol use [98]. For instance, a study based on simulations using reconstruction of genome-scale metabolic models identified acetaldehyde as one of the most exchanged compounds among bacterial species in fecal samples collected from women with Type 2 diabetes [99]. These findings complement previously reported experimental evidence that used in vitro culturing methods to show cross-feeding interactions by human gut bacteria involving acetaldehyde [100]. The recent development of a probiotic designed to prevent accumulation of acetaldehyde in the human gastrointestinal tract further emphasizes the importance of this cross-feeding interaction as a mechanism for maintaining gut health [101]. In this context, results from the current study support cross-feeding interactions involving acetaldehyde in the rumen of lambs fed a whole corn diet. For instance, the genome of *Succinivibrio dextrinosolvens*, the closest valid relative of OTU Oa-00009, includes coding sequences for alcohol dehydrogenase, but it does not encode acetaldehyde dehydrogenase or aldehyde dehydrogenase enzymes (Figure 3 and Supplementary File S5). Since this species does not appear to have the capability to produce acetaldehyde, the presence of coding sequences for alcohol dehydrogenase could be indicative of cross-feeding. In this context, one possible advantage of transforming acetaldehyde into ethanol could be as an alternative mechanism for regeneration of NADH into NAD^+^. Similarly, coding sequences for alcohol dehydrogenase, but not for acetaldehyde dehydrogenase or aldehyde dehydrogenase, were identified in the *Lachnospiraceae* contig set (Supplementary File S3). This would represent an example of a mutually beneficial cross-feeding relationship among different ruminal bacterial species, where regeneration of NADH to NAD^+^ would contribute to mitigating acetaldehyde levels in the rumen. Conversion of acetaldehyde into ethanol appeared to be a mechanism used by other bacterial OTUs predicted to generate acetaldehyde as an intermediate, such as Oa-00004 (*Chordicoccus furentiruminis*) and Oa-00015 (*Limosilactobacillus mucosae*) (Figure 3). These predictions are consistent with early reports that ethanol was a product of ruminal fermentation in cattle and sheep that were fed high amounts of easily fermentable carbohydrates [102]. Notably, accumulation of ethanol in the rumen would be another factor affecting ruminal bacterial composition, since this compound has been found to inhibit the growth of *S. ruminantium* and *Butyrivibrio fibrisolvens* under in vitro conditions [103]. Together, these results indicate that cross-feeding involving other end products in addition to lactate should be further investigated in rumen environments. In order to assess the impact of formate, acetaldehyde and ethanol on the ruminal environment, future studies would benefit from including quantification of their concentrations in addition to measuring SCFA and lactate levels.

## 5. Conclusions

As they can account for up to 70% of total direct costs [104], feed expenditures are a common challenge faced by livestock producers. Feeding high-quality or energy-dense diets only during peak growth periods is a commonly used strategy that can help mitigate feed-related production costs in ruminants. As microbial fermentation precedes host digestion in ruminants, this process is particularly impactful on efficient use of feed. Since gut microbial communities consist of metabolic specialists that each utilize specific compounds in feed that the host animal is unable to digest [105], feed formulation acts as a major selection factor in determining their composition. Feeding concentrate or grain-based diets that are rich in starch can favor the growth of microorganisms that generate undesirable end products, in addition to greater concentrations of SCFAs and other nutrients. While the effects of lactate on ruminal pH and physiology have been extensively studied, less attention has been given to the potential impact of other microbial end products such as formate, ethanol or acetaldehyde. The results described in this report indicate that unknown or poorly characterized ruminal starch utilizers may negatively impact the rumen environment through production of these other end products. However, the results also indicate that abundant bacterial species favored under these conditions could be capable of complementary metabolic activities that would allow for mitigating levels of undesirable end products through cross-feeding. In the context of adapting the rumen biological and chemical environments to high-starch diets, this information may help in the future development of improved strategies to reduce the transition period during the finishing phase, as well as mitigate health issues that may compromise growth performance.

## Figures and Tables

**Figure 1:**
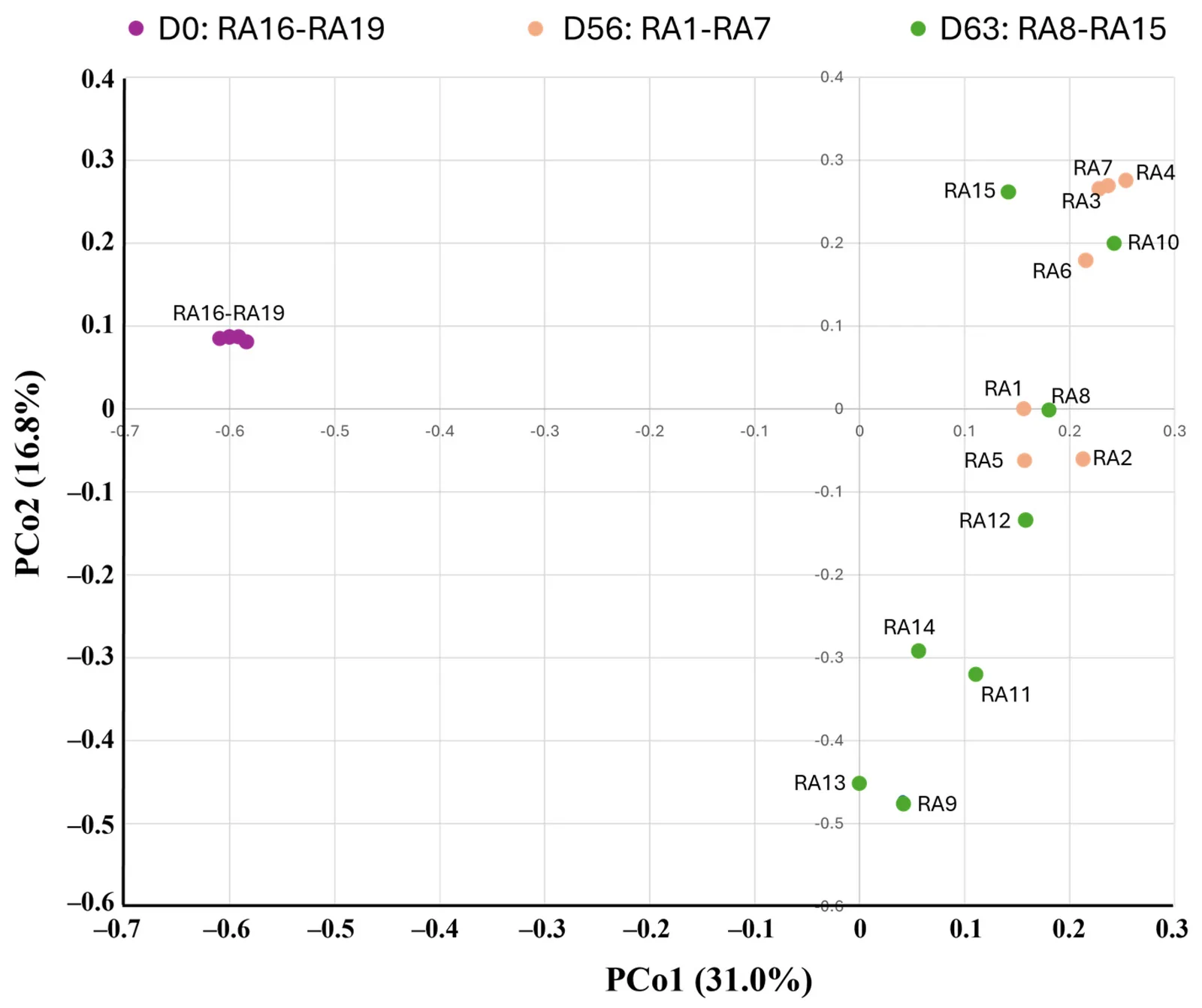
Principal coordinate analysis (PCoA) of ruminal bacterial communities from lambs fed either a forage-based diet (D0, *n* = 4) or a whole corn-based diet at two different time points (D56, *n* = 7; D63, *n* = 8). The x- and y-axes correspond to principal coordinate 1 (PCo1) and principal coordinate 2 (PCo2), respectively. For detailed OTU composition of each sample, please refer to Supplementary File S2.

**Figure 2:**
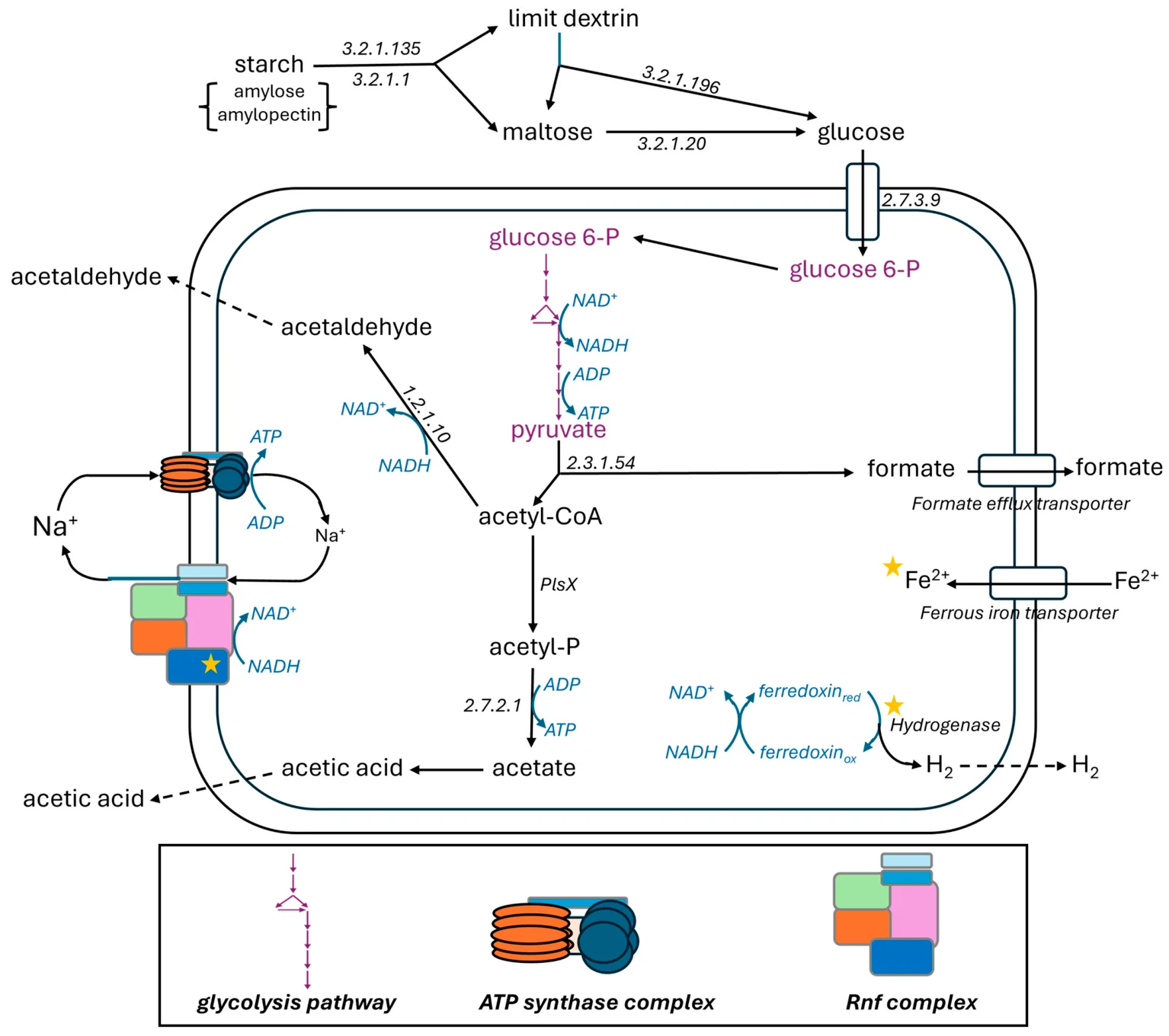
Proposed metabolic pathways for starch utilization, generation of end products, and ATP production by OTU Oa-00002. Movement by passive diffusion is represented by broken lines. Enzymes and protein complexes requiring iron as a co-factor are labeled with a yellow star. Codes shown represent the Enzyme Commission (EC) numbers of their corresponding enzymes.

**Figure 3:**
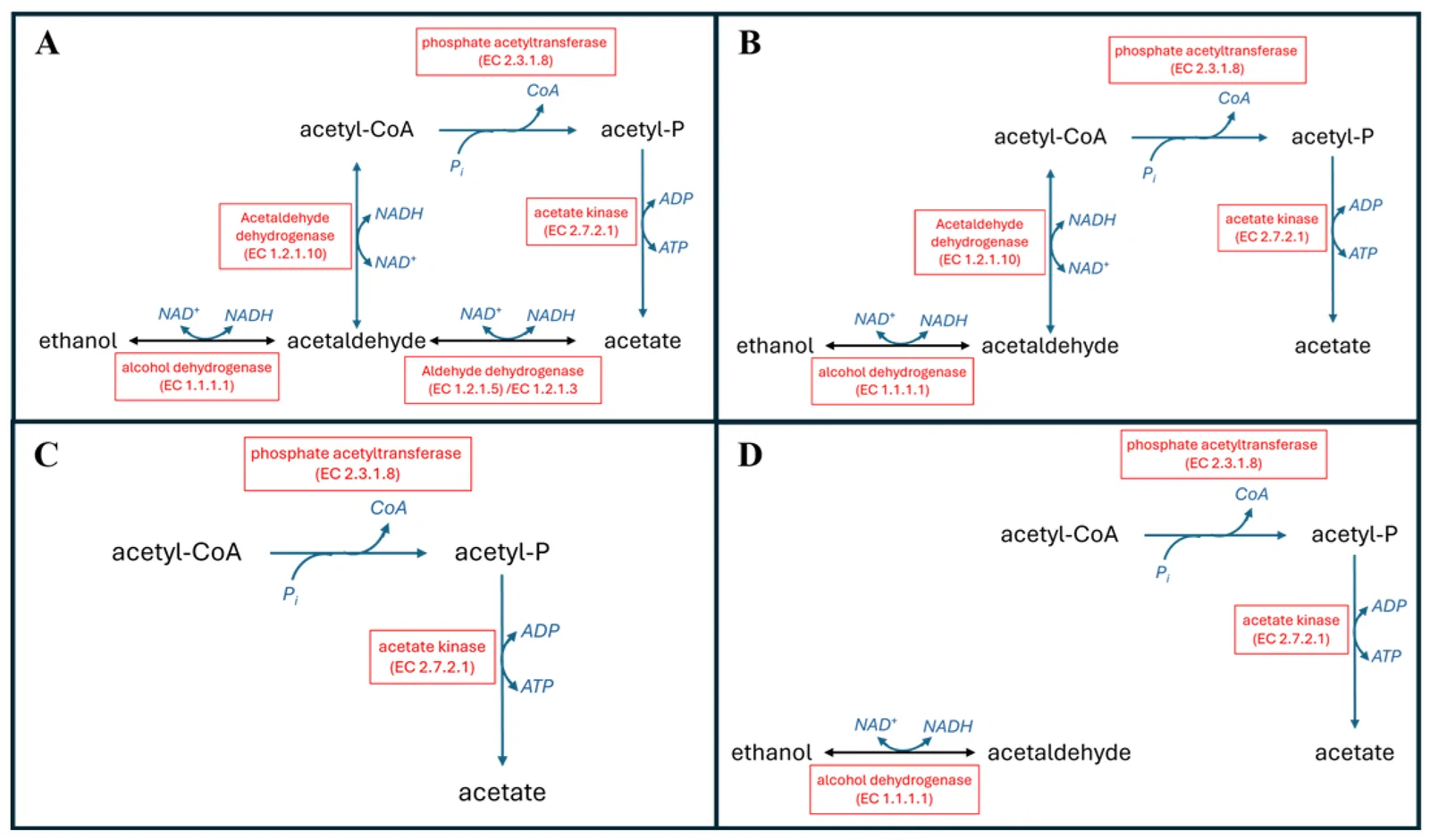
Proposed pathways for production of acetate, acetaldehyde and ethanol by OTUs (**A**) Oa-00004 (*Chordicoccus furentiruminis*), (**B**) Oa-00015 (*Limosilactobacillus mucosae*), (**C**) Oa-00011 (*Hallella mizrahii*), and (**D**) Oa-00009 (*Succinivibrio dextrinosolvens*). Predictions were based on gene annotation from publicly available genome sequences of each OTU’s respective closest relative. Please refer to Supplementary File S5 for additional details on genome accession numbers, genome selection criteria and annotation methods.

**Table 1:** Median of relative abundance (%) for the main bacterial taxonomic groups identified in the rumen of lambs that were fed either a forage-based diet or a whole corn-based diet. Phylum-level groups are highlighted in bold, with their respective family-level groups shown in regular font. Forage (D0): *n* = 4; Corn (D56): *n* = 7; Corn (D63): *n* = 8.

Taxa	Forage (D0)	Corn (D56)	Corn (D63)
**^1^ ** ***Bacillota*** ** ^$^**	**57.0**	**58.4**	**83.6**
*Lachnospiraceae*	21.1	17.1	19.9
^2^ *Oscillospiraceae*	14.3	2.2	21.1
*Erysipelotrichaceae*	0.6 ^a^	3.4 ^b^	10.8 ^b^
*Veillonellaceae*	0.4 ^a^	13.7 ^b^	2.5 ^ab^
*Lactobacillaceae*	0.1 ^a^	2.2 ^b^	6.5 ^b^
*Clostridiales* Incertae Sedis XIII	4.6 ^a^	0.3 ^b^	1.3 ^ab^
*Eubacteriaceae*	<0.1 ^a^	0.5 ^b^	1.5 ^b^
*Acidaminococcaceae*	1.2 ^a^	0.3 ^b^	0.1 ^c^
^#^ Other *Bacillota*	14.6	1.4	1.6
**^3^** ***Bacteroidota***	**24.1**	**22.5**	**10.9**
*Prevotellaceae*	15.2	21.8	10.4
^#^ Other *Bacteroidota*	7.9	0.5	0.7
**^4^** ***Pseudomonadota***	**0.6 ^a^**	**5.4 ^b^**	**0.3 ^c^**
*Succinivibrionaceae* ^$^	<0.1	1.8	0.1
^#^ Other *Pseudomonadota*	0.5	1.4	0.2
**^5^** ***Actinomycetota***	**0.8 ^ab^**	**0.7 ^a^**	**2.6 ^b^**
**^6^** ***Planctomycetota***	**1.0 ^a^**	**<0.1 ^b^**	**<0.1 ^b^**
**^7^** ***Chloroflexota***	**0.9 ^a^**	**<0.1 ^b^**	**<0.1 ^b^**
**^#^** **Other Phyla**	**1.9**	**<0.1**	**0.1**
**^#^** **Unclassified Bacteria**	**13.6**	**3.7**	**1.3**

^1^ formerly known as Firmicutes. ^2^ formerly known as *Ruminococcaceae*. ^3^ formerly known as Bacteroidetes. ^4^ formerly known as *Proteobacteria*. ^5^ formerly known as *Actinobacteria*. ^6^ formerly known as *Planctomycetes*. ^7^ formerly known as *Chloroflexi*. ^a,b,c^ Values with different superscripts were found to be significantly different by the Wilcoxon test (*p* ≤ 0.05). ^$^ Groups were found to be statistically different by the Kruskal–Wallis test (*p* ≤ 0.05), but they could not be resolved by pairwise comparisons with the Wilcoxon test. ^#^ Since ‘other’ and ‘unclassified’ are categories defined as not belonging to a known or specific subgroup of the same taxonomic level, statistical testing was not performed due to the heterogeneity of these undefined groups. ‘Other Phyla’ consisted of Armatimonadetes, Elusimicrobia, Fibrobacteres, Lentisphaerae, Spirochaetes, SR1, Synergistetes, Tenericutes, TM7 and Verrucomicrobia.

**Table 2:** Means of alpha diversity indices from the rumen of lambs that were fed either a forage-based diet or a whole corn-based diet. Forage (D0): *n* = 4; Corn (D56): *n* = 7; Corn (D63): *n*= 8.

Index	Forage (D0)	Corn (D56)	Corn (D63)
observed OTUs	1809.3 ^a^ ± 47.9	439.9 ^b^ ± 15.1	431.5 ^b^ ± 16.4
chao	3854.6 ^a^ ± 201.1	999.8 ^b^ ± 46.1	943.2 ^b^ ± 30.9
ace	5671.8 ^a^ ± 367.5	1439.8 ^b^ ± 66.8	1546.5 ^b^ ± 117.8
Shannon	6.42 ^a^ ± 0.08	3.56 ^b^ ± 0.12	3.36 ^b^ ± 0.12
Simpson	0.009 ^a^ ± 0.002	0.089 ^b^ ± 0.014	0.116 ^b^ ± 0.016
coverage	83.5 ^a^ ± 0.6%	96.1 ^b^ ± 0.1%	96.1 ^b^ ± 0.1%

^a,b^ Values with different superscripts were found to be significantly different (*p* ≤ 0.05) by Tukey’s range test for multiple comparisons.

**Table 3:** Median relative abundance (%) of main bacterial OTUs from the rumen of lambs that were fed either a forage-based diet or a whole corn-based diet. Forage (D0): *n* = 4; Corn (D56): *n* = 7; Corn (D63): *n *= 8.

OTUs	D0	D56	D63	Closest Valid Taxon (% Identity)
***Bacillota***				
Oa-00002	0.12 ^a^	0.26 ^ab^	3.04 ^b^	*Ruminococcoides bili* (94.8%)
Oa-00003	0.08 ^a^	9.41 ^b^	1.97 ^b^	*Dialister succinatiphilus* (94.4%)
Oa-00004	0.08 ^a^	1.52 ^b^	3.57 ^b^	*Chordicoccus furentiruminis* (99.8%)
Oa-00007 ^$^	0.08	2.43	0.15	*Coprococcus eutactus* (91.7%)
Oa-00013	0.02 ^a^	0.77 ^ab^	1.44 ^b^	*Ligilactobacillus ruminis* (94.4%)
Oa-00015	0.03 ^a^	1.04 ^b^	1.67 ^b^	*Limosilactobacillus mucosae* (100%)
Oa-00016	0.03 ^a^	0.63 ^b^	1.03 ^b^	*Massiliimalia timonensis* (85.3%)
Oa-00018	0.04 ^a^	1.13 ^b^	1.15 ^b^	*Eubacterium oxidoreducens* (88.6%)
Oa-00049	1.77 ^a^	<0.01 ^b^	0.01 ^b^	*Saccharofermentans acetigenes* (87.9%)
Oa-00056	1.20 ^a^	<0.01 ^b^	<0.01 ^b^	*Aristaeella lactis* (95.4%)
Oa-00062	1.00 ^a^	<0.01 ^b^	<0.01 ^b^	*Aristaeella lactis* (97.0%)
***Bacteroidota***				
Oa-00005	0.08 ^a^	5.97 ^b^	0.65 ^c^	*Xylanibacter ruminicola* (90.7%)
Oa-00011	0.03 ^a^	2.63 ^b^	1.34 ^b^	*Hallella mizrahii* (99.6%)
Oa-00012	0.06 ^a^	2.25 ^b^	0.60 ^b^	*Xylanibacter ruminicola* (90.9%)
Oa-00019	0.05 ^a^	1.36 ^b^	0.60 ^a^	*Xylanibacter ruminicola* (91.1%)
Oa-00044	1.43 ^a^	<0.01 ^b^	<0.01 ^b^	*Odoribacter splanchnicus* (81.6%)
Oa-00053	1.17 ^a^	0.01 ^b^	0.01 ^b^	*Xylanibacter ruminicola* (91.7%)
***Pseudomonadota***				
Oa-00009 ^$^	0.05	1.73	0.08	*Succinivibrio dextrinosolvens* (97.0%)
**Unclassified**				
Oa-00024	4.38 ^a^	0.02 ^b^	0.03 ^b^	*Pseudoselenomonas ruminantium* (84.5%)

^$^ Values were found to be statistically different by the Kruskal–Wallis test (*p* ≤ 0.05), but they could not be resolved by pairwise comparisons with the Wilcoxon test. ^a,b,c^ Values with different superscripts were found to be significantly different by the Wilcoxon test (*p* ≤ 0.05).

## Data Availability

Raw sequence data are available from the NCBI Sequence Read Archive under project PRJNA1499784.

## Supplementary Materials

The following supporting information can be downloaded at: https://www.mdpi.com/article/10.3390/microorganisms14102134/s1, Supplementary File S1: Perl Scripts for Bioinformatics; Supplementary File S2: OTU Abundance Table; Supplementary File S3: Metagenomics and Gene Annotation Analyses (Oa-00002/Oscillospiraceae, Lachnospiraceae, Prevotellaceae); Supplementary File S4: Oa-00002: Amino Acid and ATP Synthesis Pathways; Supplementary File S5: Gene Annotation Analyses (Oa-00004, Oa-00009, Oa-00011, Oa-00015); Figure S1: Oa-00002: Amino Acid Synthesis Pathway-1; Figure S2: Oa-00002: Amino Acid Synthesis Pathway-2; Figure S3: Oa-00002: Amino Acid Synthesis Pathway-3; Figure S4: Oa-00002: Amino Acid Synthesis Pathway-4; Figure S5: Oa-00002: ATP Synthesis by Substrate-Level Phosphorylation.
