# Supplementary material for: Enrichment of Candidate Formate and Acetaldehyde-Producing Ruminal Bacteria in Response to Corn-Based Diets
Source: Microorganisms. 2026 Sep 22;14(10):2134. doi: 10.3390/microorganisms14102134 (PMC13707844; doi:10.3390/microorganisms14102134)
Supplement: Supplementary file 1 [file microorganisms-14-02134-s001.zip › Anema et al_Supplementary File S1_Perl Scripts.pdf]

A. 16S: quality screening

A.1 MiSeq_FOR_REV_screen_trim_individual sample
A.2 MiSeq_length_selection_multiple_files
A.3 MiSeq_removing_sequences_with_N_multiple_files
A.4 MiSeq_cutoff_screen_average_quality_score_per_read_multiple_files
A.5 MiSeq_read_name_change_multiple_inputs_to_single_output
A.6 Fasta_to_phylip_conversion
A.7 MiSeq_removing_barcodes_from_sequences

B. 16S: V1-V3 alignment

B1.16S_adjusting_5prime_end_based_on_position
B2.16S_5prime_end_sequence_check
B3.16S_C2_region_position_adjustment
B4.16S_C2_region_alignment_check
B5.16S_C3_begin_region_position_adjustment
B6.16S_C3_begin_region_alignment_check
B7.16S_C3_end_region_position_adjustment
B8.16S_C4_begin_region_position_adjustment
B9.16S_C4_trimming_ends
B10.16S_alignment_length_adjustment

C. 16S: clustering and OTU quality screening

C.1 16S_OTU_clustering_tool_using_5prime_to_C4_sorted_phylip
C.2 16S_OTU_List_transform_clustering_list_into_format1_and_format2
C.3 16S_OTU_blast_16S_sorting_sequence_identities_to_alignments
C.4 16S_OTU_blast_processing_results_for_sorting_BAD_and_other_reads
C.5 16S_OTU_Identities_file_retrieving_lines_from_accnos_file_name_adjustment
C.6 16S_OTU_read_count_tally_analysis_for_5P_to_C4_sorting_list_file_format2
C.7 16S_OTU_Count_file_relative_abundance_from_itemized_count_output

D. Metagenomics: contig building

D.1 Contig_sequential_building_from_kmer_input
D.2 Contig_consensus_from_Upstream_building_outputs
D.3 Contig_consensus_from_Downstream_building_outputs
D.4 Contig_assembly_consensus_from_building_outputs_with_N_check
D.5 Contig_consolidating_assembled_consensus
D.6 Contig_merging_from_upstream_downstream

#A.1_MiSeq_FOR_REV_screen_trim_individual sample

```
$INPUT_FASTA_FILE = "Input file name.fasta" ;

@ARRAY_INPUT_FILE = split('\.',$INPUT_FASTA_FILE) ;
$NAME_OF_FILE = $ARRAY_INPUT_FILE[0] ;

#$FORWARD_PRIMER = "GTTTGATC.TGGCTCAG" ; # "AG.GTTTGATC.TGGCTCAG"
#$BARCODE_FORWARD_PRIMER = "AG.GTTTGATC.TGGCTCAG" ; #27F
$BARCODE_FORWARD_PRIMER = "AG.GTTTGATC.TGGCTCAG" ; #27F for SDSU facility
#$BARCODE_FORWARD_PRIMER = "GTGCCAGC.GCCGCGGTAA" ; #515F
$REVERSE_PRIMER = "CAGC.GCCGC.GTAA" ; #519R
#$REVERSE_PRIMER = "TACGG.AGGCAGCAG" ; # 357R BelanchE 2016 :      CTGCTGCCTYCCGTA
#$REVERSE_PRIMER = "ATTAGA.ACCC..GTAGTCC" ; #806F
$LENGTH_TO_SEARCH = 100 ;
$OUTPUT = ""  ;
print "Screening for forward and reverse primer sequences\n" ;
$COUNT = 0 ;
open (FASTA, "$INPUT_FASTA_FILE") or die "Can't open $file for reading" ;
while (<FASTA>)       {
       my $LINE = $_; chomp ($LINE);
       if ($LINE =~ />/)      {
              $COUNT = $COUNT + 1 ;  my $COUNT_THOUSAND = $COUNT/20000 ;
              my $COUNT_INTEGER = int($COUNT_THOUSAND) ; my $INTEGER_CHECK = $COUNT_THOUSAND -
$COUNT_INTEGER;
              if ($INTEGER_CHECK == 0) {print "Screening sequence $COUNT\n";      }

              my $NAME = $LINE ;
              my $SEQUENCE = <FASTA> ; chomp ($SEQUENCE) ;
              my $LENGTH_SEQ = length($SEQUENCE) ;

              my $UPSTREAM_REGION = substr($SEQUENCE,0,$LENGTH_TO_SEARCH);
              my $UPSTREAM_FOUND = 0 ;
              if ($UPSTREAM_REGION =~ /$BARCODE_FORWARD_PRIMER/) {$UPSTREAM_FOUND = 2 ;}

              my $DOWNSTREAM_START = $LENGTH_SEQ - $LENGTH_TO_SEARCH ;
              my $DOWNSTREAM_REGION = substr($SEQUENCE,$DOWNSTREAM_START,$LENGTH_TO_SEARCH);
              my $DOWNSTREAM_FOUND = 0 ;
              if ($DOWNSTREAM_REGION =~ /$REVERSE_PRIMER/) {$DOWNSTREAM_FOUND = 2 ;}

              if ($UPSTREAM_FOUND > 1 and $DOWNSTREAM_FOUND > 1)   { #trimming sequence at rev
primer
                     my $THREE_PRIME_FOUND = 0 ; my $THREE_PRIME_BOUNDARY = 0 ;
                     my $LENGTH_TO_SEARCH = length($REVERSE_PRIMER) ;
                     my $DOWNSTREAM_SCREEN_START = $LENGTH_SEQ - $LENGTH_TO_SEARCH ;
                     while ($THREE_PRIME_FOUND < 1)       {
                            my $DOWNSTREAM_SEARCH = substr($SEQUENCE, $DOWNSTREAM_SCREEN_START,
$LENGTH_TO_SEARCH) ;
                            if ($DOWNSTREAM_SEARCH !~ /$REVERSE_PRIMER/) {
                                   $DOWNSTREAM_SCREEN_START = $DOWNSTREAM_SCREEN_START - 1 ;

                     }
                            if ($DOWNSTREAM_SEARCH =~ /$REVERSE_PRIMER/) {
                                   $THREE_PRIME_FOUND =  2 ;
                                   $THREE_PRIME_BOUNDARY = $DOWNSTREAM_SCREEN_START +
$LENGTH_TO_SEARCH ;

                     }
                                                                              }
                     my $TRIMMED_SEQUENCE = substr($SEQUENCE,0,$THREE_PRIME_BOUNDARY) ;
                     $OUTPUT = $OUTPUT.="$NAME\n$TRIMMED_SEQUENCE\n" ;

              }
                                          }
                                   }
close (FASTA) ;
open (OUTPUT, ">OUTPUT_$NAME_OF_FILE\_FOR_REV_trim.fasta") or die "Can't open $file for reading"
;
print OUTPUT "$OUTPUT" ;
close (OUTPUT);
```

A.2_MiSeq_length_selection_multiple_files

```
$MASTER_FILE = "Master file input name.txt" ;

open (MASTER, "$MASTER_FILE") or die "Can't open $file for reading" ;
	my $FILE_COUNT = 0 ;
	while (<MASTER>)	{
		$FILE_COUNT = $FILE_COUNT + 1 ;
		print "Processing set number $FILE_COUNT\n" ;
		my $MASTER_FILE_LINE = $_ ;   chomp ($MASTER_FILE_LINE);
		my @ARRAY_MASTER_FILE_LINE = split('\t',$MASTER_FILE_LINE) ;
		my $INPUT_FASTA_FILE = $ARRAY_MASTER_FILE_LINE[0] ;

		my @ARRAY_INPUT_FASTA_NAME = split('\.',$INPUT_FASTA_FILE) ;
		my $INPUT_FASTA_NAME_NO_EXTENSION = $ARRAY_INPUT_FASTA_NAME[0] ;

		my $OUTPUT = "" ;
		print"Setting up hash\n" ;
		my %HASH_LENGTH ;
		my $MAX_LENGTH = 600 ;
		my $MIN_LENGTH = 400 ;

		open (FASTA, "$INPUT_FASTA_FILE") or die "Can't open $file for reading" ;
		$READ_COUNT = 0 ; $LONGEST_READ = 0 ; $OUTPUT = "" ;
		while (<FASTA>) {
			$READ_COUNT = $READ_COUNT + 1 ; my $COUNT_THOUSAND = $READ_COUNT/2000 ;
			my $COUNT_INTEGER = int($COUNT_THOUSAND) ; my $INTEGER_CHECK =
$COUNT_THOUSAND - $COUNT_INTEGER;
			if ($INTEGER_CHECK == 0) {print "Processing sequence $READ_COUNT\n";
	}

			my $LINE = $_ ; chomp ($LINE) ;
			if ($LINE =~ />/)	{
				my $READ_NAME = $LINE ;
				my $SEQ = <FASTA>;  chomp($SEQ) ;
				my $LENGTH_SEQ = length($SEQ) ;

				if ($LENGTH_SEQ > $MIN_LENGTH and $LENGTH_SEQ < $MAX_LENGTH)
	{
					$OUTPUT  = $OUTPUT.="$READ_NAME\n$SEQ\n" ;

							}
								}
						}
		close (FASTA) ;

		print "Preparing output\n" ;

		open (OUTPUT, ">$INPUT_FASTA_NAME_NO_EXTENSION\_$MIN_LENGTH-
$MAX_LENGTH\_nt.fasta") or die "Can't open $file for reading" ;
		print OUTPUT "$OUTPUT" ;
		close (OUTPUT);

						}
close (MASTER) ;
```

A.3_MiSeq_removing_sequences_with_N_multiple_files

```
$MASTER_FILE = "Master file input name.txt" ;

open (MASTER, "$MASTER_FILE") or die "Can't open $file for reading" ;
	my $FILE_COUNT = 0 ;
	while (<MASTER>)	{
		$FILE_COUNT = $FILE_COUNT + 1 ;
		print "Processing set number $FILE_COUNT\n" ;
		my $MASTER_FILE_LINE = $_ ;   chomp ($MASTER_FILE_LINE);
		my @ARRAY_MASTER_FILE_LINE = split('\t',$MASTER_FILE_LINE) ;
		my $INPUT_FASTA_FILE = $ARRAY_MASTER_FILE_LINE[0] ;

		my @ARRAY_INPUT_FASTA_NAME = split('\.',$INPUT_FASTA_FILE) ;
		my $INPUT_FASTA_NAME_NO_EXTENSION = $ARRAY_INPUT_FASTA_NAME[0] ;

		my $OUTPUT = "" ;

		my $COUNT_LINE = 0;
		open (FILE, "$INPUT_FASTA_FILE") or die "Can't open $file for reading" ;

		while (<FILE>)		{
			$COUNT_LINE = $COUNT_LINE + 1;; my $COUNT_THOUSAND = $COUNT_LINE/5000 ;
			my $COUNT_INTEGER = int($COUNT_THOUSAND) ; my $INTEGER_CHECK =
$COUNT_THOUSAND - $COUNT_INTEGER;
			if ($INTEGER_CHECK == 0) {print "Processing line $COUNT_LINE\n";    }

			my $FILE_LINE = $_; chomp($FILE_LINE);
			if ($FILE_LINE =~ />/) {
				my $READ_NAME = $FILE_LINE ;
				my $READ_SEQ = <FILE> ; chomp($READ_SEQ) ;

				my $NUMBER_KEYWORD_MATCHES = 0 ;
				while ($READ_SEQ =~ m/N/g)    {
					$NUMBER_KEYWORD_MATCHES =  $NUMBER_KEYWORD_MATCHES + 1 ;
												}
				if ($NUMBER_KEYWORD_MATCHES < 1)      {
					$OUTPUT = $OUTPUT.="$READ_NAME\n$READ_SEQ\n" ;

	}
										}
				}
close (FILE);

open (OUTPUT, ">$INPUT_FASTA_NAME_NO_EXTENSION\_N_filtered.fasta ") or die "Can't open $file for
reading" ;
print OUTPUT "$OUTPUT" ;
close (OUTPUT);

						}
close (MASTER) ;
```

A.4_MiSeq_cutoff_screen_average_quality_score_per_read_multiple_files

```
$MASTER_FILE = "Master file input name.txt" ;

$CUTOFF_AVERAGE_QUALITY = 33 ;

open (MASTER, "$MASTER_FILE") or die "Can't open $file for reading" ;
$FILE_COUNT = 0 ;
while (<MASTER>)       {
        $FILE_COUNT = $FILE_COUNT + 1 ;
        print "Processing set number $FILE_COUNT\n" ;
        my $MASTER_FILE_LINE = $_ ;   chomp ($MASTER_FILE_LINE);
        my @ARRAY_MASTER_FILE_LINE = split('\t',$MASTER_FILE_LINE) ;
        my $INPUT_FASTA_FILE = $ARRAY_MASTER_FILE_LINE[0] ;
        my $INPUT_QUAL_FILE = $ARRAY_MASTER_FILE_LINE[1] ;

        my @ARRAY_INPUT_FASTA_NAME = split('\.',$INPUT_FASTA_FILE) ;
        my $INPUT_FASTA_NAME_NO_EXTENSION = $ARRAY_INPUT_FASTA_NAME[0] ;

        my %HASH_QUAL ; my %HASH_SURVEY_QUAL ;
        my $OUTPUT = "" ;
        my $OUTPUT_SUMMARY = "\t$INPUT_FASTA_NAME_NO_EXTENSION\n" ;
        my $OUTPUT_ERROR = "Inconsistency between fasta and qual for reads:\n" ;

        open (QUAL, "$INPUT_QUAL_FILE") or die "Can't open $file for reading" ;
        my $READ_COUNT = 0 ;
        while (<QUAL>)        {
                my $LINE = $_ ;       chomp ($LINE);
                if ($LINE =~ />/)             {
                        $READ_COUNT = $READ_COUNT + 1 ; my $COUNT_THOUSAND = $READ_COUNT/20000 ;
                        my $COUNT_INTEGER = int($COUNT_THOUSAND) ; my $INTEGER_CHECK =
$COUNT_THOUSAND - $COUNT_INTEGER;
                        if ($INTEGER_CHECK == 0) {print "Loading quality for $READ_COUNT\n";
        }

                        my @ARRAY_LINE = split(' ',$LINE) ;
                        my $NAME = $ARRAY_LINE[0] ;
                        my $QUAL_DATA_LINE = <QUAL> ; chomp ($QUAL_DATA_LINE) ;
                        $HASH_QUAL{$NAME} = $QUAL_DATA_LINE ;
                                                                      }
                                      }
        close (QUAL);

        open (FASTA, "$INPUT_FASTA_FILE") or die "Can't open $file for reading" ;
        my $READ_COUNT_1 = 0 ;
        print "Screening fasta for quality\n" ;
        while (<FASTA>)       {
                my $LINE = $_ ;       chomp ($LINE);
                if ($LINE =~ />/)     {
                        $READ_COUNT_1 = $READ_COUNT_1 + 1 ; my $COUNT_THOUSAND =
$READ_COUNT_1/20000 ;
                        my $COUNT_INTEGER = int($COUNT_THOUSAND) ; my $INTEGER_CHECK =
$COUNT_THOUSAND - $COUNT_INTEGER;
                        if ($INTEGER_CHECK == 0) {print "Checking read $READ_COUNT_1 for
quality\n";    }

                        my @ARRAY_LINE = split(' ',$LINE) ;
                        my $NAME = $ARRAY_LINE[0] ;
                        my $SEQ = <FASTA> ; chomp($SEQ) ;
                        my $LENGTH_SEQ = length($SEQ) ;

                        my $QUALITY_DATA = $HASH_QUAL{$NAME} ;
                        my @ARRAY_QUALITY_DATA = split(' ',$QUALITY_DATA);
                        my $NUMBER_QUAL_DATA_ENTRIES = scalar(@ARRAY_QUALITY_DATA);
                        if ($NUMBER_QUAL_DATA_ENTRIES < $LENGTH_SEQ)        { $OUTPUT_ERROR =
$OUTPUT_ERROR.="$NAME," ; }

                        my $QUAL_COUNT = 0 ; my $LOW_QUAL_NT_COUNT = 0 ;
                        my $QUAL_CUMULATIVE = 0  ;
                        while ($QUAL_COUNT < $LENGTH_SEQ) {
                               my $QUAL_SCORE = $ARRAY_QUALITY_DATA[$QUAL_COUNT] ;
```

```
                              $QUAL_CUMULATIVE = $QUAL_CUMULATIVE + $QUAL_SCORE ;
                              $QUAL_COUNT = $QUAL_COUNT + 1 ;

                                                                                  }
                       my $AVERAGE_QUALITY_SCORE = $QUAL_CUMULATIVE / $NUMBER_QUAL_DATA_ENTRIES ;
                       if ($AVERAGE_QUALITY_SCORE > $CUTOFF_AVERAGE_QUALITY)       {
                              $OUTPUT = $OUTPUT.="$NAME\n$SEQ\n" ;

                              }
#                      $OUTPUT = $OUTPUT.="$NAME\t$AVERAGE_QUALITY_SCORE\n" ;

#                      my $QUAL_BRACKET = int($AVERAGE_QUALITY_SCORE) ;
#                      my $UPDATE_HASH_INFO = $HASH_SURVEY_QUAL{$QUAL_BRACKET} ;
#                      $UPDATE_HASH_INFO = $UPDATE_HASH_INFO.="$NAME," ;
#                      $HASH_SURVEY_QUAL{$QUAL_BRACKET} = $UPDATE_HASH_INFO ;

                                                    }
                                             }
       close (FASTA) ;
#      print "Preparing summary\n" ;
#      my $HIGHEST_QUALITY = 41 ;
#      my $QUALITY_RANGE_SURVEY = $HIGHEST_QUALITY ;
#      while ($QUALITY_RANGE_SURVEY  > 0)    {
#             my $INFO_TO_TRANSFER = $HASH_SURVEY_QUAL{$QUALITY_RANGE_SURVEY} ;
#             my @ARRAY_INFO_TO_TRANSFER = split(',',$INFO_TO_TRANSFER ) ;
#             my $NUMBER_NAMES = scalar(@ARRAY_INFO_TO_TRANSFER) ;
#             $OUTPUT_SUMMARY = $OUTPUT_SUMMARY."Reads in quality bracket
$QUALITY_RANGE_SURVEY\:\t$NUMBER_NAMES\n" ;
#             $QUALITY_RANGE_SURVEY = $QUALITY_RANGE_SURVEY - 1 ;
#                                                                   }

       open (OUTPUT, ">OUTPUT_Q$CUTOFF_AVERAGE_QUALITY $INPUT_FASTA_NAME_NO_EXTENSION\.fasta")
or die "Can't open $file for reading" ;
       print OUTPUT "$OUTPUT_ERROR\n\n" ;
#      print OUTPUT "$OUTPUT_SUMMARY\n\n" ;
       print OUTPUT "$OUTPUT" ;
       close (OUTPUT);
                              }
close (MASTER) ;
```

A.5_MiSeq_read_name_change_multiple_inputs_to_single_output

```
$MASTER_FILE = "Master_file_Input_name.txt" ;

$OUTPUT = "" ; $OUTPUT_SUMMARY = "" ;
open (MASTER, "$MASTER_FILE") or die "Can't open $file for reading" ;
$FILE_COUNT = 0 ;
while (<MASTER>)      {
       $FILE_COUNT = $FILE_COUNT + 1 ;
       print "Processing set number $FILE_COUNT\n" ;
       my $MASTER_FILE_LINE = $_ ;   chomp ($MASTER_FILE_LINE);
       my @ARRAY_MASTER_FILE_LINE = split('\t',$MASTER_FILE_LINE) ;
       my $INPUT_FASTA_FILE = $ARRAY_MASTER_FILE_LINE[0] ;
       my $READ_DESIGNATOR = $ARRAY_MASTER_FILE_LINE[1] ;

       print "Counting number of reads\n" ;
       my $COUNT = 0 ; my $NUMBER_OF_READS = 0 ;
       open (FILE, "$INPUT_FASTA_FILE") or die "Can't open $file for reading" ;
       while (<FILE>)        {
       my $LINE = $_; chomp ($LINE);
       if ($LINE =~ />/)      {
               $COUNT = $COUNT + 1 ;  my $COUNT_THOUSAND = $COUNT/20000 ;
               my $COUNT_INTEGER = int($COUNT_THOUSAND) ; my $INTEGER_CHECK = $COUNT_THOUSAND -
$COUNT_INTEGER;
               if ($INTEGER_CHECK == 0) {print "Counting reads, sequence $COUNT\n";       }

               my $SEQUENCE = <FILE> ; chomp ($SEQUENCE) ;
               $NUMBER_OF_READS = $NUMBER_OF_READS + 1;
                                               }
                                       }
       close (FILE) ;

       print "Renaming reads\n" ;
       my $RENAME_COUNT = 0 ; my $RENAMED_READS = "" ;
       my $COUNT = 0 ;
       open (FILE_2, "$INPUT_FASTA_FILE") or die "Can't open $file for reading" ;
       while (<FILE_2>)              {
               my $LINE = $_; chomp ($LINE);
               if ($LINE =~ />/)      {
                       $COUNT = $COUNT + 1 ;  my $COUNT_THOUSAND = $COUNT/20000 ;
                       my $COUNT_INTEGER = int($COUNT_THOUSAND) ; my $INTEGER_CHECK =
$COUNT_THOUSAND - $COUNT_INTEGER;
                       if ($INTEGER_CHECK == 0) {print "Renaming reads, sequence $COUNT\n";
       }

                       my $NAME = $LINE ; my $SEQUENCE = <FILE_2> ; chomp ($SEQUENCE) ;

               my $PADDING_NUMBER = 0 ;
               if ($NUMBER_OF_READS < 100) { $PADDING_NUMBER = 2 ;}
               if ($NUMBER_OF_READS >= 100 && $NUMBER_OF_READS < 1000) {$PADDING_NUMBER = 3 ; }
               if ($NUMBER_OF_READS >= 1000 && $NUMBER_OF_READS < 10000) {$PADDING_NUMBER = 4 ; }
               if ($NUMBER_OF_READS >= 10000 && $NUMBER_OF_READS < 100000) {$PADDING_NUMBER = 5 ;
}
               if ($NUMBER_OF_READS >= 100000 && $NUMBER_OF_READS < 1000000) {$PADDING_NUMBER = 6
; }
               if ($NUMBER_OF_READS >= 1000000 && $NUMBER_OF_READS < 10000000) {$PADDING_NUMBER =
7 ; }
               if ($NUMBER_OF_READS >= 10000000 && $NUMBER_OF_READS < 100000000) {$PADDING_NUMBER
= 8 ; }

               $RENAME_COUNT = $RENAME_COUNT + 1;
               my $PADDED_COUNT = sprintf("%0$PADDING_NUMBER.f", $RENAME_COUNT) ;
               my $NEW_NAME = ">$READ_DESIGNATOR-$PADDED_COUNT" ;
               $RENAMED_READS = $RENAMED_READS.="$NEW_NAME\n$SEQUENCE\n";

                                               }
                                               }
       close (FILE_2);
       $OUTPUT = $OUTPUT.="$RENAMED_READS" ;
       $OUTPUT_SUMMARY = $OUTPUT_SUMMARY.="$INPUT_FASTA_FILE\treads
renamed:\t$NUMBER_OF_READS\n" ;
```

```
                                            }
close (MASTER) ;

       print "Preparing output\n" ;
       open (OUTPUT, ">OUTPUT_renamed_reads_from $MASTER_FILE.fasta") or die "Can't open $file
for reading" ;
       print OUTPUT "$OUTPUT" ;
       close (OUTPUT);

       open (OUTPUT_SUMMARY, ">OUTPUT_Summary_renamed_reads_from $MASTER_FILE.txt") or die
"Can't open $file for reading" ;
       print OUTPUT_SUMMARY "$OUTPUT_SUMMARY" ;
       close (OUTPUT_SUMMARY);
```

A.6 Fasta_to_phylip_conversion

```
$INPUT_FILE_NAME = "Input file name.fasta" ;

@ARRAY_FILE_NAME = split('\.',$INPUT_FILE_NAME) ;
$FILE_NAME_NO_EXTENSION = $ARRAY_FILE_NAME[0] ;

open (FASTA, "$INPUT_FILE_NAME") or die "Can't open $file for reading" ;

$READ_COUNT = 0 ; $OUTPUT = "" ;
while (<FASTA>)       {
       my $LINE = $_ ;        chomp ($LINE);
       if ($LINE =~ />/)      {
               $READ_COUNT = $READ_COUNT + 1 ; my $COUNT_THOUSAND = $READ_COUNT/10000 ;
               my $COUNT_INTEGER = int($COUNT_THOUSAND) ; my $INTEGER_CHECK = $COUNT_THOUSAND -
$COUNT_INTEGER;
               if ($INTEGER_CHECK == 0) {print "Checking sequence $READ_COUNT\n";  }

               if ($LINE =~ />/)      {
                       my $NAME = $LINE ;
                       my $SEQUENCE = <FASTA> ; chomp ($SEQUENCE) ;
                       $OUTPUT = $OUTPUT.="$NAME\t$SEQUENCE\n";
                                                       }
                                               }
                                       }
close (FASTA);
open (OUTPUT, ">OUTPUT_converted_to_phylip_$FILE_NAME_NO_EXTENSION.phylip") or die "Can't open
$file for reading" ;
print OUTPUT "$OUTPUT";
close (OUTPUT);
```

A.7_MiSeq_removing_barcodes_from_sequences

```
$INPUT_FILE_NAME = " Input_file_name.phylip" ;
open (FILE, "$INPUT_FILE_NAME") or die "Can't open $file for reading" ;

@ARRAY_FILE_NAME = split('\.',$INPUT_FILE_NAME) ;
$FILE_NAME_NO_EXTENSION = $ARRAY_FILE_NAME[0] ;

$LENGTH_OF_BARCODE = 8 ;       #####################################

$READ_COUNT = 0 ;
$OUTPUT = "";

while (<FILE>)         {
       $READ_COUNT = $READ_COUNT + 1 ; my $COUNT_THOUSAND = $READ_COUNT/5000 ;
       my $COUNT_INTEGER = int($COUNT_THOUSAND) ; my $INTEGER_CHECK = $COUNT_THOUSAND -
$COUNT_INTEGER;
       if ($INTEGER_CHECK == 0) {print "Checking sequence $READ_COUNT\n";  }

       my $LINE = $_;         chomp ($LINE);

       my @ARRAY_LINE = split('\t', $LINE) ;
       my $NAME = $ARRAY_LINE[0] ; my $SEQ = $ARRAY_LINE[1] ;
       my $SEQ_LENGTH = length($SEQ) ;
       my $SEQ_LENGTH_NO_BARCODE = $SEQ_LENGTH - $LENGTH_OF_BARCODE ;
       my $NO_BARCODE_SEQ = substr($SEQ, $LENGTH_OF_BARCODE, $SEQ_LENGTH_NO_BARCODE);
       $OUTPUT = $OUTPUT.="$NAME\t$NO_BARCODE_SEQ\n" ;

                              }


close (FILE);

open (OUTPUT, ">OUTPUT_$FILE_NAME_NO_EXTENSION\_Barcodes_Removed.phylip") or die "Can't open
$file for reading" ;
print OUTPUT "$OUTPUT" ;
close (OUTPUT);
```

B1.16S_adjusting_5prime_end_based_on_position

```
$INPUT_FILE_NAME = "Input_file_name.phylip;
open (PHYLIP, "$INPUT_FILE_NAME") or die "Can't open $file for reading" ;

$TEMPLATE = "AGAGTTTGATCATGGCTCAGGAT-GAA-CGCTAG-CTA-CAGG-CTTAA-CAC-ATGC-AAGTCGAGCGGTA-" ; #for datasets
#$TEMPLATE = "TGGCTCAGGAT-GAA-CGCTAG-CTA-CAGG-CTTAA-CAC-ATGC-AAGTCGAGCGGTA-" ; #for 16S refseqs
@ARRAY_NT_TEMPLATE = split('',$TEMPLATE) ; $LENGTH_ARRAY_TEMPLATE = scalar(@ARRAY_NT_TEMPLATE) ;
$LIST_DASH_POSITIONS = "" ; $COUNT_SCREEN_TEMPLATE = 0 ;
print "Determining position of dashes in template\n" ;
while ($COUNT_SCREEN_TEMPLATE < $LENGTH_ARRAY_TEMPLATE) {
        my $NT = $ARRAY_NT_TEMPLATE[$COUNT_SCREEN_TEMPLATE] ;
        if ($NT =~ /-/) {$LIST_DASH_POSITIONS = $LIST_DASH_POSITIONS.="$COUNT_SCREEN_TEMPLATE\t" ;}
        $COUNT_SCREEN_TEMPLATE = $COUNT_SCREEN_TEMPLATE + 1 ;

        }
print "Determining length of sequence blocks in template\n" ;
@ARRAY_BLOCK_TEMPLATE = split('-',$TEMPLATE) ;
$NUMBER_ARRAY_BLOCK = scalar(@ARRAY_BLOCK_TEMPLATE) ;
$LIST_BLOCK_SIZES = "" ; $COUNT_SCREEN_TEMPLATE = 0 ;

while ($COUNT_SCREEN_TEMPLATE < $NUMBER_ARRAY_BLOCK)        {
        my $NT_BLOCK = @ARRAY_BLOCK_TEMPLATE[$COUNT_SCREEN_TEMPLATE] ;
        my $LENGTH_NT_BLOCK = length($NT_BLOCK) ;
        $LIST_BLOCK_SIZES = $LIST_BLOCK_SIZES.="$LENGTH_NT_BLOCK\t" ;
        $COUNT_SCREEN_TEMPLATE = $COUNT_SCREEN_TEMPLATE + 1;

        }


@ARRAY_DASH_POSITIONS = split('\t', $LIST_DASH_POSITIONS) ;
$NUMBER_OF_DASHES = scalar(@ARRAY_DASH_POSITIONS) ;
@ARRAY_BLOCK_SIZES = split('\t', $LIST_BLOCK_SIZES) ;

$READ_COUNT = 0 ; $PRE_OUTPUT = "" ;

while (<PHYLIP>) {
        $READ_COUNT = $READ_COUNT + 1 ; my $COUNT_THOUSAND = $READ_COUNT/2000 ;
        my $COUNT_INTEGER = int($COUNT_THOUSAND) ; my $INTEGER_CHECK = $COUNT_THOUSAND - $COUNT_INTEGER;
        if ($INTEGER_CHECK == 0) {print "Checking sequence $READ_COUNT\n";  }

        my $LINE = $_ ;        chomp ($LINE); my @ARRAY_LINE = split('\t',$LINE) ;
        my $NAME = $ARRAY_LINE[0] ;   my $SEQ = $ARRAY_LINE[1] ; my $LENGTH_SEQ = length($SEQ) ;

        my $ADJUSTED_SEQ = "" ;
        my $DASH_COUNT = 0 ; my $START = 0; my $DASH_POS = 0 ; my $LENGTH_BLOCK = 0 ;
        while ($DASH_COUNT < $NUMBER_OF_DASHES) {
                $DASH_POS = $ARRAY_DASH_POSITIONS[$DASH_COUNT] ;
                $LENGTH_BLOCK = $ARRAY_BLOCK_SIZES[$DASH_COUNT] ;
                my $BLOCK_SEQ = substr($SEQ, $START, $LENGTH_BLOCK) ;
                $ADJUSTED_SEQ = $ADJUSTED_SEQ.="$BLOCK_SEQ-" ;
                $START = $START + $LENGTH_BLOCK ;
                $DASH_COUNT = $DASH_COUNT + 1;
                                                                                }
        my $LENGTH_DOWNSTREAM = $LENGTH_SEQ - $START ;
        my $DOWNSTREAM_SEQ = substr($SEQ, $START, $LENGTH_DOWNSTREAM) ;

        $PRE_OUTPUT = $PRE_OUTPUT.="$NAME\t$ADJUSTED_SEQ" ;
        $PRE_OUTPUT = $PRE_OUTPUT.="$DOWNSTREAM_SEQ\n" ;

                                }
close (PHYLIP);


open (OUTPUT, ">OUTPUT_5prime_adjusted_$INPUT_FILE_NAME") or die "Can't open $file for reading" ;
print OUTPUT "$PRE_OUTPUT" ;
close (OUTPUT);
```

B2.16S_5prime_end_sequence_check

```
$INPUT_FILE_NAME = "Input_file_name.phylip " ;
open (PHYLIP, "$INPUT_FILE_NAME") or die "Can't open $file for reading" ;

#$CONSENSUS = "AG.GTTTGATC.TGGCTCAGGAT-GAA-CGCTAG-CGG-CAGG-CTTAA-CAC-ATGC-AA" ;    #4178
#$CONSENSUS = "AG.GTTTGATC.TGGCTCAG...-GAA-......-...-....-.....-...-....-AA" ;    #91354
$CONSENSUS = "AG.GTTTGATC.TGGCTCAG...-...-......-...-....-.....-...-....-A." ; #91354 + 4584
$READ_COUNT = 0 ;
$OUTPUT_GOOD = "" ; $OUTPUT__TO_CHECK = "" ;
$GOOD_COUNT = 0 ; $TO_CHECK_COUNT = 0 ;

while (<PHYLIP>) {
        $READ_COUNT = $READ_COUNT + 1 ; my $COUNT_THOUSAND = $READ_COUNT/2000 ;
        my $COUNT_INTEGER = int($COUNT_THOUSAND) ; my $INTEGER_CHECK = $COUNT_THOUSAND -
$COUNT_INTEGER;
        if ($INTEGER_CHECK == 0) {print "Checking sequence $READ_COUNT\n";  }

        my $LINE = $_ ;        chomp ($LINE); my @ARRAY_LINE = split('\t',$LINE) ;
        my $NAME = $ARRAY_LINE[0] ;   my $SEQ = $ARRAY_LINE[1] ; my $LENGTH_SEQ = length($SEQ) ;

        if ($SEQ =~ /$CONSENSUS/)     {
                $OUTPUT_GOOD = $OUTPUT_GOOD.="$NAME\t$SEQ\n" ;
                $GOOD_COUNT = $GOOD_COUNT + 1 ;
                                                        }
        if ($SEQ !~ /$CONSENSUS/)     {
                $OUTPUT__TO_CHECK = $OUTPUT__TO_CHECK.="$NAME\t$SEQ\n" ;
                $TO_CHECK_COUNT = $TO_CHECK_COUNT + 1 ;
                                                        }

                                }
close (PHYLIP);


open (OUTPUT, ">OUTPUT_5prime_checked_sequence_$INPUT_FILE_NAME") or die "Can't open $file for
reading" ;
print OUTPUT "good sequences have consensus $CONSENSUS-:$GOOD_COUNT\n$OUTPUT_GOOD\n" ;
close (OUTPUT);

open (OUTPUT_2, ">OUTPUT_5prime_recheck_sequence_$INPUT_FILE_NAME") or die "Can't open $file for
reading" ;
print OUTPUT_2 "sequences to check:$TO_CHECK_COUNT\n$OUTPUT__TO_CHECK\n" ;
close (OUTPUT_2);
```

B3.16S_C2_region_position_adjustment

```
$INPUT_FILE_NAME = "Input_file_name.phylip" ;
open (PHYLIP, "$INPUT_FILE_NAME") or die "Can't open $file for reading" ;


$V1_TOTAL_LENGTH = 150 ;
$V1_C2_TOTAL_LENGTH = 212 ;

$CONSENSUS_1A = "TAATAC...AT" ;
$CONSENSUS_1B = "TAACAC....T" ;
$CONSENSUS_1C = "TAATAC....C" ;
$CONSENSUS_1D = "T.ATAC...AT" ;
$CONSENSUS_1E = "TAA..CC..GC" ;
$CONSENSUS_1F = "TGATAC...GT" ;
$CONSENSUS_1G = "TAA.AC...AT" ;
#............CATTTAAGAACGAAT # V1 sequences that need to be excluded from consensus
#...............GTAATACGAGCT  # V1 sequences that need to be excluded from consensus
$CONSENSUS_2 =  "TAA..CC..AT" ;
$CONSENSUS_3 =  "TAA..CT..AT" ;
$CONSENSUS_4 =  "TAA..CC..CT" ;
$CONSENSUS_5 =  "TAA..CT..CT" ;
$CONSENSUS_6 =  "TAA..CC..AG" ;
$CONSENSUS_7 =  "TAA..CC..CG" ;
$CONSENSUS_8 =  "TAA..CT..AG" ;
$CONSENSUS_9 =  "TAA..CT..CG" ;
#Lee's consensus sequences
$CONSENSUS_10 = "CAAAACCGAAT" ;
$CONSENSUS_11 = "GAGGA.CC.C." ;
$CONSENSUS_12 = "TAATG.C..G." ;
$CONSENSUS_12A ="TAAT..CG.GT" ;
$CONSENSUS_13 = "T.ATC.C...." ; #this one is probaly too loose
$CONSENSUS_13B= "TAA...C..AT" ;
$CONSENSUS_14 = "A.TCCCAATAT" ;

#$CONSENSUS_LIST = $CONSENSUS_LIST.="";
$CONSENSUS_LIST =
"$CONSENSUS_1A,$CONSENSUS_1B,$CONSENSUS_1C,$CONSENSUS_1D,$CONSENSUS_1E,$CONSENSUS_1F,$CONSENSUS_1
G,";
$CONSENSUS_LIST =
$CONSENSUS_LIST.="$CONSENSUS_2,$CONSENSUS_3,$CONSENSUS_4,$CONSENSUS_5,$CONSENSUS_6,$CONSENSUS_7"
;
$CONSENSUS_LIST =
$CONSENSUS_LIST.=",$CONSENSUS_8,$CONSENSUS_9,$CONSENSUS_10,$CONSENSUS_11,$CONSENSUS_12,$CONSENSUS
_12A" ;
$CONSENSUS_LIST = $CONSENSUS_LIST.=",$CONSENSUS_13,$CONSENSUS_13B,$CONSENSUS_14" ;
@ARRAY_CONSENSUS = split(',',$CONSENSUS_LIST) ;
$NUMBER_CONSENSUS_SEQ = scalar(@ARRAY_CONSENSUS) ;

$OUTPUT_FOUND = "" ; $OUTPUT_NOT_FOUND = "" ; $OUTPUT_SUMMARY = "" ;
$OUTPUT_MULTIPLE_CONSENSUS = "" ;
%HASH_COUNT ; $COUNT_HASH_SET_UP = 0 ;
%HASH_POSITION_BASED_ON_DASHES ; $HIGHEST_NUMBER_DASHES = 0 ;
while ($COUNT_HASH_SET_UP < $NUMBER_CONSENSUS_SEQ)   {
        $HASH_COUNT{$COUNT_HASH_SET_UP} = 0 ;
        $COUNT_HASH_SET_UP = $COUNT_HASH_SET_UP + 1 ;

        }
$READ_COUNT = 0 ;
while (<PHYLIP>)       {
        $READ_COUNT = $READ_COUNT + 1 ; my $COUNT_THOUSAND = $READ_COUNT/2000 ;
        my $COUNT_INTEGER = int($COUNT_THOUSAND) ; my $INTEGER_CHECK = $COUNT_THOUSAND -
$COUNT_INTEGER;
        if ($INTEGER_CHECK == 0) {print "Aligning sequence $READ_COUNT\n";  }
#       print "Checking sequence $READ_COUNT\n";
        my $LINE = $_ ;        chomp ($LINE);

        if ($LINE =~ />/) {
                my @ARRAY_LINE = split('\t',$LINE) ;
                my $NAME = $ARRAY_LINE[0] ;   my $SEQ = $ARRAY_LINE[1] ; my $LENGTH_SEQ =
length($SEQ) ;
```

```
                my @ARRAY_SEQ_BLOCKS = split('-', $SEQ) ;
                my $NUMBER_OF_BLOCKS = scalar(@ARRAY_SEQ_BLOCKS) ;
                my $SECOND_TO_LAST_BLOCK = $NUMBER_OF_BLOCKS - 1 ;
                my $LENGTH_FIVE_PRIME = 0 ;
                my $COUNT_BLOCK = 0 ; my $REBUILDING_SEQUENCE = "" ;
                while ($COUNT_BLOCK < $NUMBER_OF_BLOCKS) {
                        my $BLOCK = $ARRAY_SEQ_BLOCKS[$COUNT_BLOCK] ;
                        $REBUILDING_SEQUENCE = $REBUILDING_SEQUENCE.="$BLOCK-" ;
                        if ($COUNT_BLOCK < $SECOND_TO_LAST_BLOCK)    {
                                $LENGTH_FIVE_PRIME = length($REBUILDING_SEQUENCE) ;

        }
                        $COUNT_BLOCK = $COUNT_BLOCK + 1 ;
                                                                                        }
                my $DOWNSTREAM_REGION = $ARRAY_SEQ_BLOCKS[$COUNT_BLOCK] ;
                my $CONSENSUS_FOUND = 0 ; my $CONSENSUS_FOUND_COPY = 0 ;
                my $ALIGNED_SEQ = "" ; my $NUMBER_OF_DASHES = 0 ;
                my $COUNT_SCREEN = 0 ;
                while ($COUNT_SCREEN < $NUMBER_CONSENSUS_SEQ and $CONSENSUS_FOUND < 1)     {
                        my $CONSENSUS_TO_SCREEN = $ARRAY_CONSENSUS[$COUNT_SCREEN] ;
                        my $LENGTH_CONSENSUS = length($CONSENSUS_TO_SCREEN) ;
                        if ($SEQ =~ /$CONSENSUS_TO_SCREEN/)   {
                                $CONSENSUS_FOUND = 2 ;
                                my $INFO_TO_UPDATE = $HASH_COUNT{$COUNT_SCREEN} ;
                                $INFO_TO_UPDATE = $INFO_TO_UPDATE + 1 ;
                                $HASH_COUNT{$COUNT_SCREEN} = $INFO_TO_UPDATE ;

                                my $FIVE_PRIME_BOUNDARY =  0 ; my $THREE_PRIME_BOUNDARY = 0 ;
                                my $DOWNSTREAM_LENGTH = 0 ;
                                my $THREE_PRIME_FOUND =  0 ;
                                my $LENGTH_TO_SCREEN = $LENGTH_CONSENSUS ;
                                while ($THREE_PRIME_FOUND < 1)       {
                                        my $DOWNSTREAM_SEARCH = substr($SEQ, 0, $LENGTH_TO_SCREEN)
;
                                        if ($DOWNSTREAM_SEARCH !~ /$CONSENSUS_TO_SCREEN/)    {
                                                $LENGTH_TO_SCREEN = $LENGTH_TO_SCREEN + 1 ;

                                }
                                        if ($DOWNSTREAM_SEARCH =~ /$CONSENSUS_TO_SCREEN/)    {
                                                $FIVE_PRIME_BOUNDARY = $LENGTH_TO_SCREEN -
$LENGTH_CONSENSUS;
                                                $THREE_PRIME_BOUNDARY = $LENGTH_TO_SCREEN ;
                                                $DOWNSTREAM_LENGTH = $LENGTH_SEQ -
$THREE_PRIME_BOUNDARY ;
                                                $THREE_PRIME_FOUND =  2 ;

                                        }

                                                                                        }
                                my $FIVE_PRIME_REGION = substr($SEQ, 0, $LENGTH_FIVE_PRIME) ;
#                               my $CONSERVED_REGION = substr($SEQ,$FIVE_PRIME_BOUNDARY,
$LENGTH_CONSENSUS) ;
                                my $LENGTH_V1_C2_NOT_ALIGNED = $THREE_PRIME_BOUNDARY -
$LENGTH_FIVE_PRIME ;
                                my $V1_C2 = substr($SEQ,$LENGTH_FIVE_PRIME,
$LENGTH_V1_C2_NOT_ALIGNED);
                                $NUMBER_OF_DASHES = $V1_C2_TOTAL_LENGTH - $LENGTH_V1_C2_NOT_ALIGNED
;
                                my $DASHES_COUNT = 0 ; my $V1_DASHES = "" ;
                                while ($DASHES_COUNT < $NUMBER_OF_DASHES)    {
                                        $V1_DASHES = $V1_DASHES.="-" ;
                                        $DASHES_COUNT = $DASHES_COUNT + 1 ;

                }
                                my $DOWNSTREAM = substr($SEQ,$THREE_PRIME_BOUNDARY
,$DOWNSTREAM_LENGTH) ;
                                $ALIGNED_SEQ = "$FIVE_PRIME_REGION-$V1_DASHES-$V1_C2--$DOWNSTREAM"
;
                                #$OUTPUT_FOUND = $OUTPUT_FOUND.="$NAME\t$ALIGNED_SEQ\n" ;
```

```
                              my $SEQ_HASH_TRANSFER =
$HASH_POSITION_BASED_ON_DASHES{$NUMBER_OF_DASHES} ;
                              $SEQ_HASH_TRANSFER = $SEQ_HASH_TRANSFER.="$NAME\t$ALIGNED_SEQ\n" ;
                              $HASH_POSITION_BASED_ON_DASHES{$NUMBER_OF_DASHES} =
$SEQ_HASH_TRANSFER ;
                              if ($NUMBER_OF_DASHES > $HIGHEST_NUMBER_DASHES )     {
                                      $HIGHEST_NUMBER_DASHES = $NUMBER_OF_DASHES ;

                              }
#determining if other copies of consensus present downstream
                              my $COUNT_SCREEN_COPY = 0 ;
                              while ($COUNT_SCREEN_COPY < $NUMBER_CONSENSUS_SEQ and
$CONSENSUS_FOUND_COPY < 1)    {
                                      my $CONSENSUS_TO_SCREEN =
$ARRAY_CONSENSUS[$COUNT_SCREEN_COPY] ;
                                      my $LENGTH_CONSENSUS = length($CONSENSUS_TO_SCREEN) ;
                                      if ($DOWNSTREAM  =~ /$CONSENSUS_TO_SCREEN/)  {
$CONSENSUS_FOUND_COPY = 2 ; }
                                      $COUNT_SCREEN_COPY = $COUNT_SCREEN_COPY + 1 ;

                                                                                          }

                                              }
                      $COUNT_SCREEN = $COUNT_SCREEN + 1 ;

                                                      }
              if ($CONSENSUS_FOUND < 1)     {
                      $OUTPUT_NOT_FOUND = $OUTPUT_NOT_FOUND.="$LINE\n" ;
                                                                      }
              if ($CONSENSUS_FOUND_COPY > 1)       {
                      $OUTPUT_MULTIPLE_CONSENSUS = $OUTPUT_MULTIPLE_CONSENSUS.="$LINE\n" ;
                                                                              }
              if ($CONSENSUS_FOUND_COPY < 1)       {

                                                                              }
                                              }
                                      }
close (PHYLIP);

print "Preparing frequency output\n" ;
$COUNT_OUTPUT = 0;
while ($COUNT_OUTPUT < $NUMBER_CONSENSUS_SEQ)       {
       my $CURRENT_CONSENSUS = $ARRAY_CONSENSUS[$COUNT_OUTPUT] ;
       my $FREQUENCY = $HASH_COUNT{$COUNT_OUTPUT} ;
       $OUTPUT_SUMMARY = $OUTPUT_SUMMARY.="Frequency of $CURRENT_CONSENSUS:\t$FREQUENCY\n" ;
       $COUNT_OUTPUT = $COUNT_OUTPUT + 1 ;
                                                                                          }

print "Preparing output\n" ;
$COUNT_OUTPUT_2 = $HIGHEST_NUMBER_DASHES ;
while ($COUNT_OUTPUT_2 > 0)   {
       my $INFO_TO_OUTPUT = $HASH_POSITION_BASED_ON_DASHES{$COUNT_OUTPUT_2} ;
       chomp ($INFO_TO_OUTPUT) ;
       my $SIZE_INFO_TO_OUTPUT = length($INFO_TO_OUTPUT) ;
       if ($SIZE_INFO_TO_OUTPUT > 0)        {
              $OUTPUT_FOUND = $OUTPUT_FOUND.="$INFO_TO_OUTPUT\n" ;
                                                                      }
       $COUNT_OUTPUT_2 = $COUNT_OUTPUT_2 - 1 ;
                                                              }

open (OUTPUT, ">OUTPUT_C2_region_adjusted_$INPUT_FILE_NAME") or die "Can't open $file for
reading" ;
print OUTPUT "$OUTPUT_SUMMARY" ;
print OUTPUT "$OUTPUT_FOUND";
close (OUTPUT);

open (OUTPUT_2, ">OUTPUT_C2_region_MULTIPLE_copies_$INPUT_FILE_NAME") or die "Can't open $file
for reading" ;
print OUTPUT_2 "Sequences with multiple copies of consensus\n" ;
print OUTPUT_2 "$OUTPUT_MULTIPLE_CONSENSUS\n" ;
close (OUTPUT_2);
```

```
open (OUTPUT_3, ">OUTPUT_C2_region_NOT_FOUND_$INPUT_FILE_NAME") or die "Can't open $file for
reading" ;
print OUTPUT_3 "Sequences with no copies of consensus\n" ;
print OUTPUT_3 "$OUTPUT_NOT_FOUND";
close (OUTPUT_3) ;
```

B4.16S_C2_region_alignment_check

```
$INPUT_FILE_NAME = "Input_file_name.phylip" ;
open (PHYLIP, "$INPUT_FILE_NAME") or die "Can't open $file for reading" ;

$POSITION_TO_CHECK = 287 ;
$LENGTH_TO_CHECK = 2 ;
$PATTERN =  "--" ;
$LENGTH_PATTERN = length($PATTERN) ;


$OUTPUT_OK = "" ; $OUTPUT_NOT_OK = "" ;

while (<PHYLIP>)       {
       $READ_COUNT = $READ_COUNT + 1 ; my $COUNT_THOUSAND = $READ_COUNT/2000 ;
       my $COUNT_INTEGER = int($COUNT_THOUSAND) ; my $INTEGER_CHECK = $COUNT_THOUSAND -
$COUNT_INTEGER;
       if ($INTEGER_CHECK == 0) {print "Checking sequence $READ_COUNT\n";  }

       my $LINE = $_ ;        chomp ($LINE);
       if ($LINE =~ />/)      {
              my @ARRAY_LINE = split('\t',$LINE) ;
              my $NAME = $ARRAY_LINE[0] ;   my $SEQ = $ARRAY_LINE[1] ; my $LENGTH_SEQ =
length($SEQ) ;

              my $REGION_TO_CHECK = substr($SEQ,$POSITION_TO_CHECK,$LENGTH_TO_CHECK) ;
              if ($REGION_TO_CHECK ne $PATTERN) {$OUTPUT_NOT_OK = $OUTPUT_NOT_OK.="$LINE\n" ; }
              if ($REGION_TO_CHECK eq $PATTERN)     {
                     my $SECOND_CHECK = 0 ;
                     my $UPSTREAM_POSITION = $POSITION_TO_CHECK - 1 ;
                     my $UPSTREAM_TO_CHECK = substr($SEQ,$UPSTREAM_POSITION,1) ;
                     if ($UPSTREAM_TO_CHECK eq "-") {$SECOND_CHECK = 2 ;}

                     my $DOWNSTREAM_POSITION = $POSITION_TO_CHECK + $LENGTH_PATTERN ;
                     my $DOWNSTREAM_TO_CHECK = substr($SEQ,$DOWNSTREAM_POSITION,1) ;
                     if ($DOWNSTREAM_TO_CHECK eq "-") {$SECOND_CHECK = 2 ;}

                     if ($SECOND_CHECK < 1)        {
                            $OUTPUT_OK = $OUTPUT_OK.="$LINE\n" ;
                                                                      }
                     if ($SECOND_CHECK > 1)        {
                            $OUTPUT_NOT_OK = $OUTPUT_NOT_OK.="$LINE\n" ;
                                                                      }
                                                                                    }
                                          }
                                   }
close (PHYLIP);

print "Preparing frequency output\n" ;
$COUNT_OUTPUT = 0;
while ($COUNT_OUTPUT < $NUMBER_CONSENSUS_SEQ)       {
       my $CURRENT_CONSENSUS = $ARRAY_CONSENSUS[$COUNT_OUTPUT] ;
       my $FREQUENCY = $HASH_COUNT{$COUNT_OUTPUT} ;
       $OUTPUT_SUMMARY = $OUTPUT_SUMMARY.="Frequency of $CURRENT_CONSENSUS:\t$FREQUENCY\n" ;
       $COUNT_OUTPUT = $COUNT_OUTPUT + 1 ;
                                                                                    }

open (OUTPUT, ">OUTPUT_C2_region_confirmed_$INPUT_FILE_NAME") or die "Can't open $file for
reading" ;
#print OUTPUT "Consensus and variants are:\n$CONSENSUS_VARIANT_LIST\n";
print OUTPUT "$OUTPUT_OK";
close (OUTPUT);

open (OUTPUT_2, ">OUTPUT_C2_region_to_check_$INPUT_FILE_NAME") or die "Can't open $file for
reading" ;
print OUTPUT_2 "$OUTPUT_NOT_OK";
close (OUTPUT_2);
```

B5.16S_C3_begin_region_position_adjustment

```
$INPUT_FILE_NAME = "Input_file_name.phylip" ;
open (PHYLIP, "$INPUT_FILE_NAME") or die "Can't open $file for reading" ;

$V2_TOTAL_LENGTH = 150 ;
$CONSENSUS_1A =  ".CG....ATTAG.T.GTTGGT......" ;            #C3a begin
$CONSENSUS_1B =  ".CG....GTTAG.T.GTTGGC......";             #other 03102019
$CONSENSUS_2 =  ".CG....ATTAG.T.GT.GG.....TA" ;      #C3b begin
$CONSENSUS_3 =  "A.A....ATTAG.T.GT.GG.....TA" ;      #C3c begin
$CONSENSUS_4 =  "G.G....ATTAG...G..GG.....C.";       #C3d begin
$CONSENSUS_5 =  "GC.....AT.AG.T.GTTGG.....TA" ; #BSTP
$CONSENSUS_21 = ".......AT.AG.T.GTTGG.....TA" ;      #BSTP
$CONSENSUS_22 = "G......ATTA....G..GG......A" ;         #GCGTCTGATTAACTAGTTGGTAAGGTA
$CONSENSUS_23 = "G......A.TAG...G..GG......A" ;         #GCGTTCCAGTAGCTAGTTGGTGAGGTA
$CONSENSUS_24 = "...........G.T.GT.G......TA";
$CONSENSUS_25 = "GCG.........T......T...GTA";
$CONSENSUS_26 = ".......AT......GTTGG.....TA";
$CONSENSUS_27 = "GC.....ATTAG.T.G....T....TA";

$CONSENSUS_31= "GCG.........T..........GTA" ;
$CONSENSUS_32= "G.G.........T..........GTA" ;
$CONSENSUS_33= ".CG.........T...........TA" ;
$CONSENSUS_34= ".GG.........T.G..GG.....CA" ;
$CONSENSUS_35= ".G..........T.G..G......CA" ;

$CONSENSUS_LAST1 = ".......ATTAG...G..GG.......";
$CONSENSUS_LAST2 = ".......AT.AG...G..GG.......";

#Lee's consensus sequences
#$CONSENSUS_6 = "CGGCCCATCAGTTAGTTGGTGAGGGTA";
#$CONSENSUS_7 = "GCGGCCCATTAGCTTGTCGGCGGGGC.";
#$CONSENSUS_8 = "GCGTCCGATTAGCTAGTTGGCGGGGCA";
#$CONSENSUS_9 = "CG....ATTAG.TAGTTG.....GGTA";
#$CONSENSUS_10 = "CGTCCTATCAG.TAGTTGGTTGAGGTA";
#$CONSENSUS_11 = "GCGTC.GATTA.CT..TTGG.G.GGTA";
#$CONSENSUS_12 = "GCGTCCTACCAGATAGTTGGCGGGGTA" ;
#$CONSENSUS_13 = "GTATTGTATCAGTTAGTTGGTGGGGTA" ;

$LIST_CONSENSUS =
"$CONSENSUS_1A,$CONSENSUS_1B,$CONSENSUS_2,$CONSENSUS_3,$CONSENSUS_4,$CONSENSUS_5" ;
$LIST_CONSENSUS =
$LIST_CONSENSUS.=",$CONSENSUS_21,$CONSENSUS_22,$CONSENSUS_23,$CONSENSUS_24,$CONSENSUS_25" ;
$LIST_CONSENSUS = $LIST_CONSENSUS.=",$CONSENSUS_26,$CONSENSUS_27" ;
$LIST_CONSENSUS = $LIST_CONSENSUS.=",$CONSENSUS_LAST1,$CONSENSUS_LAST2" ;
$LIST_CONSENSUS =
$LIST_CONSENSUS.=",$CONSENSUS_31,$CONSENSUS_32,$CONSENSUS_33,$CONSENSUS_34,$CONSENSUS_35" ;
@ARRAY_LIST = split(',', $LIST_CONSENSUS) ; $NUMBER_CONSENSUS = scalar(@ARRAY_LIST) ;

%HASH_COUNT ; $COUNT_HASH_SET_UP = 0 ;
while ($COUNT_HASH_SET_UP < $NUMBER_CONSENSUS)       {
        $HASH_COUNT{$COUNT_HASH_SET_UP} = 0 ;
        $COUNT_HASH_SET_UP = $COUNT_HASH_SET_UP + 1 ;
                                                                                }

$OUTPUT_NOT_FOUND = "" ; $OUTPUT_FOUND = "" ; $OUTPUT_SUMMARY = "" ;
$READ_COUNT = 0 ; $COUNT_NOT_FOUND = 0 ;
while (<PHYLIP>)       {
        $READ_COUNT = $READ_COUNT + 1 ; my $COUNT_THOUSAND = $READ_COUNT/2000 ;
        my $COUNT_INTEGER = int($COUNT_THOUSAND) ; my $INTEGER_CHECK = $COUNT_THOUSAND -
$COUNT_INTEGER;
        if ($INTEGER_CHECK == 0) {print "Checking sequence $READ_COUNT\n";  }

        my $LINE = $_ ;        chomp ($LINE); my @ARRAY_LINE = split('\t',$LINE) ;
        my $NAME = $ARRAY_LINE[0] ;   my $SEQ = $ARRAY_LINE[1] ; my $LENGTH_SEQ = length($SEQ) ;

        my @ARRAY_SEQ_BLOCKS = split('-', $SEQ) ;
        my $NUMBER_OF_BLOCKS = scalar(@ARRAY_SEQ_BLOCKS) ;
        my $SECOND_TO_LAST_BLOCK = $NUMBER_OF_BLOCKS - 1 ;
        my $LENGTH_FIVE_PRIME = 0 ;
        my $COUNT_BLOCK = 0 ; my $REBUILDING_SEQUENCE = "" ;
```

```
        while ($COUNT_BLOCK < $SECOND_TO_LAST_BLOCK)        {
                my $BLOCK = $ARRAY_SEQ_BLOCKS[$COUNT_BLOCK] ;
                $REBUILDING_SEQUENCE = $REBUILDING_SEQUENCE.="$BLOCK-" ;
                $COUNT_BLOCK = $COUNT_BLOCK + 1 ;

        }
        my $LENGTH_FIVE_PRIME = length($REBUILDING_SEQUENCE) ;
        my $DOWNSTREAM_REGION_TO_SCREEN = $ARRAY_SEQ_BLOCKS[$SECOND_TO_LAST_BLOCK ] ;
        my $LENGTH_DOWNSTREAM_TO_SCREEN = length($DOWNSTREAM_REGION_TO_SCREEN) ;
        my $COUNT_ARRAY = 0 ; my $CONSENSUS_FOUND = 0 ;
        while ($COUNT_ARRAY < $NUMBER_CONSENSUS and $CONSENSUS_FOUND < 1)   {
                my $CURRENT_CONSENSUS = $ARRAY_LIST[$COUNT_ARRAY] ;
                my $LENGTH_CONSENSUS = length($CURRENT_CONSENSUS) ;
                if ($DOWNSTREAM_REGION_TO_SCREEN =~ /$CURRENT_CONSENSUS/)   {
                        $CONSENSUS_FOUND = 2 ;
                        my $INFO_TO_UPDATE = $HASH_COUNT{$COUNT_ARRAY} ;
                        $INFO_TO_UPDATE = $INFO_TO_UPDATE + 1 ;
                        $HASH_COUNT{$COUNT_ARRAY} = $INFO_TO_UPDATE ;


                        my $FIVE_PRIME_BOUNDARY =  0 ; my $THREE_PRIME_BOUNDARY = 0 ;
                        my $DOWNSTREAM_LENGTH = 0 ; my $THREE_PRIME_FOUND =  0 ;
                        my $LENGTH_TO_SCREEN = length($CURRENT_CONSENSUS) ;
                        while ($THREE_PRIME_FOUND < 1)       {
                                my $DOWNSTREAM_SEARCH = substr($DOWNSTREAM_REGION_TO_SCREEN, 0,
$LENGTH_TO_SCREEN) ;
                                if ($DOWNSTREAM_SEARCH !~ /$CURRENT_CONSENSUS/)      {
                                        $LENGTH_TO_SCREEN = $LENGTH_TO_SCREEN + 1 ;

                        }
                                if ($DOWNSTREAM_SEARCH =~ /$CURRENT_CONSENSUS/)      {
                                        $FIVE_PRIME_BOUNDARY = $LENGTH_TO_SCREEN -
$LENGTH_CONSENSUS;
                                        $THREE_PRIME_BOUNDARY = $LENGTH_TO_SCREEN ;
                                        $DOWNSTREAM_LENGTH = $LENGTH_DOWNSTREAM_TO_SCREEN -
$THREE_PRIME_BOUNDARY ;
                                        $THREE_PRIME_FOUND =  2 ;

                        }

                                                                                                }
                        my $FIVE_PRIME_REGION = substr($SEQ, 0, $LENGTH_FIVE_PRIME) ;
                        my $CONSERVED_REGION =
substr($DOWNSTREAM_REGION_TO_SCREEN,$FIVE_PRIME_BOUNDARY, $LENGTH_CONSENSUS) ;
#                       $OUTPUT_FOUND = $OUTPUT_FOUND.="$CONSERVED_REGION\n" ;
#                       my $LENGTH_V2_NOT_ALIGNED = $FIVE_PRIME_BOUNDARY - $LENGTH_FIVE_PRIME ;
                        my $LENGTH_V2_NOT_ALIGNED = $FIVE_PRIME_BOUNDARY ;
                        my $V2 = substr($DOWNSTREAM_REGION_TO_SCREEN,0, $LENGTH_V2_NOT_ALIGNED);
                        my $NUMBER_OF_DASHES = $V2_TOTAL_LENGTH - $LENGTH_V2_NOT_ALIGNED ;
                        my $DASHES_COUNT = 0 ; my $V2_DASHES = "" ;
                        while ($DASHES_COUNT < $NUMBER_OF_DASHES)    {
                                $V2_DASHES = $V2_DASHES.="-" ;
                                $DASHES_COUNT = $DASHES_COUNT + 1 ;

        }
                        my $DOWNSTREAM = substr($DOWNSTREAM_REGION_TO_SCREEN,$THREE_PRIME_BOUNDARY
,$DOWNSTREAM_LENGTH) ;
                        my $ALIGNED_SEQ = "$FIVE_PRIME_REGION-$V2_DASHES-$V2-$CONSERVED_REGION-
$DOWNSTREAM"  ;
#                       $OUTPUT_FOUND = $OUTPUT_FOUND.="$LINE\n" ;
                        $OUTPUT_FOUND = $OUTPUT_FOUND.="$NAME\t$ALIGNED_SEQ\n" ;

                                }
                $COUNT_ARRAY = $COUNT_ARRAY + 1 ;

                                        }

        if ($CONSENSUS_FOUND < 1)      {
#               $COUNT_NOT_FOUND = $COUNT_NOT_FOUND + 1 ;
                $OUTPUT_NOT_FOUND = $OUTPUT_NOT_FOUND."$LINE\n" ;
                                                                        }
```

```
                              }
close (PHYLIP);
#####################################
print "Preparing frequency output\n" ;
$COUNT_OUTPUT = 0;
while ($COUNT_OUTPUT < $NUMBER_CONSENSUS)    {
       my $CURRENT_CONSENSUS = $ARRAY_LIST[$COUNT_OUTPUT] ;
       my $FREQUENCY = $HASH_COUNT{$COUNT_OUTPUT} ;
       $OUTPUT_SUMMARY = $OUTPUT_SUMMARY.="Frequency of $CURRENT_CONSENSUS:\t$FREQUENCY\n" ;
       $COUNT_OUTPUT = $COUNT_OUTPUT + 1 ;
                                                                                 }



open (OUTPUT, ">OUTPUT_C3_begin_adjusted_$INPUT_FILE_NAME") or die "Can't open $file for reading"
;
#print OUTPUT "Consensus and variants are:\n$CONSENSUS_VARIANT_LIST\n";
print OUTPUT "$OUTPUT_SUMMARY" ;
print OUTPUT "$OUTPUT_FOUND";
close (OUTPUT);

open (OUTPUT_2, ">OUTPUT_C3_begin_NOT_FOUND_$INPUT_FILE_NAME") or die "Can't open $file for
reading" ;
print OUTPUT_2 "$OUTPUT_NOT_FOUND";
close (OUTPUT_2);
```

B6.16S_C3_begin_region_alignment_check

```
$INPUT_FILE_NAME = "Input_file_name.phylip " ;
open (PHYLIP, "$INPUT_FILE_NAME") or die "Can't open $file for reading" ;

$POSITION_TO_CHECK = 441 ;
$LENGTH_TO_CHECK = 29 ;
$PATTERN_TEST =  "-GCGGTGCATTAGTTAGTTGGTGGGGTA-" ;
$PATTERN =       "-...........................-" ;

$OUTPUT_OK = "" ; $OUTPUT_NOT_OK = "" ;
#print "test\n" ;
#if ($PATTERN_TEST !~ /$PATTERN/) {$OUTPUT_NOT_OK = $OUTPUT_NOT_OK.="test pattern fails\n" ; }
#if ($PATTERN_TEST =~ /$PATTERN/)     {$OUTPUT_OK = $OUTPUT_OK.="test pattern passed\n" ; }


while (<PHYLIP>)       {
        $READ_COUNT = $READ_COUNT + 1 ; my $COUNT_THOUSAND = $READ_COUNT/2000 ;
        my $COUNT_INTEGER = int($COUNT_THOUSAND) ; my $INTEGER_CHECK = $COUNT_THOUSAND - 
$COUNT_INTEGER;
        if ($INTEGER_CHECK == 0) {print "Checking sequence $READ_COUNT\n";  }

        my $LINE = $_ ;        chomp ($LINE); my @ARRAY_LINE = split('\t',$LINE) ;
        my $NAME = $ARRAY_LINE[0] ;   my $SEQ = $ARRAY_LINE[1] ; my $LENGTH_SEQ = length($SEQ) ;

        my $REGION_TO_CHECK = substr($SEQ,$POSITION_TO_CHECK,$LENGTH_TO_CHECK) ;
        if ($REGION_TO_CHECK !~ /$PATTERN/) {$OUTPUT_NOT_OK = $OUTPUT_NOT_OK.="$LINE\n" ; }
        if ($REGION_TO_CHECK =~ /$PATTERN/)   {
               my $DOWNSTREAM_START = $POSITION_TO_CHECK + $LENGTH_TO_CHECK ;
               my $DOWNSTREAM_LENGTH = $LENGTH_SEQ - $DOWNSTREAM_START ;
               my $DOWNSTREAM_REGION = substr($SEQ,$DOWNSTREAM_START,$DOWNSTREAM_LENGTH) ;
               if ($DOWNSTREAM_REGION =~ /-/)       {
                      $OUTPUT_NOT_OK = $OUTPUT_NOT_OK.="$LINE\n" ;
                                                                            }
               if ($DOWNSTREAM_REGION !~ /-/)               {

                      $OUTPUT_OK = $OUTPUT_OK.="$LINE\n" ;
                                                                                   }
                                                                            }
                                      }
close (PHYLIP);

print "Preparing frequency output\n" ;
$COUNT_OUTPUT = 0;
while ($COUNT_OUTPUT < $NUMBER_CONSENSUS_SEQ)       {
        my $CURRENT_CONSENSUS = $ARRAY_CONSENSUS[$COUNT_OUTPUT] ;
        my $FREQUENCY = $HASH_COUNT{$COUNT_OUTPUT} ;
        $OUTPUT_SUMMARY = $OUTPUT_SUMMARY.="Frequency of $CURRENT_CONSENSUS:\t$FREQUENCY\n" ;
        $COUNT_OUTPUT = $COUNT_OUTPUT + 1 ;
                                                                                   }

open (OUTPUT, ">OUTPUT_C3_begin_confirmed_$INPUT_FILE_NAME") or die "Can't open $file for 
reading" ;
#print OUTPUT "Consensus and variants are:\n$CONSENSUS_VARIANT_LIST\n";
print OUTPUT "$OUTPUT_OK";
close (OUTPUT);

open (OUTPUT_2, ">OUTPUT_C3_begin_to_check_$INPUT_FILE_NAME") or die "Can't open $file for 
reading" ;
print OUTPUT_2 "$OUTPUT_NOT_OK";
close (OUTPUT_2);
```

B7.16S_C3_end_region_position_adjustment

```
$INPUT_FILE_NAME = "Input_file_name.phylip " ;
open (PHYLIP, "$INPUT_FILE_NAME") or die "Can't open $file for reading" ;

#$C3_LENGTH_MODEL_ALIGN = 59 ; ?? do I need this ???

#$CONSENSUS = "AT.TT...CAA.GG..G.....C..A.......A" ; # this sequence is downstream from
Consensus_1 to 15
#other tested: "TTGTAAACTGCT" ;
$CONSENSUS_1 =  "TTGTAAA....." ;
$CONSENSUS_2a = ".TGTAAA....." ;
$CONSENSUS_2 =  "T.GTAAA....." ;
#$CONSENSUS_3 =  "TT.TAAA....." ; #SUBSTITUTED ON 09052018
$CONSENSUS_3 =  "T..TAAA....." ; #MODIFIED ON 09052018
$CONSENSUS_4 =  "TTG.AAA....." ;
$CONSENSUS_5 =  "TTGT.AA....." ;
$CONSENSUS_6 =  "TTGTA.A....." ;
$CONSENSUS_7 =  "TTGTAA......" ;
$CONSENSUS_8 =  "ACGTAAA....." ;
$CONSENSUS_9 =  ".CGTAAA....." ;
$CONSENSUS_10 = "A.GTAAA....." ;
$CONSENSUS_11 = "AC.TAAA....." ;
$CONSENSUS_12 = "ACG.AAA....." ;
$CONSENSUS_13 = "ACGT.AA....." ;
$CONSENSUS_14 = "ACGTA.A....." ;
$CONSENSUS_15 = "ACGTAA......" ;
$CONSENSUS_16 = "TCGTAAG....." ; #latest addition tested 09052018
$CONSENSUS_17 = "TCTTAAA....." ; #latest addition tested 09052018
#Other to try: "TCGTGAA....."
#$LIST_CONSENSUS = "$CONSENSUS";
$LIST_CONSENSUS =
"$CONSENSUS_1,$CONSENSUS_2,$CONSENSUS_2a,$CONSENSUS_3,$CONSENSUS_4,$CONSENSUS_5,$CONSENSUS_6,$CON
SENSUS_7,$CONSENSUS_8" ;
$LIST_CONSENSUS =
$LIST_CONSENSUS.=",$CONSENSUS_9,$CONSENSUS_10,$CONSENSUS_11,$CONSENSUS_12,$CONSENSUS_13,$CONSENSU
S_14,$CONSENSUS_15" ;
$LIST_CONSENSUS = $LIST_CONSENSUS.=",$CONSENSUS_16,$CONSENSUS_17" ; #latest addition tested
09052018
@ARRAY_LIST = split(',', $LIST_CONSENSUS) ; $NUMBER_CONSENSUS = scalar(@ARRAY_LIST) ;

%HASH_COUNT ; $COUNT_HASH_SET_UP = 0 ;
while ($COUNT_HASH_SET_UP < $NUMBER_CONSENSUS)       {
        $HASH_COUNT{$COUNT_HASH_SET_UP} = 0 ;
        $COUNT_HASH_SET_UP = $COUNT_HASH_SET_UP + 1 ;
                                                                                                    }

$OUTPUT_NOT_FOUND = "" ; $OUTPUT_FOUND = "" ; $OUTPUT_SUMMARY = "" ;
$READ_COUNT = 0 ; $COUNT_NOT_FOUND = 0 ;

while (<PHYLIP>)       {
        $READ_COUNT = $READ_COUNT + 1 ; my $COUNT_THOUSAND = $READ_COUNT/2000 ;
        my $COUNT_INTEGER = int($COUNT_THOUSAND) ; my $INTEGER_CHECK = $COUNT_THOUSAND -
$COUNT_INTEGER;
        if ($INTEGER_CHECK == 0) {print "Checking sequence $READ_COUNT\n";  }

        my $LINE = $_ ;        chomp ($LINE); my @ARRAY_LINE = split('\t',$LINE) ;
        my $NAME = $ARRAY_LINE[0] ;   my $SEQ = $ARRAY_LINE[1] ; my $LENGTH_SEQ = length($SEQ) ;

        my @ARRAY_SEQ_BLOCKS = split('-', $SEQ) ;
        my $NUMBER_OF_BLOCKS = scalar(@ARRAY_SEQ_BLOCKS) ;
        my $LAST_BLOCK = $NUMBER_OF_BLOCKS - 1 ;
        my $LENGTH_FIVE_PRIME = 0 ;
        my $COUNT_BLOCK = 0 ; my $REBUILDING_SEQUENCE = "" ;
        while ($COUNT_BLOCK < $LAST_BLOCK) {
                my $BLOCK = $ARRAY_SEQ_BLOCKS[$COUNT_BLOCK] ;
                $REBUILDING_SEQUENCE = $REBUILDING_SEQUENCE.="$BLOCK-" ;
                $COUNT_BLOCK = $COUNT_BLOCK + 1 ;
                                                                                                    }
        my $LENGTH_FIVE_PRIME = length($REBUILDING_SEQUENCE) ;
        my $DOWNSTREAM_REGION_TO_SCREEN = $ARRAY_SEQ_BLOCKS[$LAST_BLOCK ] ;
```

```
        my $LENGTH_DOWNSTREAM_TO_SCREEN = length($DOWNSTREAM_REGION_TO_SCREEN) ;
        my $COUNT_ARRAY = 0 ; my $CONSENSUS_FOUND = 0 ;
        while ($COUNT_ARRAY < $NUMBER_CONSENSUS and $CONSENSUS_FOUND < 1)   {
                my $CURRENT_CONSENSUS = $ARRAY_LIST[$COUNT_ARRAY] ;
                my $LENGTH_CONSENSUS = length($CURRENT_CONSENSUS) ;
                if ($DOWNSTREAM_REGION_TO_SCREEN =~ /$CURRENT_CONSENSUS/)   {
                        $CONSENSUS_FOUND = 2 ;
                        my $INFO_TO_UPDATE = $HASH_COUNT{$COUNT_ARRAY} ;
                        $INFO_TO_UPDATE = $INFO_TO_UPDATE + 1 ;
                        $HASH_COUNT{$COUNT_ARRAY} = $INFO_TO_UPDATE ;


                        my $FIVE_PRIME_BOUNDARY =  0 ; my $THREE_PRIME_BOUNDARY = 0 ;
                        my $THREE_PRIME_LENGTH = 0 ; my $THREE_PRIME_FOUND =  0 ;
                        my $LENGTH_TO_SCREEN = length($CURRENT_CONSENSUS) ;
                        while ($THREE_PRIME_FOUND < 1)       {
                                my $DOWNSTREAM_SEARCH = substr($DOWNSTREAM_REGION_TO_SCREEN, 0,
$LENGTH_TO_SCREEN) ;
                                if ($DOWNSTREAM_SEARCH !~ /$CURRENT_CONSENSUS/)      {
                                        $LENGTH_TO_SCREEN = $LENGTH_TO_SCREEN + 1 ;

                        }
                                if ($DOWNSTREAM_SEARCH =~ /$CURRENT_CONSENSUS/)      {
                                        $FIVE_PRIME_BOUNDARY = $LENGTH_TO_SCREEN -
$LENGTH_CONSENSUS;
                                        $THREE_PRIME_BOUNDARY = $LENGTH_TO_SCREEN ;
                                        $THREE_PRIME_LENGTH = $LENGTH_DOWNSTREAM_TO_SCREEN -
$THREE_PRIME_BOUNDARY ;
                                        $THREE_PRIME_FOUND =  2 ;

                        }

                                                                        }
                        my $FIVE_PRIME_REGION = substr($SEQ, 0, $LENGTH_FIVE_PRIME) ;
                        my $C3 = substr($DOWNSTREAM_REGION_TO_SCREEN,0, $LENGTH_TO_SCREEN) ;
                        my $DOWNSTREAM = substr($DOWNSTREAM_REGION_TO_SCREEN,$THREE_PRIME_BOUNDARY
,$THREE_PRIME_LENGTH) ;
                        my $ALIGNED_SEQ = "$FIVE_PRIME_REGION-$C3-$DOWNSTREAM"  ;
#                       $OUTPUT_FOUND = $OUTPUT_FOUND.="$LINE\n" ; ##FOR CHECKING ON SCRIPT
ACCURACY
                        $OUTPUT_FOUND = $OUTPUT_FOUND.="$NAME\t$ALIGNED_SEQ\n" ;

                                }
                $COUNT_ARRAY = $COUNT_ARRAY + 1 ;


                                        }
        if ($CONSENSUS_FOUND < 1)      {
#               $COUNT_NOT_FOUND = $COUNT_NOT_FOUND + 1 ;
                $OUTPUT_NOT_FOUND = $OUTPUT_NOT_FOUND."$LINE\n" ;
                                                        }
                                        }
close (PHYLIP);

print "Preparing frequency output\n" ;
$COUNT_OUTPUT = 0;
while ($COUNT_OUTPUT < $NUMBER_CONSENSUS)    {
        my $CURRENT_CONSENSUS = $ARRAY_LIST[$COUNT_OUTPUT] ;
        my $FREQUENCY = $HASH_COUNT{$COUNT_OUTPUT} ;
        $OUTPUT_SUMMARY = $OUTPUT_SUMMARY.="Frequency of $CURRENT_CONSENSUS:\t$FREQUENCY\n" ;
        $COUNT_OUTPUT = $COUNT_OUTPUT + 1 ;
                                                                        }
open (OUTPUT, ">OUTPUT_C3_end_position_adjusted_$INPUT_FILE_NAME") or die "Can't open $file for
reading" ;
print OUTPUT "$OUTPUT_SUMMARY" ;
print OUTPUT "$OUTPUT_FOUND";
close (OUTPUT);
open (OUTPUT_2, ">OUTPUT_C3_end_position_NOT_FOUND_$INPUT_FILE_NAME") or die "Can't open $file
for reading" ;
print OUTPUT_2 "$OUTPUT_NOT_FOUND";
close (OUTPUT_2);
```

B8.16S_C4_begin_region_position_adjustment

```
$INPUT_FILE_NAME = "Input_file_name.phylip" ;
open (PHYLIP, "$INPUT_FILE_NAME") or die "Can't open $file for reading" ;

$V3_TOTAL_LENGTH = 150 ; #ORIGINALLY USED: GAATAAG.ATCGGC
$CONSENSUS_1A = "CGGCTAA.T" ; $CONSENSUS_1B = ".GGCTAA.T" ; $CONSENSUS_1C = "C.GCTAA.T" ;
$CONSENSUS_1D = "CG.CTAA.T"; $CONSENSUS_1E= "CGG.TAA.T"; $CONSENSUS_1F = "CGGC.AA.T" ;
$CONSENSUS_1G = "CGGCT.A.T" ; $CONSENSUS_1H = "CGGCTA..T" ; $CONSENSUS_1I = "CGGCTAA.." ;
$CONSENSUS_2A = "T.ACAAAAC" ;$CONSENSUS_2B = "TG.CTAAAC" ;$CONSENSUS_2C = "TGA.TAAAC" ;
$CONSENSUS_2D = "TGAC.AAAC" ;$CONSENSUS_2E = "TGACT.AAC" ;$CONSENSUS_2F = "TGACTA.AC" ;
$CONSENSUS_2G = "TGACTAA.C" ;$CONSENSUS_2H = "TGACTAAA." ;$CONSENSUS_2I = ".GACTAAAC" ;
$CONSENSUS_3A = ".GTTTGATC" ;$CONSENSUS_3B = "A.TTTGATC" ;$CONSENSUS_3C = "AG.TTGATC" ;
$CONSENSUS_3D = "AGT.TGATC" ;$CONSENSUS_3E = "AGTT.GATC" ;$CONSENSUS_3F = "AGTTT.ATC" ;
$CONSENSUS_3G = "AGTTTG.TC" ;$CONSENSUS_3H = "AGTTTGA.C" ;$CONSENSUS_3I = "AGTTTGAT." ;
$CONSENSUS_4A = ".TACTAACT" ; $CONSENSUS_4B = "T.ACTAACT" ; $CONSENSUS_4C = "TT.CTAACT" ;
$CONSENSUS_4D = "TTA.TAACT" ; $CONSENSUS_4E = "TTAC.AACT" ; $CONSENSUS_4F = "TTACT.ACT" ;
$CONSENSUS_4G = "TTACTA.CT" ; $CONSENSUS_4H = "TTACTAA.T" ; $CONSENSUS_4I = "TTACTAAC." ;

$LIST_CONSENSUS = "$CONSENSUS_1A,$CONSENSUS_1B,$CONSENSUS_1C,$CONSENSUS_1D,$CONSENSUS_1E" ;
$LIST_CONSENSUS = $LIST_CONSENSUS.=",$CONSENSUS_1F,$CONSENSUS_1G,$CONSENSUS_1H,$CONSENSUS_1I" ;
$LIST_CONSENSUS =
$LIST_CONSENSUS.=",$CONSENSUS_2A,$CONSENSUS_2B,$CONSENSUS_2C,$CONSENSUS_2D,$CONSENSUS_2E" ;
$LIST_CONSENSUS = $LIST_CONSENSUS.=",$CONSENSUS_2F,$CONSENSUS_2G,$CONSENSUS_2H,$CONSENSUS_2I" ;
$LIST_CONSENSUS =
$LIST_CONSENSUS.=",$CONSENSUS_3A,$CONSENSUS_3B,$CONSENSUS_3C,$CONSENSUS_3D,$CONSENSUS_3E" ;
$LIST_CONSENSUS = $LIST_CONSENSUS.=",$CONSENSUS_3F,$CONSENSUS_3G,$CONSENSUS_3H,$CONSENSUS_3I" ;
$LIST_CONSENSUS =
$LIST_CONSENSUS.=",$CONSENSUS_4A,$CONSENSUS_4B,$CONSENSUS_4C,$CONSENSUS_4D,$CONSENSUS_4E" ;
$LIST_CONSENSUS = $LIST_CONSENSUS.=",$CONSENSUS_4F,$CONSENSUS_4G,$CONSENSUS_4H,$CONSENSUS_4I" ;

@ARRAY_LIST = split(',', $LIST_CONSENSUS) ; $NUMBER_CONSENSUS = scalar(@ARRAY_LIST) ;

%HASH_COUNT ; $COUNT_HASH_SET_UP = 0 ;
while ($COUNT_HASH_SET_UP < $NUMBER_CONSENSUS)      {
        $HASH_COUNT{$COUNT_HASH_SET_UP} = 0 ;
        $COUNT_HASH_SET_UP = $COUNT_HASH_SET_UP + 1 ;
                                                                                                  }

$OUTPUT_NOT_FOUND = "" ; $OUTPUT_FOUND = "" ; $OUTPUT_SUMMARY = "" ;
$READ_COUNT = 0 ; $COUNT_NOT_FOUND = 0 ;
while (<PHYLIP>)      {
        $READ_COUNT = $READ_COUNT + 1 ; my $COUNT_THOUSAND = $READ_COUNT/2000 ;
        my $COUNT_INTEGER = int($COUNT_THOUSAND) ; my $INTEGER_CHECK = $COUNT_THOUSAND -
$COUNT_INTEGER;
        if ($INTEGER_CHECK == 0) {print "Checking sequence $READ_COUNT\n";  }

        my $LINE = $_ ;       chomp ($LINE); my @ARRAY_LINE = split('\t',$LINE) ;
        my $NAME = $ARRAY_LINE[0] ;   my $SEQ = $ARRAY_LINE[1] ; my $LENGTH_SEQ = length($SEQ) ;

        my @ARRAY_SEQ_BLOCKS = split('-', $SEQ) ;
        my $NUMBER_OF_BLOCKS = scalar(@ARRAY_SEQ_BLOCKS) ;
        my $LAST_BLOCK = $NUMBER_OF_BLOCKS - 1 ;
        my $LENGTH_FIVE_PRIME = 0 ;
        my $COUNT_BLOCK = 0 ; my $REBUILDING_SEQUENCE = "" ;
        while ($COUNT_BLOCK < $LAST_BLOCK)    {
                my $BLOCK = $ARRAY_SEQ_BLOCKS[$COUNT_BLOCK] ;
                $REBUILDING_SEQUENCE = $REBUILDING_SEQUENCE.="$BLOCK-" ;
                $COUNT_BLOCK = $COUNT_BLOCK + 1 ;

        }
        my $LENGTH_FIVE_PRIME = length($REBUILDING_SEQUENCE) ;
        my $DOWNSTREAM_REGION_TO_SCREEN = $ARRAY_SEQ_BLOCKS[$LAST_BLOCK ] ;
        my $LENGTH_DOWNSTREAM_TO_SCREEN = length($DOWNSTREAM_REGION_TO_SCREEN) ;
        my $COUNT_ARRAY = 0 ; my $CONSENSUS_FOUND = 0 ;
        while ($COUNT_ARRAY < $NUMBER_CONSENSUS and $CONSENSUS_FOUND < 1)   {
                my $CURRENT_CONSENSUS = $ARRAY_LIST[$COUNT_ARRAY] ;
                my $LENGTH_CONSENSUS = length($CURRENT_CONSENSUS) ;
                if ($DOWNSTREAM_REGION_TO_SCREEN =~ /$CURRENT_CONSENSUS/)   {
                        $CONSENSUS_FOUND = 2 ;
                        my $INFO_TO_UPDATE = $HASH_COUNT{$COUNT_ARRAY} ;
```

```
                        $INFO_TO_UPDATE = $INFO_TO_UPDATE + 1 ;
                        $HASH_COUNT{$COUNT_ARRAY} = $INFO_TO_UPDATE ;


                        my $FIVE_PRIME_BOUNDARY =  0 ; my $THREE_PRIME_BOUNDARY = 0 ;
                        my $DOWNSTREAM_LENGTH = 0 ; my $THREE_PRIME_FOUND =  0 ;
                        my $LENGTH_TO_SCREEN = length($CURRENT_CONSENSUS) ;
                        while ($THREE_PRIME_FOUND < 1)        {
                                my $DOWNSTREAM_SEARCH = substr($DOWNSTREAM_REGION_TO_SCREEN, 0,
$LENGTH_TO_SCREEN) ;
                                if ($DOWNSTREAM_SEARCH !~ /$CURRENT_CONSENSUS/)      {
                                        $LENGTH_TO_SCREEN = $LENGTH_TO_SCREEN + 1 ;

                        }
                                if ($DOWNSTREAM_SEARCH =~ /$CURRENT_CONSENSUS/)      {
                                        $FIVE_PRIME_BOUNDARY = $LENGTH_TO_SCREEN -
$LENGTH_CONSENSUS;
                                        $THREE_PRIME_BOUNDARY = $LENGTH_TO_SCREEN ;
                                        $DOWNSTREAM_LENGTH = $LENGTH_DOWNSTREAM_TO_SCREEN -
$THREE_PRIME_BOUNDARY ;
                                        $THREE_PRIME_FOUND =  2 ;

                        }

                                                                                }
                        my $FIVE_PRIME_REGION = substr($SEQ, 0, $LENGTH_FIVE_PRIME) ;
                        my $CONSERVED_REGION =
substr($DOWNSTREAM_REGION_TO_SCREEN,$FIVE_PRIME_BOUNDARY, $LENGTH_CONSENSUS) ;
#                       my $LENGTH_V3_NOT_ALIGNED = $FIVE_PRIME_BOUNDARY - $LENGTH_FIVE_PRIME ;
                        my $LENGTH_V3_NOT_ALIGNED = $FIVE_PRIME_BOUNDARY ;
                        my $V3 = substr($DOWNSTREAM_REGION_TO_SCREEN,0, $LENGTH_V3_NOT_ALIGNED);
                        my $NUMBER_OF_DASHES = $V3_TOTAL_LENGTH - $LENGTH_V3_NOT_ALIGNED ;
                        my $DASHES_COUNT = 0 ; my $V3_DASHES = "" ;
                        while ($DASHES_COUNT < $NUMBER_OF_DASHES)    {
                                $V3_DASHES = $V3_DASHES.="-" ;
                                $DASHES_COUNT = $DASHES_COUNT + 1 ;

        }
                        my $DOWNSTREAM = substr($DOWNSTREAM_REGION_TO_SCREEN,$THREE_PRIME_BOUNDARY
,$DOWNSTREAM_LENGTH) ;
                        my $ALIGNED_SEQ = "$FIVE_PRIME_REGION-$V3_DASHES-$V3-$CONSERVED_REGION-
$DOWNSTREAM"  ;
#                       $OUTPUT_FOUND = $OUTPUT_FOUND.="$LINE\n" ;
                        $OUTPUT_FOUND = $OUTPUT_FOUND.="$NAME\t$ALIGNED_SEQ\n" ;

                                }
                $COUNT_ARRAY = $COUNT_ARRAY + 1 ;

                                        }

        if ($CONSENSUS_FOUND < 1)      {
#               $COUNT_NOT_FOUND = $COUNT_NOT_FOUND + 1 ;
                $OUTPUT_NOT_FOUND = $OUTPUT_NOT_FOUND."$LINE\n" ;
                                                                }
                                }
close (PHYLIP);
#####################################
print "Preparing frequency output\n" ;
$COUNT_OUTPUT = 0;
while ($COUNT_OUTPUT < $NUMBER_CONSENSUS)    {
        my $CURRENT_CONSENSUS = $ARRAY_LIST[$COUNT_OUTPUT] ;
        my $FREQUENCY = $HASH_COUNT{$COUNT_OUTPUT} ;
        $OUTPUT_SUMMARY = $OUTPUT_SUMMARY.="Frequency of $CURRENT_CONSENSUS:\t$FREQUENCY\n" ;
        $COUNT_OUTPUT = $COUNT_OUTPUT + 1 ;
                                                                                }



open (OUTPUT, ">OUTPUT_C4_position_adjusted_$INPUT_FILE_NAME") or die "Can't open $file for
reading" ;
#print OUTPUT "Consensus and variants are:\n$CONSENSUS_VARIANT_LIST\n";
```

```
print OUTPUT "$OUTPUT_SUMMARY" ;
print OUTPUT "$OUTPUT_FOUND";
close (OUTPUT);

open (OUTPUT_2, ">OUTPUT_C4_position_NOT_FOUND_$INPUT_FILE_NAME") or die "Can't open $file for
reading" ;
print OUTPUT_2 "$OUTPUT_NOT_FOUND";
close (OUTPUT_2);
```

B9.16S_C4_trimming_ends

```
$FILE_NAME = "Input_file_name.phylip" ;
open (PHYLIP_FILE, "$FILE_NAME") or die "Can't open $file for reading" ;

$SEQUENCE_TO_FIND = "..-.........-.................TAA" ;
$LENGTH_SEQUENCE_TO_FIND = length($SEQUENCE_TO_FIND) ;

print "Screening for nt sequence\n" ;
$READ_COUNT = 0 ;   $OUTPUT_MATCHED = "" ; $OUTPUT_NOT_MATCHED = "" ; $OUTPUT_TRIMMED = "" ;

while (<PHYLIP_FILE>)  {
        $READ_COUNT = $READ_COUNT + 1 ; my $COUNT_THOUSAND = $READ_COUNT/5000 ;
        my $COUNT_INTEGER = int($COUNT_THOUSAND) ; my $INTEGER_CHECK = $COUNT_THOUSAND -
$COUNT_INTEGER;
        if ($INTEGER_CHECK == 0) {print "Processing sequence $READ_COUNT\n";        }

        my $LINE = $_; chomp ($LINE); my @ARRAY_LINE = split('\t',$LINE);
        my $NAME = $ARRAY_LINE[0] ; my $SEQUENCE = $ARRAY_LINE[1] ;

        my $LENGTH_SEQUENCE = length($SEQUENCE) ;
        my $START_OF_END_TO_CHECK = $LENGTH_SEQUENCE - $LENGTH_SEQUENCE_TO_FIND ;
        my $END_TO_CHECK = substr($SEQUENCE,$START_OF_END_TO_CHECK,$LENGTH_SEQUENCE_TO_FIND) ;

        if ($END_TO_CHECK =~ /$SEQUENCE_TO_FIND/)     {
                $OUTPUT_MATCHED = $OUTPUT_MATCHED.="$NAME\t$SEQUENCE\n" ;
                                                                                        }

        if ($END_TO_CHECK !~ /$SEQUENCE_TO_FIND/)     {
#               my @ARRAY_NT = split('',$SEQUENCE) ;
                my $SEQUENCE_TO_TRIM_FOUND = 0 ;
                my $START_POSITION_TRIM = $LENGTH_SEQUENCE - $LENGTH_SEQUENCE_TO_FIND ;
                while ($SEQUENCE_TO_TRIM_FOUND < 1 and $START_POSITION_TRIM > 0)    {
                        my $END_TO_TRIM =
substr($SEQUENCE,$START_POSITION_TRIM,$LENGTH_SEQUENCE_TO_FIND) ;
                        my @ARRAY_END_TO_TRIM = split('',$END_TO_TRIM) ;
                        my $COUNTER_END_TO_TRIM_CHECK = 0 ; my $NUMBER_DASHES = 0 ;
                        while ($COUNTER_END_TO_TRIM_CHECK < $LENGTH_SEQUENCE_TO_FIND)       {
                                my $CURRENT_CHARACTER =
$ARRAY_END_TO_TRIM[$COUNTER_END_TO_TRIM_CHECK] ;
                                if ($CURRENT_CHARACTER =~ /\-/) {$NUMBER_DASHES = $NUMBER_DASHES +
1 ;}
                                $COUNTER_END_TO_TRIM_CHECK = $COUNTER_END_TO_TRIM_CHECK + 1 ;

                                                }
                        if ($NUMBER_DASHES < 3) {
                                if ($END_TO_TRIM =~ /$SEQUENCE_TO_FIND/) {
                                my $LENGTH_TRIMMED = $START_POSITION_TRIM +
$LENGTH_SEQUENCE_TO_FIND;
                                my $TRIMMED_SEQ_FOR_OUTPUT = substr($SEQUENCE,0,$LENGTH_TRIMMED) ;
                                $OUTPUT_TRIMMED =
$OUTPUT_TRIMMED.="$NAME\t$TRIMMED_SEQ_FOR_OUTPUT\n" ;
                                $SEQUENCE_TO_TRIM_FOUND = 2 ;
                                                                                        }
                                                                        }
                        $START_POSITION_TRIM = $START_POSITION_TRIM - 1 ;

                                                }
                if ($SEQUENCE_TO_TRIM_FOUND < 1)      {
                        $OUTPUT_NOT_MATCHED = $OUTPUT_NOT_MATCHED.="$NAME\t$SEQUENCE\n" ;
                                                                                }
                                                                                        }
                                                        }
close (PHYLIP_FILE);


print "Writing to output file" ;

open (OUTPUT_1, ">OUTPUT_seqs_matched_to_query_$FILE_NAME") or die "Can't open $file for reading"
;
print OUTPUT_1 "$OUTPUT_MATCHED\n" ;
```

```
print OUTPUT_1 "$OUTPUT_TRIMMED" ;
close (OUTPUT_1);

open (OUTPUT_2, ">OUTPUT_seqs_NOT_matched_to_query_$FILE_NAME") or die "Can't open $file for
reading" ;
print OUTPUT_2 "$OUTPUT_NOT_MATCHED" ;
close (OUTPUT_2);
```

B10.16S_alignment_length_adjustment

```
$INPUT_FILE_NAME = "Input_file_name.phylip" ;
open (PHYLIP, "$INPUT_FILE_NAME") or die "Can't open $file for reading" ;

$EXPECTED_TOTAL_LENGTH = 900  ;
$POSITION_TO_INSERT_DASHES = 710 ;
$OUTPUT_TOO_LONG = "" ; $OUTPUT_OK = "" ; $OUTPUT_ADJUSTED = "" ;
$NUMBER_OK = 0 ; $NUMBER_ADJUSTED = 0 ;
while (<PHYLIP>) {
	$READ_COUNT = $READ_COUNT + 1 ; my $COUNT_THOUSAND = $READ_COUNT/2000 ;
	my $COUNT_INTEGER = int($COUNT_THOUSAND) ; my $INTEGER_CHECK = $COUNT_THOUSAND - 
$COUNT_INTEGER;
	if ($INTEGER_CHECK == 0) {print "Checking sequence $READ_COUNT\n";  }

	my $LINE = $_ ;		chomp ($LINE);
	if ($LINE =~ />/) {
		my @ARRAY_LINE = split('\t',$LINE) ;
		my $NAME = $ARRAY_LINE[0] ;   my $SEQ = $ARRAY_LINE[1] ; my $LENGTH_SEQ = 
length($SEQ) ;

		if ($LENGTH_SEQ == $EXPECTED_TOTAL_LENGTH)   {
			$OUTPUT_OK = $OUTPUT_OK.="$NAME\t$SEQ\n" ;
			$NUMBER_OK = $NUMBER_OK + 1 ;

	}
		if ($LENGTH_SEQ < $EXPECTED_TOTAL_LENGTH)    {
			my $UPSTREAM = substr($SEQ,0,$POSITION_TO_INSERT_DASHES) ;
			my $LENGTH_DOWNSTREAM = $LENGTH_SEQ - $POSITION_TO_INSERT_DASHES ;
			my $DOWNSTREAM = 
substr($SEQ,$POSITION_TO_INSERT_DASHES,$LENGTH_DOWNSTREAM) ;
			my $DASHES_TO_ADD = $EXPECTED_TOTAL_LENGTH - $LENGTH_SEQ ;
			my $DASHES = "" ;
			my $COUNT_ADDED_DASHES = 0 ;
			while ($COUNT_ADDED_DASHES < $DASHES_TO_ADD)        {
				$DASHES = $DASHES.="-" ;
				$COUNT_ADDED_DASHES = $COUNT_ADDED_DASHES + 1 ;

		}
			my $SEQ_ADJUSTED = "$UPSTREAM$DASHES$DOWNSTREAM" ;
			$OUTPUT_ADJUSTED = $OUTPUT_ADJUSTED.="$NAME\t$SEQ_ADJUSTED\n" ;
			$NUMBER_ADJUSTED = $NUMBER_ADJUSTED + 1 ;

	}

		if ($LENGTH_SEQ > $EXPECTED_TOTAL_LENGTH)    {
			$OUTPUT_TOO_LONG = $OUTPUT_TOO_LONG.="$NAME\t$SEQ\n" ;

	}
					}
				}
close (PHYLIP);

open (OUTPUT, ">OUTPUT_adjusted_length_$INPUT_FILE_NAME") or die "Can't open $file for reading" ;
print OUTPUT "$OUTPUT_OK\n" ;
print OUTPUT "$OUTPUT_ADJUSTED\n" ;
close (OUTPUT);

open (OUTPUT_SUMMARY, ">OUTPUT_summary_adjusted_length_$INPUT_FILE_NAME") or die "Can't open 
$file for reading" ;
print OUTPUT_SUMMARY "Sequences not needing length adjustment:\t$NUMBER_OK\n" ;
print OUTPUT_SUMMARY "Sequences with adjusted length:\t$NUMBER_ADJUSTED\n" ;
print OUTPUT_SUMMARY "Sequences that are longer than expected:\n" ;
print OUTPUT_SUMMARY "$OUTPUT_TOO_LONG\n" ;
close (OUTPUT_SUMMARY)  ;
```

C.1 16S_OTU_clustering_tool_using_5prime_to_C4_sorted_phylip

```
$INPUT_PHYLIP_NAME = " Input_file_name.phylip" ;

print "How many OTUs to cluster for:\n" ;
$NUMBER_OF_OTUS = <STDIN>; chomp($NUMBER_OF_OTUS) ;
print "What number would you like to assign to this round of clustering:\n" ;
$OTU_CLUSTERING_ROUND = <STDIN>; chomp($OTU_CLUSTERING_ROUND) ;

$INPUT_CUTOFF = 0.04 ;                #######PICK CORRECT CUTOFF
#$INPUT_CUTOFF = 0.05 ;                      #######PICK CORRECT CUTOFF
$CENTER_CUTOFF = $INPUT_CUTOFF/2 ;
$CLUSTER_CYCLE_UPDATE_INCREMENT = 500 ;

%PHYLIP_HASH ; @ARRAY_NAMES_TO_CLUSTER ;

$READ_COUNT = 0 ; $OUTPUT = "" ; $OUTPUT_COUNTS = "" ;
$OUTPUT_UNCLUSTERED = "" ; $OUTPUT_TIME_LOG = "" ;
print "Loading sequences in hash\n" ;
open (PHYLIP, "$INPUT_PHYLIP_NAME") or die "Can't open $file for reading" ;
while (<PHYLIP>)       {
       $READ_COUNT = $READ_COUNT + 1 ; my $COUNT_THOUSAND = $READ_COUNT/2000 ;
       my $COUNT_INTEGER = int($COUNT_THOUSAND) ; my $INTEGER_CHECK = $COUNT_THOUSAND -
$COUNT_INTEGER;
       if ($INTEGER_CHECK == 0) {print "Loading sequence $READ_COUNT\n";   }

       my $PHYLIP_LINE = $_ ; chomp ($PHYLIP_LINE) ;
       if ($PHYLIP_LINE =~ />/) {
              my @ARRAY_PHYLIP_LINE = split('\t', $PHYLIP_LINE);
              my $NAME = $ARRAY_PHYLIP_LINE[0] ; my $SEQ = $ARRAY_PHYLIP_LINE[1] ;
#CHECK LENGTH OF SEQUENCES
              $PHYLIP_HASH{$NAME} = $SEQ ;
       push (@ARRAY_NAMES_TO_CLUSTER,$NAME) ;
                                                  }
                                    }
close (PHYLIP);

#@ARRAY_NAMES_TO_CLUSTER = split('\t', $NAMES_LIST_COUNT_ORDERED) ;
$SIZE_ARRAY_NAMES_TO_CLUSTER = scalar(@ARRAY_NAMES_TO_CLUSTER) ;
$OTU_COUNT = 0 ;
print "Clustering $SIZE_ARRAY_NAMES_TO_CLUSTER sequence reads\n" ;
while ($OTU_COUNT < $NUMBER_OF_OTUS)  {
       print "Reads remaining to cluster: $SIZE_ARRAY_NAMES_TO_CLUSTER\n" ;
       $OTU_COUNT = $OTU_COUNT + 1 ;
       print "Clustering for OTU from cycle $OTU_COUNT\n" ;
       $OUTPUT = $OUTPUT.="\nOTU from cycle $OTU_COUNT\n";
       my $NAME_CENTER_OF_CLUSTER = $ARRAY_NAMES_TO_CLUSTER[0] ;
       $OUTPUT = $OUTPUT.="$NAME_CENTER_OF_CLUSTER," ;
       my $SEQ_CENTER_OF_CLUSTER = $PHYLIP_HASH{$NAME_CENTER_OF_CLUSTER} ;
       my @ARRAY_NT_CENTER_OF_CLUSTER = split('', $SEQ_CENTER_OF_CLUSTER) ;
       my $LENGTH_SEQ = length($SEQ_CENTER_OF_CLUSTER) ;
       my $UNMATCHED_NAMES = "" ;
       my $COUNT_ARRAY_CLUSTER = 1 ;
       while ($COUNT_ARRAY_CLUSTER < $SIZE_ARRAY_NAMES_TO_CLUSTER) {
              my $NAME_NEXT_SEQ = $ARRAY_NAMES_TO_CLUSTER[$COUNT_ARRAY_CLUSTER] ;
              my $NEXT_SEQ = $PHYLIP_HASH{$NAME_NEXT_SEQ} ;
              my @ARRAY_NT_NEXT_SEQ = split('', $NEXT_SEQ) ;
              my $NT_POSITION = 0 ; my $DISSIMILARITY_COUNT = 0 ;
              my $PERCENT_DISSIMILARITY = 0 ;
              while ($NT_POSITION < $LENGTH_SEQ and $PERCENT_DISSIMILARITY <= $CENTER_CUTOFF)
       {
                     my $CURRENT_NT_CENTER = $ARRAY_NT_CENTER_OF_CLUSTER[$NT_POSITION] ;
                     my $CURRENT_NT_NEXT_SEQ       = $ARRAY_NT_NEXT_SEQ[$NT_POSITION] ;
                     if      ($CURRENT_NT_CENTER ne $CURRENT_NT_NEXT_SEQ) {
                            $DISSIMILARITY_COUNT= $DISSIMILARITY_COUNT + 1 ;
                            $PERCENT_DISSIMILARITY = $DISSIMILARITY_COUNT/$LENGTH_SEQ ;

                     }
                     $NT_POSITION = $NT_POSITION + 1;

                                                                           }
```

```
#               $PERCENT_DISSIMILARITY = $DISSIMILARITY_COUNT/$LENGTH_SEQ ;
                if ($PERCENT_DISSIMILARITY <= $CENTER_CUTOFF)        {
                        $OUTPUT = $OUTPUT.="$NAME_NEXT_SEQ," ;

        }
                if ($PERCENT_DISSIMILARITY > $CENTER_CUTOFF)         {
                        $UNMATCHED_NAMES = $UNMATCHED_NAMES.="$NAME_NEXT_SEQ\t" ;

        }
                $COUNT_ARRAY_CLUSTER = $COUNT_ARRAY_CLUSTER + 1;

                        }
        chop ($OUTPUT) ;



        #################### TEST FOR PROGRESS OUTPUT ####
        my $CLUSTERING_PROGRESS_TALLY = $OTU_COUNT/$CLUSTER_CYCLE_UPDATE_INCREMENT ;
        my $CLUSTERING_PROGRESS_TALLY_INTEGER = int($CLUSTERING_PROGRESS_TALLY) ;
        my $CLUSTERING_PROGRESS_TALLY_INTEGER_CHECK = $CLUSTERING_PROGRESS_TALLY -
$CLUSTERING_PROGRESS_TALLY_INTEGER;

        if ($CLUSTERING_PROGRESS_TALLY_INTEGER_CHECK == 0)   {
                print "Writing to progress file\n" ;
                open (OUTPUT_PROGRESS, ">OUTPUT $INPUT_PHYLIP_NAME\_progress_round
$OTU_COUNT.list") or die "Can't open $file for reading" ;
                print OUTPUT_PROGRESS "$OUTPUT" ;
                close (OUTPUT_PROGRESS);                                    }

        ################      END TEST FOR PROGRESS OUTPUT ####

        @ARRAY_NAMES_TO_CLUSTER = split('\t', $UNMATCHED_NAMES) ;
        $SIZE_ARRAY_NAMES_TO_CLUSTER = scalar(@ARRAY_NAMES_TO_CLUSTER) ;
        my @timeData = localtime(time) ;
        my $YEAR = $timeData[5] + 1900 ;
        my $MONTH = $timeData[4] + 1 ;
        my $DATE_TIME = "$MONTH-$timeData[3]-$YEAR\t$timeData[2]:$timeData[1]";
        $OUTPUT_TIME_LOG = $OUTPUT_TIME_LOG.="OTU $OTU_COUNT done at $DATE_TIME\n" ;
                                                                                }

print "Generating counts information\n" ;
@ARRAY_OUTPUT = split('\n',$OUTPUT) ;
$NUMBER_OTU_LINES = scalar(@ARRAY_OUTPUT) ;
$COUNTS_FOR_COUNTS = 0 ;
while ($COUNTS_FOR_COUNTS < $NUMBER_OTU_LINES)      {
        my $CURRENT_OUTPUT_LINE = $ARRAY_OUTPUT[$COUNTS_FOR_COUNTS] ;
        if ($CURRENT_OUTPUT_LINE !~ />/)                    {
                $OUTPUT_COUNTS = $OUTPUT_COUNTS.="$CURRENT_OUTPUT_LINE\t" ;
                                                                                        }
        if ($CURRENT_OUTPUT_LINE =~ />/)                    {
                my @ARRAY_CURRENT_OUTPUT_LINE = split(',', $CURRENT_OUTPUT_LINE) ;
                my $OTU_COUNT = scalar(@ARRAY_CURRENT_OUTPUT_LINE) ;
                my $OTU_REP = $ARRAY_CURRENT_OUTPUT_LINE[0] ;
                $OUTPUT_COUNTS = $OUTPUT_COUNTS.="$OTU_REP\t$OTU_COUNT\n" ;
                                                                                        }
        $COUNTS_FOR_COUNTS = $COUNTS_FOR_COUNTS + 1 ;
                                                                                        }
print "Creating phylip for unclustered sequences\n" ;
$COUNT_UNCLUSTERED = 0 ;
while ($COUNT_UNCLUSTERED < $SIZE_ARRAY_NAMES_TO_CLUSTER)   {
        my $CURRENT_UNCLUSTERED = $ARRAY_NAMES_TO_CLUSTER[$COUNT_UNCLUSTERED] ;
        my $SEQ_UNCLUSTERED = $PHYLIP_HASH{$CURRENT_UNCLUSTERED} ;
        $OUTPUT_UNCLUSTERED = $OUTPUT_UNCLUSTERED.="$CURRENT_UNCLUSTERED\t$SEQ_UNCLUSTERED\n" ;
        $COUNT_UNCLUSTERED = $COUNT_UNCLUSTERED + 1 ;

                }

open (OUTPUT,
">OUTPUT_$INPUT_PHYLIP_NAME\_cluster_$INPUT_CUTOFF\_round_$OTU_CLUSTERING_ROUND.list") or die
"Can't open $file for reading" ;
print OUTPUT "$OUTPUT";
```

```
close (OUTPUT);
open (OUTPUT2,
">OUTPUT_$INPUT_PHYLIP_NAME\_cluster_$INPUT_CUTOFF\_round_$OTU_CLUSTERING_ROUND.count") or die
"Can't open $file for reading" ;
print OUTPUT2 "$OUTPUT_COUNTS";
close (OUTPUT2);
open (OUTPUT3,
">OUTPUT_$INPUT_PHYLIP_NAME\_unclustered_reads\_round_$OTU_CLUSTERING_ROUND.phylip") or die
"Can't open $file for reading" ;
print OUTPUT3 "$OUTPUT_UNCLUSTERED";
close (OUTPUT3);
open (OUTPUT4,
">OUTPUT_$INPUT_PHYLIP_NAME\_cluster_$INPUT_CUTOFF\_time_log\_round_$OTU_CLUSTERING_ROUND.txt")
or die "Can't open $file for reading" ;
print OUTPUT4 "$OUTPUT_TIME_LOG";
close (OUTPUT4);
```

C.2 16S_OTU_List_transform_clustering_list_into_format1_and_format2

```
$INPUT_FILE_LIST = " Input_file_name.list" ;

@ARRAY_FILE_NAME = split('\.',$INPUT_FILE_LIST) ;
$FILE_NAME_NO_EXTENSION = $ARRAY_FILE_NAME[0] ;

$OUTPUT_FORMAT1 = "" ; $OUTPUT_FORMAT2 = "" ;
print "Processing list data information\n" ;
open (LIST_FILE, "$INPUT_FILE_LIST") or die "Can't open $file for reading" ;
$READ_COUNT = 0 ;
while (<LIST_FILE>) {
        $READ_COUNT = $READ_COUNT + 1 ; my $COUNT_THOUSAND = $READ_COUNT/1000 ;
        my $COUNT_INTEGER = int($COUNT_THOUSAND) ; my $INTEGER_CHECK = $COUNT_THOUSAND -
$COUNT_INTEGER;
        if ($INTEGER_CHECK == 0) {print "Processing list $READ_COUNT\n";    }

        my $LINE = $_ ; chomp ($LINE) ;
        if ($LINE =~ />/)      {
                my @ARRAY_LINE = split(',',$LINE) ;
                my $OTU_REP = $ARRAY_LINE[0] ;
                $OUTPUT_FORMAT1 = $OUTPUT_FORMAT1.="$LINE\n" ;
                $OUTPUT_FORMAT2 = $OUTPUT_FORMAT2.="$OTU_REP\t$LINE\n" ;
                                                }
                                }
close (LIST_FILE) ;

print "Creating output\n" ;

open (OUTPUT1, ">OUTPUT_format1_$FILE_NAME_NO_EXTENSION\.list") or die "Can't open $file for
reading" ;
print OUTPUT1 "$OUTPUT_FORMAT1" ;
close (OUTPUT1);

open (OUTPUT2, ">OUTPUT_format2_$FILE_NAME_NO_EXTENSION\.list") or die "Can't open $file for
reading" ;
print OUTPUT2 "$OUTPUT_FORMAT2" ;
close (OUTPUT2);
```

C.3 16S_OTU_blast_16S_sorting_sequence_identities_to_alignments

```
$INPUT_FILE_BLASTX = " Input_file_name.txt" ;

open (BLASTX, "$INPUT_FILE_BLASTX") or die "Can't open $file for reading" ;
$QUERY_NAME_LIST = "" ;
#$OUTPUT = "QueryName\tTargetName\tIdenticals\tAlignLength\tPercentIdentity\n" ;
$OUTPUT = "QueryName\tTargetName\tAlignAllLength\tDissimilarnt\tPercentIdentity\n" ;
$READ_COUNT = 0 ;
print "Starting search\n" ;
while (<BLASTX>) {
	my $BLASTX_LINE = $_ ; chomp ($BLASTX_LINE) ;
	if ($BLASTX_LINE =~ /Query=/)	{
		$READ_COUNT = $READ_COUNT + 1 ; my $COUNT_THOUSAND = $READ_COUNT/1000 ;
		my $COUNT_INTEGER = int($COUNT_THOUSAND) ; my $INTEGER_CHECK = $COUNT_THOUSAND -
$COUNT_INTEGER;
		if ($INTEGER_CHECK == 0) {print "Processing info for read number $READ_COUNT\n";
	}

		my $QUERY_NAME_LINE = $BLASTX_LINE ;
		my @ARRAY_QUERY_NAME_LINE = split('= ',$QUERY_NAME_LINE) ;
		my $QUERY_NAME = $ARRAY_QUERY_NAME_LINE[1] ; $QUERY_NAME_LIST =
$QUERY_NAME_LIST.="$QUERY_NAME,";
#		print "Query name $QUERY_NAME\n" ;
		my $EMPTY_LINE = <BLASTX> ;
		my $QUERY_LENGTH_LINE = <BLASTX> ; chomp($QUERY_LENGTH_LINE) ;
		my @ARRAY_QUERY_LENGTH_LINE = split('=',$QUERY_LENGTH_LINE) ;
		my $QUERY_LENGTH = $ARRAY_QUERY_LENGTH_LINE[1] ;

		my $INFO_CURRENT_QUERY = "$QUERY_NAME\t" ;
		my $TARGET_LENGTH = 0 ; my $ALIGNMENT_LENGTH = 0 ; my $NUMBER_IDENTICALS = 0 ; my
$PERCENT_IDENTICAL = 0.00 ;
		my $NUMBER_DISSIMILAR_NT = 1000 ;
		my $END_QUERY_INFO_REACHED = 0 ;
		my $FIRST_TARGET_FOUND = 0 ;
		while ($END_QUERY_INFO_REACHED < 1) {

			my $CURRENT_LINE = <BLASTX> ; chomp ($CURRENT_LINE) ;

			if ($CURRENT_LINE =~ />/)	{
				my $TARGET_NAME_LINE = $CURRENT_LINE ;
				my @ARRAY_TARGET_NAME_LINE = split(' ',$TARGET_NAME_LINE) ;
				my $TARGET_NAME = $ARRAY_TARGET_NAME_LINE[0] ;
				my $TARGET_LENGTH_LINE_FOUND = 0 ;
				while ($TARGET_LENGTH_LINE_FOUND < 1)	{
					my $NEXT_LINE = <BLASTX> ;  chomp ($NEXT_LINE) ;
					if ($NEXT_LINE =~ /Length=/)  { $TARGET_LENGTH_LINE_FOUND =
2 ;  }

	}
				my $ALIGNMENT_LENGTH_FOUND = 0 ;
				while ($ALIGNMENT_LENGTH_FOUND < 1) {
					my $NEXT_LINE = <BLASTX> ;  chomp ($NEXT_LINE) ;
					if ($NEXT_LINE =~ /Identities/) {
						$ALIGNMENT_LENGTH_FOUND = 2 ;
#						print "Alignment length found\n" ;
						my @ARRAY_IDENTITIES_LINE = split(',',$NEXT_LINE) ;
						my $IDENTITIES_ELEMENT = $ARRAY_IDENTITIES_LINE[0] ;
						my @ARRAY_IDENTITIES_ELEMENT = split('
',$IDENTITIES_ELEMENT) ;
						my $NUMBER_IDENTITIES_ELEMENTS =
scalar(@ARRAY_IDENTITIES_ELEMENT) ;
						my $COUNTER_IDENTITIES_ELEMENT = 0 ;
						while ($COUNTER_IDENTITIES_ELEMENT <
$NUMBER_IDENTITIES_ELEMENTS)  {
							my $IDENTITIES_ELEMENT_TO_TEST =
$ARRAY_IDENTITIES_ELEMENT[$COUNTER_IDENTITIES_ELEMENT] ;
							if ($IDENTITIES_ELEMENT_TO_TEST =~ /\//)
	{
								my @ARRAY_ALIGNMENT_LENGTH =
split('\/',$IDENTITIES_ELEMENT_TO_TEST) ;
```

```
                                                            $NUMBER_IDENTICALS =
$ARRAY_ALIGNMENT_LENGTH[0] ;
                                                            $ALIGNMENT_LENGTH =
       $ARRAY_ALIGNMENT_LENGTH[1] ;
                                                            $PERCENT_IDENTICAL =
$NUMBER_IDENTICALS/$ALIGNMENT_LENGTH ;
                                                            $NUMBER_DISSIMILAR_NT =
$ALIGNMENT_LENGTH - $NUMBER_IDENTICALS ;
                                                            my $ALIGN_LENGTH_TEST = "ND" ;
                                                            if ($FIRST_TARGET_FOUND < 1)  {
                                                                   $FIRST_TARGET_FOUND = 2 ;
#                                                                  print "First target found\n"
;
                                                                   if ($QUERY_LENGTH ==
$ALIGNMENT_LENGTH) {$ALIGN_LENGTH_TEST = "Y" ;}
                                                                   if ($QUERY_LENGTH !=
$ALIGNMENT_LENGTH) {$ALIGN_LENGTH_TEST = "N" ;}
                                                                   $INFO_CURRENT_QUERY =
$INFO_CURRENT_QUERY.="$TARGET_NAME\t$ALIGN_LENGTH_TEST\t$NUMBER_DISSIMILAR_NT\t$PERCENT_IDENTICAL
" ;
#                                                                  $INFO_CURRENT_QUERY =
$INFO_CURRENT_QUERY.="$TARGET_NAME\t$NUMBER_IDENTICALS\t$ALIGNMENT_LENGTH\t$PERCENT_IDENTICAL" ;

#                                                                  print
"$TARGET_NAME\t$ALIGN_LENGTH_TEST\t$NUMBER_DISSIMILAR_NT\n" ;

                      }

                                     }
                                                     $COUNTER_IDENTITIES_ELEMENT =
$COUNTER_IDENTITIES_ELEMENT + 1 ;

                                                                          }


       }

       }
                                                                                  }
#                      if ($CURRENT_LINE =~ /Effective/)     {$END_QUERY_INFO_REACHED = 2 ; }
#NOTE: CAME ACROSS BLAST OUTPUT FILES THAT WERE MISSING EFFECTIVE AT THE END OF THE SEARCH FOR A
QUERY
                      if ($CURRENT_LINE =~ /Lambda/)        {$END_QUERY_INFO_REACHED = 2 ; }

                                                                                  }
               $OUTPUT = $OUTPUT.="$INFO_CURRENT_QUERY\n" ;
                                                                          }
                             }
close (BLASTX) ;



open (OUTPUT, ">OUTPUT_identities_sorted_$INPUT_FILE_BLASTX") or die "Can't open $file for
reading" ;
print OUTPUT "$OUTPUT" ;
close (OUTPUT);
```

C.4 16S_OTU_blast_processing_results_for_sorting_BAD_and_other_reads

```
$INPUT_FILE_BLASTX = " Input_file_name.txt" ;

open (BLASTX, "$INPUT_FILE_BLASTX") or die "Can't open $file for reading" ;
$QUERY_NAME_LIST = "" ; %HASH_BASIC_INFO ; %HASH_ALIGNMENT_SCORES ;
$BAD_5PRIME_LIST ; $BAD_3PRIME_LIST ; $UNBALANCED_LIST ;
$GOOD_LIST ;
$READ_COUNT = 0 ;
while (<BLASTX>) {
	my $BLASTX_LINE = $_ ; chomp ($BLASTX_LINE) ;
	if ($BLASTX_LINE =~ /Query=/)	{
		$READ_COUNT = $READ_COUNT + 1 ; my $COUNT_THOUSAND = $READ_COUNT/100 ;
		my $COUNT_INTEGER = int($COUNT_THOUSAND) ; my $INTEGER_CHECK = $COUNT_THOUSAND - 
$COUNT_INTEGER;
		if ($INTEGER_CHECK == 0) {print "Processing info for read number $READ_COUNT\n";
	}
#retrieving query info
		my $QUERY_NAME_LINE = $BLASTX_LINE ;
		my @ARRAY_QUERY_NAME_LINE = split('= ',$QUERY_NAME_LINE) ;
		my $QUERY_NAME = $ARRAY_QUERY_NAME_LINE[1] ; $QUERY_NAME_LIST = 
$QUERY_NAME_LIST.="$QUERY_NAME,";
		my $EMPTY_LINE = <BLASTX> ;
		my $QUERY_LENGTH_LINE = <BLASTX> ; chomp($QUERY_LENGTH_LINE) ;
		my @ARRAY_QUERY_LENGTH_LINE = split('=',$QUERY_LENGTH_LINE) ;
		my $QUERY_LENGTH = $ARRAY_QUERY_LENGTH_LINE[1] ;

		my $INFO_CURRENT_QUERY = "$QUERY_NAME\t$QUERY_LENGTH\t" ;
#initializing variables for align info to retrieve
		my $NUMBER_ALIGNMENTS_FOUND = 0 ;
		my $TARGET_LENGTH = 0 ; my $ALIGNMENT_LENGTH = 0 ;
		my $QUERY_ALIGN_START = 0 ; my $TARGET_ALIGN_START = 0 ;
		my $QUERY_ALIGN_END = 0 ; my $TARGET_ALIGN_END = 0 ;
		my $ALIGNMENT_SCORES = "" ;
		my $END_QUERY_INFO_REACHED = 0 ;
		while ($END_QUERY_INFO_REACHED < 1) {
			my $CURRENT_LINE = <BLASTX> ; chomp ($CURRENT_LINE) ;

			if ($CURRENT_LINE =~ />/ and $NUMBER_ALIGNMENTS_FOUND < 1)	{
				$NUMBER_ALIGNMENTS_FOUND = 2 ;
				my $TARGET_LENGTH_LINE_FOUND = 0 ;
				while ($TARGET_LENGTH_LINE_FOUND < 1)	{
					my $NEXT_LINE = <BLASTX> ;  chomp ($NEXT_LINE) ;
					if ($NEXT_LINE =~ /Length=/)	{
						$TARGET_LENGTH_LINE_FOUND = 2 ;
						my @ARRAY_TARGET_LENGTH_LINE = split('=',$NEXT_LINE) 
;
						$TARGET_LENGTH = $ARRAY_TARGET_LENGTH_LINE[1] ;
						$INFO_CURRENT_QUERY = 
$INFO_CURRENT_QUERY.="$TARGET_LENGTH\t" ;

	}

	}
				my $ALIGNMENT_LENGTH_FOUND = 0 ;
				while ($ALIGNMENT_LENGTH_FOUND < 1) {
					my $NEXT_LINE = <BLASTX> ;  chomp ($NEXT_LINE) ;
					if ($NEXT_LINE =~ /Identities/) {
						$ALIGNMENT_LENGTH_FOUND = 2 ;
						my @ARRAY_IDENTITIES_LINE = split(',',$NEXT_LINE) ;
						my $IDENTITIES_ELEMENT = $ARRAY_IDENTITIES_LINE[0] ;
						my @ARRAY_IDENTITIES_ELEMENT = split('
',$IDENTITIES_ELEMENT) ;
						my $NUMBER_IDENTITIES_ELEMENTS = 
scalar(@ARRAY_IDENTITIES_ELEMENT) ;
						my $COUNTER_IDENTITIES_ELEMENT = 0 ;
						while ($COUNTER_IDENTITIES_ELEMENT < 
$NUMBER_IDENTITIES_ELEMENTS)  {
							my $IDENTITIES_ELEMENT_TO_TEST = 
$ARRAY_IDENTITIES_ELEMENT[$COUNTER_IDENTITIES_ELEMENT] ;
```

```
                                                if ($IDENTITIES_ELEMENT_TO_TEST =~ /\//)
        {
                                                        my @ARRAY_ALIGNMENT_LENGTH =
split('\/',$IDENTITIES_ELEMENT_TO_TEST) ;
                                                        $ALIGNMENT_LENGTH =
        $ARRAY_ALIGNMENT_LENGTH[1] ;
                                                        $INFO_CURRENT_QUERY =
$INFO_CURRENT_QUERY.="$ALIGNMENT_LENGTH\t" ;
                        }
                                                $COUNTER_IDENTITIES_ELEMENT =
$COUNTER_IDENTITIES_ELEMENT + 1 ;

                                                                        }


        }

        }

                        my $ALIGNMENT_LINE_NUMBER_RAW = $ALIGNMENT_LENGTH/60 ;
                        my $ALIGNMENT_LINE_NUMBER_INT = int($ALIGNMENT_LINE_NUMBER_RAW) ;
                        my $ALIGNMENT_LINE_NUMBER_TEST = $ALIGNMENT_LINE_NUMBER_RAW -
$ALIGNMENT_LINE_NUMBER_INT ;
                        my $NUMBER_ALIGN_LINES = 0 ; my $LENGTH_LAST_ALIGN_LINE = 0 ;
                        if ($ALIGNMENT_LINE_NUMBER_TEST == 0)        {
                                $NUMBER_ALIGN_LINES = $ALIGNMENT_LINE_NUMBER_INT ;
                                $LENGTH_LAST_ALIGN_LINE = 60 ;

        }
                        if ($ALIGNMENT_LINE_NUMBER_TEST > 0)  {
                                $NUMBER_ALIGN_LINES = $ALIGNMENT_LINE_NUMBER_INT + 1 ;
                                my $CUMULATIVE_LENGTH_FULL_LINES =
$ALIGNMENT_LINE_NUMBER_INT * 60 ;
                                $LENGTH_LAST_ALIGN_LINE = $ALIGNMENT_LENGTH -
$CUMULATIVE_LENGTH_FULL_LINES ;

        }
                        $ALIGNMENT_SCORES = $ALIGNMENT_SCORES.="$NUMBER_ALIGN_LINES\t" ;

                        my $END_OF_ALIGNMENT_REACHED = 0 ; my $COUNTER_QUERY_ALIGN = 0 ;
                        while ($END_OF_ALIGNMENT_REACHED < 1)        {
                                my $NEXT_LINE = <BLASTX> ;  chomp ($NEXT_LINE) ;
                                if ($NEXT_LINE =~ /Query/ and $NEXT_LINE !~ /=/)     {
                                        my $ALIGN_QUERY_LINE = $NEXT_LINE ;
                                        $COUNTER_QUERY_ALIGN = $COUNTER_QUERY_ALIGN + 1 ;
                                        if ($COUNTER_QUERY_ALIGN == 1)       {
                                                my @ARRAY_QUERY_LINE = split('
',$ALIGN_QUERY_LINE) ;
                                                $QUERY_ALIGN_START = $ARRAY_QUERY_LINE[1] ;
                                                $INFO_CURRENT_QUERY =
$INFO_CURRENT_QUERY.="$QUERY_ALIGN_START\t" ;

        }
                                        if ($COUNTER_QUERY_ALIGN == $NUMBER_ALIGN_LINES)
        {
                                                my @ARRAY_QUERY_LINE = split('
',$ALIGN_QUERY_LINE) ;
                                                $QUERY_ALIGN_END = $ARRAY_QUERY_LINE[3] ;
                                                $INFO_CURRENT_QUERY =
$INFO_CURRENT_QUERY.="$QUERY_ALIGN_END\t" ;

                                                }


                                        my $ALIGN_BARS_LINE = <BLASTX> ;
chomp($ALIGN_BARS_LINE) ;
                                        my $NUMBER_ALIGN_BARS = $ALIGN_BARS_LINE =~ tr/\|//
;
                                        if ($COUNTER_QUERY_ALIGN < $NUMBER_ALIGN_LINES)
        {
```

```
                                                    my $CURRENT_ALIGNMENT_SCORE =
$NUMBER_ALIGN_BARS/60 ;
                                                    my $ALIGNMENT_SCORE_ROUNDED =
sprintf("%0.2f",$CURRENT_ALIGNMENT_SCORE) ;
                                                    $ALIGNMENT_SCORES =
$ALIGNMENT_SCORES."$ALIGNMENT_SCORE_ROUNDED," ;

                                             }
                                             if ($COUNTER_QUERY_ALIGN == $NUMBER_ALIGN_LINES)
        {
                                                    my $CURRENT_ALIGNMENT_SCORE =
$NUMBER_ALIGN_BARS/$LENGTH_LAST_ALIGN_LINE ;
                                                    my $ALIGNMENT_SCORE_ROUNDED =
sprintf("%0.2f",$CURRENT_ALIGNMENT_SCORE) ;
                                                    $ALIGNMENT_SCORES =
$ALIGNMENT_SCORES."$ALIGNMENT_SCORE_ROUNDED," ;

                                             }
                                             #$INFO_CURRENT_QUERY =
$INFO_CURRENT_QUERY.="$NUMBER_ALIGN_BARS\t" ;

                                             my $ALIGN_TARGET_LINE = <BLASTX> ;
chomp($ALIGN_TARGET_LINE) ;
                                             if ($COUNTER_QUERY_ALIGN == 1)        {
                                                    my @ARRAY_TARGET_LINE = split('
',$ALIGN_TARGET_LINE) ;
                                                    $TARGET_ALIGN_START = $ARRAY_TARGET_LINE[1]
;
                                                    $INFO_CURRENT_QUERY =
$INFO_CURRENT_QUERY.="$TARGET_ALIGN_START\t" ;

        }
                                             if ($COUNTER_QUERY_ALIGN == $NUMBER_ALIGN_LINES)
        {
                                                    my @ARRAY_TARGET_LINE = split('
',$ALIGN_TARGET_LINE) ;
                                                    $TARGET_ALIGN_END = $ARRAY_TARGET_LINE[3] ;
                                                    $INFO_CURRENT_QUERY =
$INFO_CURRENT_QUERY.="$TARGET_ALIGN_END\t" ;

                                                    }


                                      }
#                                     if ($NEXT_LINE =~ />/) {$END_OF_ALIGNMENT_REACHED = 2 ;}
                                      if ($NEXT_LINE =~ /Lambda/) {$END_OF_ALIGNMENT_REACHED = 2
;}

        }

                                      }
                      if ($CURRENT_LINE =~ /Effective/)     {$END_QUERY_INFO_REACHED = 2 ; }


                                                                                  }
               $HASH_ALIGNMENT_SCORES{$QUERY_NAME} = $ALIGNMENT_SCORES ;
               $HASH_BASIC_INFO{$QUERY_NAME} = $INFO_CURRENT_QUERY ;
#              $OUTPUT = $OUTPUT.="$INFO_CURRENT_QUERY\n" ;
#checking if bad 5 prime
               if ($QUERY_ALIGN_START > 5 and $TARGET_ALIGN_START > 5) {
                              $BAD_5PRIME_LIST = $BAD_5PRIME_LIST.="$QUERY_NAME," ;

                      }
#checking for bad 3 prime
               my $QUERY_END_CHECK = $QUERY_LENGTH - $QUERY_ALIGN_END ;
               my $TARGET_END_CHECK = $TARGET_LENGTH - $TARGET_ALIGN_END ;
               if ($QUERY_END_CHECK > 5 and $TARGET_END_CHECK > 5) {
                      $BAD_3PRIME_LIST = $BAD_3PRIME_LIST.="$QUERY_NAME," ;

               }
```

```
                                                                        }
                                                                }
close (BLASTX) ;

print "Preparing output for bad 5 prime end\n" ;
$OUTPUT = $OUTPUT.="Reads with bad 5prime ends\n" ;
$OUTPUT =
$OUTPUT.="QueryName\tQueryLength\tTargetLength\tAlignLength\tQueryStart\tTargetStart\tQueryEnd\tT
argetEnd\n" ;
@ARRAY_BAD_5PRIME = split(',',$BAD_5PRIME_LIST)  ;
$NUMBER_BAD_5PRIME = scalar(@ARRAY_BAD_5PRIME) ;
$COUNTER_BAD_5PRIME = 0 ;
while ($COUNTER_BAD_5PRIME < $NUMBER_BAD_5PRIME)     {
        my $CURRENT_BAD_5PRIME = $ARRAY_BAD_5PRIME[$COUNTER_BAD_5PRIME] ;
        my $HASH_INFO_RETRIEVE = $HASH_BASIC_INFO{$CURRENT_BAD_5PRIME} ;
        $OUTPUT = $OUTPUT.="$HASH_INFO_RETRIEVE\n" ;
        $COUNTER_BAD_5PRIME = $COUNTER_BAD_5PRIME + 1 ;


        }

print "Preparing output for bad 3 prime end\n" ;
$OUTPUT = $OUTPUT.="Reads with bad 3prime ends\n" ;
$OUTPUT =
$OUTPUT.="QueryName\tQueryLength\tTargetLength\tAlignLength\tQueryStart\tTargetStart\tQueryEnd\tT
argetEnd\n" ;
@ARRAY_BAD_3PRIME = split(',',$BAD_3PRIME_LIST)  ;
$NUMBER_BAD_3PRIME = scalar(@ARRAY_BAD_3PRIME) ;
$COUNTER_BAD_3PRIME = 0 ;
while ($COUNTER_BAD_3PRIME < $NUMBER_BAD_3PRIME)     {
        my $CURRENT_BAD_3PRIME = $ARRAY_BAD_3PRIME[$COUNTER_BAD_3PRIME] ;
        my $HASH_INFO_RETRIEVE = $HASH_BASIC_INFO{$CURRENT_BAD_3PRIME} ;
        $OUTPUT = $OUTPUT.="$HASH_INFO_RETRIEVE\n" ;
        $COUNTER_BAD_3PRIME = $COUNTER_BAD_3PRIME + 1 ;


        }
print "Preparing output for other reads\n" ;
$OUTPUT = $OUTPUT.="Alignment scores for other reads\n" ;
$OUTPUT = $OUTPUT.="QueryName\tNumberAlignLines\n" ;
@ARRAY_QUERY_NAME_LIST = split(',',$QUERY_NAME_LIST) ;
$NUMBER_ALL_READS = scalar(@ARRAY_QUERY_NAME_LIST)   ;
$COUNTER_ALL_READS = 0 ;
while ($COUNTER_ALL_READS < $NUMBER_ALL_READS)       {
        my $CURRENT_READ_TO_OUTPUT = $ARRAY_QUERY_NAME_LIST[$COUNTER_ALL_READS] ;
        if ($BAD_5PRIME_LIST !~ /$CURRENT_READ_TO_OUTPUT/ and $BAD_3PRIME_LIST !~
/$CURRENT_READ_TO_OUTPUT/)    {
                my $ALIGNMENT_SCORES_TO_OUTPUT = $HASH_ALIGNMENT_SCORES{$CURRENT_READ_TO_OUTPUT} ;
                $OUTPUT = $OUTPUT.="$CURRENT_READ_TO_OUTPUT\t$ALIGNMENT_SCORES_TO_OUTPUT\n" ;


        }
        $COUNTER_ALL_READS = $COUNTER_ALL_READS + 1 ;
                                                                                     }

open (OUTPUT, ">OUTPUT_screening_for_bad_reads_$INPUT_FILE_BLASTX") or die "Can't open $file for
reading" ;
print OUTPUT "$OUTPUT" ;
close (OUTPUT);
```

C.5 16S_OTU_Identities_file_retrieving_lines_from_accnos_file_name_adjustment

```
$COUNT_FILE_NAME = " Input_file_name.txt" ;
$ACCNOS_FILE_NAME = "Bison Longitudinal Gradient_singletons-doubletons to check.accnos" ; ###

$OUTPUT = "" ;        $NAMES_NOT_FOUND = "Names without a matching line from input:\n" ;

%HASH_COUNT ; $NAME_COUNT = 0 ;
print "Loading count file into hash\n" ;
open (COUNT, "$COUNT_FILE_NAME") or die "Can't open $file for reading" ;
while (<COUNT>) {
	$NAME_COUNT = $NAME_COUNT + 1 ; my $COUNT_THOUSAND = $NAME_COUNT /5000 ;
	my $COUNT_INTEGER = int($COUNT_THOUSAND) ; my $INTEGER_CHECK = $COUNT_THOUSAND -
$COUNT_INTEGER;
	if ($INTEGER_CHECK == 0) {print "Processing name $NAME_COUNT\n";}

	my $LINE = $_ ; chomp($LINE) ; my @ARRAY_COUNT_LINE = split('\t', $LINE);
	my $UNIQUE_NAME = $ARRAY_COUNT_LINE[0] ;
	if ($UNIQUE_NAME !~ />/)      {
		$UNIQUE_NAME = ">$UNIQUE_NAME" ;
							}
	$HASH_COUNT{$UNIQUE_NAME} = $LINE ;
				}
close (COUNT) ;

print "Starting read count retrieval\n";
open (ACCNOS, "$ACCNOS_FILE_NAME") or die "Can't open $file for reading" ;
$LINE_COUNT = 0 ;
while (<ACCNOS>)      {
	$LINE_COUNT = $LINE_COUNT + 1 ; my $COUNT_THOUSAND = $LINE_COUNT /5000 ;
	my $COUNT_INTEGER = int($COUNT_THOUSAND) ; my $INTEGER_CHECK = $COUNT_THOUSAND -
$COUNT_INTEGER;
	if ($INTEGER_CHECK == 0) {print "Processing name $LINE_COUNT\n";}

	my $OTU_LINE_NUMBER = $. ; #print "Retrieving line $OTU_LINE_NUMBER\n" ;

	my $ACCNOS_LINE = $_; chomp ($ACCNOS_LINE);

	if ($ACCNOS_LINE !~ />/)      {
		$ACCNOS_LINE = ">$ACCNOS_LINE" ;
							}
	my $RETRIEVED_LINE = $HASH_COUNT{$ACCNOS_LINE};
	my @ARRAY_RETRIEVED_LINE = split('\t',$RETRIEVED_LINE) ;
	$LENGTH_TEST = scalar(@ARRAY_RETRIEVED_LINE) ;
#	my $LENGTH_TEST = length($RETRIEVED_LINE) ;
	if ($LENGTH_TEST > 1) {$OUTPUT = $OUTPUT.="$RETRIEVED_LINE\n" ;}

	if ($LENGTH_TEST < 2) {$NAMES_NOT_FOUND = $NAMES_NOT_FOUND.="$ACCNOS_LINE\n" ;}

					}
chop ($OUTPUT) ;
close (ACCNOS);

$NAMES_NOT_FOUND = $NAMES_NOT_FOUND.="\n\n" ;

print "Writing to output file\n" ;
open (OUTPUT, ">OUTPUT_retrieved_counts_from_$ACCNOS_FILE_NAME\.count") or die "Can't open $file
for reading" ;
#print OUTPUT "$NAMES_NOT_FOUND" ;
print OUTPUT "$OUTPUT" ;
close (OUTPUT);

print "Writing to output file\n" ;
open (OUTPUT_TEST, ">OUTPUT_names not found from_$ACCNOS_FILE_NAME\.count") or die "Can't open
$file for reading" ;
print OUTPUT_TEST "$NAMES_NOT_FOUND" ;
close (OUTPUT_TEST);
```

C.6 16S_OTU_read_count_tally_analysis_for_5P_to_C4_sorting_list_file_format2

```
$OTU_LIST_FILE_NAME = " Input_file_name.list" ;

$SAMPLE_NAMES_FILE = "RumiCell_Culture_Set1_sample_designators.txt" ;

@ARRAY_OTU_LIST_FILE_NAME = split('\.',$OTU_LIST_FILE_NAME) ;
$OTU_LIST_FILE_NAME_NO_EXTENSION = $ARRAY_OTU_LIST_FILE_NAME[0] ;
$OUTPUT = "Rep\t" ;
print "Loading sample names information into arrays\n" ;
$SAMPLE_NAMES_LIST = "" ; $DEFAULT_SAMPLE_VALUES = "" ;
open (SAMPLE_NAMES, "$SAMPLE_NAMES_FILE") or die "Can't open $file for reading" ;
while (<SAMPLE_NAMES>)        {
        my $SAMPLE_NAMES_LINE = $_ ; chomp ($SAMPLE_NAMES_LINE) ;
        $SAMPLE_NAMES_LIST = $SAMPLE_NAMES_LIST.="$SAMPLE_NAMES_LINE," ;
        $OUTPUT = $OUTPUT.="$SAMPLE_NAMES_LINE\t" ;
        $DEFAULT_SAMPLE_VALUES = $DEFAULT_SAMPLE_VALUES.="0\t" ;
                                                }
close (SAMPLE_NAMES) ;
chop($SAMPLE_NAMES_LIST) ; chop($OUTPUT) ; chop ($DEFAULT_SAMPLE_VALUES) ;
@ARRAY_SAMPLE_NAMES = split(',',$SAMPLE_NAMES_LIST) ;

@ARRAY_SAMPLE_VALUES = split('\t',$DEFAULT_SAMPLE_VALUES) ;
$SIZE_ARRAY_SAMPLE_NAMES = scalar(@ARRAY_SAMPLE_NAMES) ;

print "Starting read count analysis\n";
open (OTUS, "$OTU_LIST_FILE_NAME") or die "Can't open $file for reading" ;
$COUNTER_OTUS = 0 ;
while (<OTUS>)        {
#RESETTING VALUE FOR ARRAY SAMPLE VALUES
        my $COUNTER_ARRAY_RESET = 0 ;
        while ($COUNTER_ARRAY_RESET < $SIZE_ARRAY_SAMPLE_NAMES)      {
                $ARRAY_SAMPLE_VALUES[$COUNTER_ARRAY_RESET] = 0 ;
                $COUNTER_ARRAY_RESET = $COUNTER_ARRAY_RESET + 1 ;

                        }
        my $OTU_LINE = $_; chomp ($OTU_LINE);
        $COUNTER_OTUS = $COUNTER_OTUS + 1 ; my $COUNT_THOUSAND = $COUNTER_OTUS/10 ;
        my $COUNT_INTEGER = int($COUNT_THOUSAND) ; my $INTEGER_CHECK = $COUNT_THOUSAND -
$COUNT_INTEGER;
        if ($INTEGER_CHECK == 0) {print "Processing OTU $COUNTER_OTUS\n";   }

        my @ARRAY_OTU_LINE = split('\t',$OTU_LINE) ;
        my $FIRST_PHYLOTYPE = $ARRAY_OTU_LINE[0] ;

        my $OTU_LIST = $ARRAY_OTU_LINE[1] ;
        my @ARRAY_OTU_LIST = split(',', $OTU_LIST);
        my $NUMBER_OF_PHYLOTYPES = scalar(@ARRAY_OTU_LIST);
        $OUTPUT = $OUTPUT.="\n$FIRST_PHYLOTYPE" ;
        my $COUNTER_OTU_LIST = 0 ;
        while ($COUNTER_OTU_LIST < $NUMBER_OF_PHYLOTYPES)    {
                my $CURRENT_PHYLOTYPE = $ARRAY_OTU_LIST[$COUNTER_OTU_LIST];
                my $COUNTER_SAMPLE_VALUES = 0 ;
                while ($COUNTER_SAMPLE_VALUES < $SIZE_ARRAY_SAMPLE_NAMES)    {
                        my $DESIGNATOR_TO_TEST = $ARRAY_SAMPLE_NAMES[$COUNTER_SAMPLE_VALUES] ;
                        if ($CURRENT_PHYLOTYPE =~ /$DESIGNATOR_TO_TEST/)     {
                                my $PREVIOUS_COUNT = $ARRAY_SAMPLE_VALUES[$COUNTER_SAMPLE_VALUES] ;
                                my $UPDATED_COUNT = $PREVIOUS_COUNT + 1 ;
                                $ARRAY_SAMPLE_VALUES[$COUNTER_SAMPLE_VALUES] = $UPDATED_COUNT ;

                        }
                        $COUNTER_SAMPLE_VALUES = $COUNTER_SAMPLE_VALUES + 1 ;

                                }
                $COUNTER_OTU_LIST = $COUNTER_OTU_LIST + 1 ;

        }

        my $COUNTER_OUTPUT = 0 ;
        while ($COUNTER_OUTPUT < $SIZE_ARRAY_SAMPLE_NAMES)   {
                my $DATA_TO_TRANSFER = $ARRAY_SAMPLE_VALUES[$COUNTER_OUTPUT] ;
```

```
                $OUTPUT = $OUTPUT.="\t$DATA_TO_TRANSFER" ;
                $COUNTER_OUTPUT = $COUNTER_OUTPUT + 1 ;

        }
                                }
close (OTUS);

print "Writing to output file\n" ;
open (OUTPUT, ">OUTPUT_sample_itemized_read_counts_$OTU_LIST_FILE_NAME_NO_EXTENSION.count") or
die "Can't open $file for reading" ;
print OUTPUT "$OUTPUT" ;
close (OUTPUT);
```

C.7. 16S_OTU_Count_file_relative_abundance_from_itemized_count_output

```
$COUNT_FILE_NAME = " Input_file_name.count" ;

$OUTPUT = "" ;         $OUTPUT_TEST = "" ;
%HASH_COUNT ; %HASH_PERCENT ; @ARRAY_CUMULATIVE_SAMPLE ; $OTU_REP_LIST = "" ;

print "Loading count file information\n" ;
open (COUNT, "$COUNT_FILE_NAME") or die "Can't open $file for reading" ;
$LINE_COUNT = 0 ;
while (<COUNT>) {
        $LINE_COUNT = $LINE_COUNT + 1 ; my $COUNT_THOUSAND = $LINE_COUNT /1000 ;
        my $COUNT_INTEGER = int($COUNT_THOUSAND) ; my $INTEGER_CHECK = $COUNT_THOUSAND - 
$COUNT_INTEGER;
        if ($INTEGER_CHECK == 0) {print "Processing line $LINE_COUNT\n";}

        my $LINE = $_ ; chomp($LINE) ;
        my @ARRAY_COUNT_LINE = split('\t', $LINE); my $NUMBER_ARRAY_ELEMENTS = 
scalar(@ARRAY_COUNT_LINE) ;
        my $OTU_REP = $ARRAY_COUNT_LINE[0] ;
        $OTU_REP_LIST = $OTU_REP_LIST.="$OTU_REP," ;
        my $COUNT_ARRAY_CUMU_UPDATE = 1 ;
        while ($COUNT_ARRAY_CUMU_UPDATE < $NUMBER_ARRAY_ELEMENTS)   {
                my $CURRENT_COUNT_CELL = $ARRAY_COUNT_LINE[$COUNT_ARRAY_CUMU_UPDATE] ;
                my $UPDATE_CUMU_INFO = $ARRAY_CUMULATIVE_SAMPLE[$COUNT_ARRAY_CUMU_UPDATE] ;
                $UPDATE_CUMU_INFO = $UPDATE_CUMU_INFO + $CURRENT_COUNT_CELL ;
                $ARRAY_CUMULATIVE_SAMPLE[$COUNT_ARRAY_CUMU_UPDATE] = $UPDATE_CUMU_INFO ;

                $COUNT_ARRAY_CUMU_UPDATE = $COUNT_ARRAY_CUMU_UPDATE + 1 ;

                        }
        $HASH_COUNT{$OTU_REP} = $LINE ;
                                }
close (COUNT) ;
@ARRAY_OTU_REP_LIST = split(',',$OTU_REP_LIST) ;
$NUMBER_OTU_REPS = scalar(@ARRAY_OTU_REP_LIST) ;
$COUNT_OTU_REPS = 0 ;
print "Calculating relative abundance\n";
while ($COUNT_OTU_REPS < $NUMBER_OTU_REPS)   {
        my $COUNT_THOUSAND = $COUNT_OTU_REPS /1000 ;
        my $COUNT_INTEGER = int($COUNT_THOUSAND) ; my $INTEGER_CHECK = $COUNT_THOUSAND - 
$COUNT_INTEGER;
        if ($INTEGER_CHECK == 0) {print "Calculating for OTUrep $COUNT_OTU_REPS\n";}
#       print "Calculating for OTUrep $COUNT_OTU_REPS\n";
        my $CURRENT_OTU_REP = $ARRAY_OTU_REP_LIST[$COUNT_OTU_REPS] ;
        my $HASH_COUNT_LINE = $HASH_COUNT{$CURRENT_OTU_REP} ;
        $OUTPUT_TEST = $OUTPUT_TEST.="\n$CURRENT_OTU_REP\t$HASH_COUNT_LINE\n" ;
        if ($CURRENT_OTU_REP !~ />/)  {
                $HASH_PERCENT{$CURRENT_OTU_REP} = $HASH_COUNT_LINE ;
                                                                        }
        if ($CURRENT_OTU_REP =~ />/) {
#               $OUTPUT_TEST = $OUTPUT_TEST.="\n$CURRENT_OTU_REP\t" ;
                my @ARRAY_HASH_COUNT_LINE = split('\t',$HASH_COUNT_LINE) ;
                my $NUMBER_ELEMENTS_COUNT_LINE = scalar(@ARRAY_HASH_COUNT_LINE) ;
                my $PERCENT_COUNT_LINE = "$CURRENT_OTU_REP\t" ;
                my $COUNTS_COUNT_LINE = 1 ;
                while ($COUNTS_COUNT_LINE < $NUMBER_ELEMENTS_COUNT_LINE)    {
                        my $CURRENT_COUNT = $ARRAY_HASH_COUNT_LINE[$COUNTS_COUNT_LINE] ;
                        my $SAMPLE_CUMULATIVE_TALLY = $ARRAY_CUMULATIVE_SAMPLE[$COUNTS_COUNT_LINE]
;
#                       $OUTPUT_TEST = $OUTPUT_TEST.="$CURRENT_COUNT,$SAMPLE_CUMULATIVE_TALLY," ;
                        my $PERCENT_ABUNDANCE = $CURRENT_COUNT/$SAMPLE_CUMULATIVE_TALLY ;
                        $PERCENT_COUNT_LINE = $PERCENT_COUNT_LINE.="$PERCENT_ABUNDANCE\t" ;
#                       $OUTPUT_TEST = $OUTPUT_TEST.=",$PERCENT_ABUNDANCE\t" ;
                        $COUNTS_COUNT_LINE = $COUNTS_COUNT_LINE + 1 ;

                                }
                $HASH_PERCENT{$CURRENT_OTU_REP} = $PERCENT_COUNT_LINE ;
                                                                        }
        $COUNT_OTU_REPS = $COUNT_OTU_REPS + 1 ;
                                                                                }
```

```
print "Writing to output file\n" ;
$COUNT_OUTPUT = 0 ;
while ($COUNT_OUTPUT < $NUMBER_OTU_REPS)     {
       my $OTU_REP_OUTPUT = $ARRAY_OTU_REP_LIST[$COUNT_OUTPUT] ;
       my $PERCENT_TO_OUTPUT = $HASH_PERCENT{$OTU_REP_OUTPUT} ;
       chomp($PERCENT_TO_OUTPUT) ;
       $OUTPUT = $OUTPUT.="$PERCENT_TO_OUTPUT\n" ;
       $COUNT_OUTPUT = $COUNT_OUTPUT + 1 ;
                                                                                 }

open (OUTPUT, ">OUTPUT_counts_in_relative_abundance_$COUNT_FILE_NAME") or die "Can't open $file
for reading" ;
print OUTPUT "$OUTPUT" ;
close (OUTPUT);

open (OUTPUT_TEST, ">OUTPUT_TEST_relative_abundance_script.txt") or die "Can't open $file for
reading" ;
print OUTPUT_TEST "$OUTPUT_TEST" ;
close (OUTPUT_TEST);
```

D.1_Contig_sequential_building_from_kmer_input

```
$INPUT_FASTA_FILE = "Input_file_name.fasta" ;
$INPUT_FILE_KMER_SELECTION =
"testing_building_script_6c_starting_kmers_from_WP1_contigs_from_EF.kmer" ;

$CONTIG_CODE = "testing_building_script_version6c" ; $CONTIG_DESIGNATING_NUMBER = 0 ;
$LENGTH_SHORT_SEQ_TO_SCREEN = 30 ;    ####### UPDATE IF NEEDED
$BUILDING_CYCLE_UPDATE_INCREMENT = 500 ; ####### UPDATE IF NEEDED

$READS_USED_FOR_BUILDING = "" ; $READS_USED_FOR_BUILDING_ACCNOS = "" ;
$REDUNDANT_KMERS_FROM_INPUT_FILE = "" ;
############# DO NOT CHANGE   !!!!!!!!!!!!
$CONTIG_BUILDING_COMPLETED = 0 ; $OUTPUT_ASSEMBLIES_TOO_SHORT = "" ;
$LIST_DOWNSTREAM_BUILT_FILES = "" ; $LIST_UPSTREAM_BUILT_FILES = "" ;
$INFO_FASTA_FILE_KMER_SELECTION = "" ;
%HASH_READS_ALREADY_USED_FOR_KMERS ;
print "Loading sequences for kmers in array\n" ;
$COUNTER_FASTA_KMER_SELECTION = 0 ;
open (KMER_SELECTION, "$INPUT_FILE_KMER_SELECTION") or die "Can't open $file for reading" ;
while (<KMER_SELECTION>)      {
        $COUNTER_FASTA_KMER_SELECTION = $COUNTER_FASTA_KMER_SELECTION + 1 ; my
$KMER_COUNT_THOUSAND = $COUNTER_FASTA_KMER_SELECTION/100000 ;
        my $KMER_COUNT_INTEGER = int($KMER_COUNT_THOUSAND) ; my $KMER_INTEGER_CHECK =
$KMER_COUNT_THOUSAND - $KMER_COUNT_INTEGER;
        if ($KMER_INTEGER_CHECK == 0) {print "Loading sequence $COUNTER_FASTA_KMER_SELECTION\n";
        }

        my $FASTA_KMER_SELECTION_LINE = $_;   chomp ($FASTA_KMER_SELECTION_LINE);
        my $FASTA_KMER_SELECTION_SEQUENCE = $FASTA_KMER_SELECTION_LINE ;

        $INFO_FASTA_FILE_KMER_SELECTION =
$INFO_FASTA_FILE_KMER_SELECTION.="$FASTA_KMER_SELECTION_SEQUENCE\n" ;
                                                                }
close (KMER_SELECTION) ;

@ARRAY_INFO_FASTA_FILE_KMER_SELECTION = split('\n',$INFO_FASTA_FILE_KMER_SELECTION) ;
$NUMBER_KMERS_FOR_BUILDING = scalar(@ARRAY_INFO_FASTA_FILE_KMER_SELECTION) ;
$COUNTER_KMERS_FOR_BUILDING = 0 ;
while ($COUNTER_KMERS_FOR_BUILDING < $NUMBER_KMERS_FOR_BUILDING) {
        print "Building from kmer $COUNTER_KMERS_FOR_BUILDING of $NUMBER_KMERS_FOR_BUILDING\n" ;

        my $STARTING_SHORT_SEQ_FOR_SCREENING  =
$ARRAY_INFO_FASTA_FILE_KMER_SELECTION[$COUNTER_KMERS_FOR_BUILDING] ;

        my @ARRAY_READS_USED_FOR_BUILDING = split('\n',$READS_USED_FOR_BUILDING) ;
        my $NUMBER_READS_USED_FOR_BUILDING = scalar(@ARRAY_READS_USED_FOR_BUILDING) ;
        my $COUNTER_KMER_SCREENING = 0 ;
        my $KMER_FOUND_DURING_CHECK = 0 ; my $MATCHING_READ_FOUND = 0 ;
        print "Checking to see if kmer $STARTING_SHORT_SEQ_FOR_SCREENING was in reads used in
previous rounds\n" ;
        while ($COUNTER_KMER_SCREENING < $NUMBER_READS_USED_FOR_BUILDING and $MATCHING_READ_FOUND
< 1)    {
                my $CURRENT_READ_TO_CHECK =
$ARRAY_READS_USED_FOR_BUILDING[$COUNTER_KMER_SCREENING] ;
                if ($CURRENT_READ_TO_CHECK =~ /$STARTING_SHORT_SEQ_FOR_SCREENING/)  {
                        $KMER_FOUND_DURING_CHECK = 2 ;
                        $MATCHING_READ_FOUND = 2 ;
                        print "kmer $STARTING_SHORT_SEQ_FOR_SCREENING matched to reads previously
used\n" ;
                        $REDUNDANT_KMERS_FROM_INPUT_FILE =
$REDUNDANT_KMERS_FROM_INPUT_FILE.="$STARTING_SHORT_SEQ_FOR_SCREENING\n" ;

                                        }
                $COUNTER_KMER_SCREENING = $COUNTER_KMER_SCREENING + 1 ;

                                                                                        }

        if ($KMER_FOUND_DURING_CHECK < 1)     {
        print "kmer $STARTING_SHORT_SEQ_FOR_SCREENING not found in previously used reads\n" ;
```

```
        $CONTIG_DESIGNATING_NUMBER = $CONTIG_DESIGNATING_NUMBER + 1 ;
        my $CONTIG_NAME = "$CONTIG_CODE\_$CONTIG_DESIGNATING_NUMBER";
###### UPSTREAM BUILDING #######
        print "Starting kmer from first read:\t$STARTING_SHORT_SEQ_FOR_SCREENING\n" ;
        print "Upstream building in progress $CONTIG_NAME\n" ;
        my $UPSTREAM_NUMBER_BUILDING_CYCLES = 1 ;
        my $UPSTREAM_OUTPUT = "" ;
        my $UPSTREAM_OUTPUT_SUMMARY = "Building cycle\tScreening sequence\tNumber reads in
set\tStarting position\n" ;
        my $UPSTREAM_OUTPUT_SUMMARY_2 = "List of all candidate kmers for each round\n" ;
        my $UPSTREAM_SHORT_SEQ_FOR_SCREENING = $STARTING_SHORT_SEQ_FOR_SCREENING ;
        my $UPSTREAM_END_OF_CONTIG_REACHED = 0 ;
        while ($UPSTREAM_END_OF_CONTIG_REACHED  < 1 and $CONTIG_BUILDING_COMPLETED < 1) {
                print "Upstream screening for kmer $UPSTREAM_SHORT_SEQ_FOR_SCREENING, cycle
$UPSTREAM_NUMBER_BUILDING_CYCLES\n" ;
                my $UPSTREAM_COUNT = 0 ; my $UPSTREAM_CURRENT_READ_SET = "" ;
                open (UPSTREAM_FASTA, "$INPUT_FASTA_FILE") or die "Can't open $file for reading" ;
                while (<UPSTREAM_FASTA>)       {
                        my $UPSTREAM_LINE = $_;        chomp ($UPSTREAM_LINE);
                        if ($UPSTREAM_LINE =~ />/)     {
                                $UPSTREAM_COUNT = $UPSTREAM_COUNT + 1 ;       my
$UPSTREAM_COUNT_THOUSAND = $UPSTREAM_COUNT/20000 ;
                                my $UPSTREAM_COUNT_INTEGER = int($UPSTREAM_COUNT_THOUSAND) ;
                                my $UPSTREAM_INTEGER_CHECK = $UPSTREAM_COUNT_THOUSAND -
$UPSTREAM_COUNT_INTEGER;
                                if ($UPSTREAM_INTEGER_CHECK == 0)
                                {print "Upstream building $CONTIG_NAME round
$UPSTREAM_NUMBER_BUILDING_CYCLES\tScreening sequence $UPSTREAM_COUNT\n";   }
                                my $UPSTREAM_NAME = $UPSTREAM_LINE ;  my $UPSTREAM_SEQUENCE =
<UPSTREAM_FASTA> ; chomp ($UPSTREAM_SEQUENCE) ;
                                my $UPSTREAM_LENGTH_SEQ = length($UPSTREAM_SEQUENCE) ;
                                if ($UPSTREAM_SEQUENCE =~ /$UPSTREAM_SHORT_SEQ_FOR_SCREENING/)
        {
                                        if ($UPSTREAM_NUMBER_BUILDING_CYCLES < 11)           {
                                                $UPSTREAM_CURRENT_READ_SET =
$UPSTREAM_CURRENT_READ_SET.="$UPSTREAM_NAME\t$UPSTREAM_SEQUENCE\n" ;

                                }
                                        if ($UPSTREAM_NUMBER_BUILDING_CYCLES > 10)           {
                                                my @ARRAY_UPSTREAM_NAME =
split('\_rev',$UPSTREAM_NAME) ;
                                                my $UPSTREAM_NAME_FOR = $ARRAY_UPSTREAM_NAME[0] ;
                                                my $UPSTREAM_NAME_REV = "$UPSTREAM_NAME_FOR\_rev" ;

                                                my $CHECK_IF_READ_USED_ALREADY_FOR =
$HASH_READS_ALREADY_USED_FOR_KMERS{$UPSTREAM_NAME_FOR} ;
                                                my $CHECK_IF_READ_USED_ALREADY_REV =
$HASH_READS_ALREADY_USED_FOR_KMERS{$UPSTREAM_NAME_REV} ;

                                                if ($CHECK_IF_READ_USED_ALREADY_FOR !~ /Used/ and
$CHECK_IF_READ_USED_ALREADY_REV !~ /Used/)    {
                                                        $UPSTREAM_CURRENT_READ_SET =
$UPSTREAM_CURRENT_READ_SET.="$UPSTREAM_NAME\t$UPSTREAM_SEQUENCE\n" ;
                                                        $READS_USED_FOR_BUILDING =
$READS_USED_FOR_BUILDING.="$UPSTREAM_NAME\t$UPSTREAM_SEQUENCE\n" ;

        $HASH_READS_ALREADY_USED_FOR_KMERS{$UPSTREAM_NAME_FOR} = "Used" ;

        $HASH_READS_ALREADY_USED_FOR_KMERS{$UPSTREAM_NAME_REV} = "Used" ;
                                                        $READS_USED_FOR_BUILDING_ACCNOS =
$READS_USED_FOR_BUILDING_ACCNOS.="$UPSTREAM_NAME_FOR\n$UPSTREAM_NAME_REV\n" ;


                                }

                                }

                                                        }
                                                                                }
                                                                }
                close (UPSTREAM_FASTA) ;
```

```
                print "Upstream building: Determining position of reads\n"  ;
                my %UPSTREAM_HASH_POSITION_MAPPING ; my $UPSTREAM_HIGHEST_POSITION = 0 ;
                my @UPSTREAM_ARRAY_CURRENT_READ_SET = split('\n',$UPSTREAM_CURRENT_READ_SET) ;
                my $UPSTREAM_NUMBER_READS_IN_CURRENT_SET =
scalar(@UPSTREAM_ARRAY_CURRENT_READ_SET) ;
                my $UPSTREAM_COUNTER_ELEMENTS_CURRENT_READ_SET = 0 ;
                while ($UPSTREAM_COUNTER_ELEMENTS_CURRENT_READ_SET <
$UPSTREAM_NUMBER_READS_IN_CURRENT_SET) {
                        my $UPSTREAM_CURRENT_READ =
$UPSTREAM_ARRAY_CURRENT_READ_SET[$UPSTREAM_COUNTER_ELEMENTS_CURRENT_READ_SET] ;
                        my @UPSTREAM_ARRAY_CURRENT_READ = split('\t',$UPSTREAM_CURRENT_READ) ;
                        my $UPSTREAM_CURRENT_NAME = $UPSTREAM_ARRAY_CURRENT_READ[0] ;
                        my $UPSTREAM_CURRENT_SEQUENCE = $UPSTREAM_ARRAY_CURRENT_READ[1] ;
                        my $UPSTREAM_LENGTH_CURRRENT_SEQ = length($UPSTREAM_CURRENT_SEQUENCE) ;
                        my $UPSTREAM_LENGTH_SEQ_FOR_MAPPING =
length($UPSTREAM_SHORT_SEQ_FOR_SCREENING) ;
                        if ($UPSTREAM_CURRENT_SEQUENCE =~ /$UPSTREAM_SHORT_SEQ_FOR_SCREENING/)
        {
                                my $UPSTREAM_SEQ_POSITION = 0 ;
                                my $UPSTREAM_MAPPING_SEQ_FOUND = 0 ;
                                my $UPSTREAM_MAX_LENGTH_TO_SCREEN = $UPSTREAM_LENGTH_CURRRENT_SEQ -
$UPSTREAM_LENGTH_SEQ_FOR_MAPPING ;
                                while ($UPSTREAM_SEQ_POSITION < $UPSTREAM_MAX_LENGTH_TO_SCREEN and
$UPSTREAM_MAPPING_SEQ_FOUND < 1) {
                                        my $UPSTREAM_LENGTH_SEQ_TO_SCREEN = $UPSTREAM_SEQ_POSITION
+ $UPSTREAM_LENGTH_SEQ_FOR_MAPPING ;
                                        my $UPSTREAM_REGION_TO_SCREEN =
substr($UPSTREAM_CURRENT_SEQUENCE,0,$UPSTREAM_LENGTH_SEQ_TO_SCREEN) ;

                                        if ($UPSTREAM_REGION_TO_SCREEN =~
/$UPSTREAM_SHORT_SEQ_FOR_SCREENING/)  {
                                                $UPSTREAM_MAPPING_SEQ_FOUND = 2 ;
                                                my $UPSTREAM_HASH_INFO_TO_UPDATE =
$UPSTREAM_HASH_POSITION_MAPPING{$UPSTREAM_SEQ_POSITION} ;
                                                $UPSTREAM_HASH_INFO_TO_UPDATE =
$UPSTREAM_HASH_INFO_TO_UPDATE.="$UPSTREAM_CURRENT_READ\n" ;

        $UPSTREAM_HASH_POSITION_MAPPING{$UPSTREAM_SEQ_POSITION} = $UPSTREAM_HASH_INFO_TO_UPDATE ;

                                                if ($UPSTREAM_SEQ_POSITION >
$UPSTREAM_HIGHEST_POSITION)   {
                                                        $UPSTREAM_HIGHEST_POSITION =
$UPSTREAM_SEQ_POSITION;

                                                                }

                                                                                }
                                        $UPSTREAM_SEQ_POSITION = $UPSTREAM_SEQ_POSITION + 1 ;



                        }

                                                                }
                        $UPSTREAM_COUNTER_ELEMENTS_CURRENT_READ_SET =
$UPSTREAM_COUNTER_ELEMENTS_CURRENT_READ_SET + 1 ;

                                                                                                }


                print "Upstream building: Aligning and sorting reads\n"  ;
                my $UPSTREAM_COUNTER_SORTING = $UPSTREAM_HIGHEST_POSITION ;
                my $UPSTREAM_LOWEST_NUMBER_DASHES = 10000 ;
                my %UPSTREAM_HASH_LENGTH_SORTED ;
                my $UPSTREAM_DASHES_TO_ADD = 0 ;
                while ($UPSTREAM_COUNTER_SORTING >= 0) {
                        my $UPSTREAM_CONTENT_TO_SORT =
$UPSTREAM_HASH_POSITION_MAPPING{$UPSTREAM_COUNTER_SORTING} ;
                        my $UPSTREAM_SIZE_CONTENT_TO_SORT = length($UPSTREAM_CONTENT_TO_SORT) ;
                        if ($UPSTREAM_SIZE_CONTENT_TO_SORT > 1)              {
```

```
                              my @UPSTREAM_ARRAY_CONTENT_TO_SORT =
split('\n',$UPSTREAM_CONTENT_TO_SORT) ;
                              my $UPSTREAM_NUMBER_ARRAY_ELEMENTS =
scalar(@UPSTREAM_ARRAY_CONTENT_TO_SORT) ;
                              my $UPSTREAM_COUNTER_ARRAY_SORTING = 0 ;
                              while ($UPSTREAM_COUNTER_ARRAY_SORTING <
$UPSTREAM_NUMBER_ARRAY_ELEMENTS)      {
                                     my $UPSTREAM_CURRENT_ARRAY_ELEMENT_TO_SORT =
$UPSTREAM_ARRAY_CONTENT_TO_SORT[$UPSTREAM_COUNTER_ARRAY_SORTING] ;

                                     my @UPSTREAM_ARRAY_CURRENT_READ_2 =
split('\t',$UPSTREAM_CURRENT_ARRAY_ELEMENT_TO_SORT) ;
                                     my $UPSTREAM_CURRENT_READ_NAME_2 =
$UPSTREAM_ARRAY_CURRENT_READ_2[0] ;
                                     my $UPSTREAM_CURRENT_READ_SEQ_2 =
$UPSTREAM_ARRAY_CURRENT_READ_2[1] ;

                                     my $UPSTREAM_COUNTER_DASHES = 0 ; my $UPSTREAM_ALIGNED_SEQ
= "" ;
                                     while ($UPSTREAM_COUNTER_DASHES <= $UPSTREAM_DASHES_TO_ADD)
        {
                                            $UPSTREAM_ALIGNED_SEQ = $UPSTREAM_ALIGNED_SEQ.="\-"
;
                                            $UPSTREAM_COUNTER_DASHES = $UPSTREAM_COUNTER_DASHES
+ 1 ;

                                                           }
                                     if ($UPSTREAM_DASHES_TO_ADD <
$UPSTREAM_LOWEST_NUMBER_DASHES)       {
                                            $UPSTREAM_LOWEST_NUMBER_DASHES =
$UPSTREAM_DASHES_TO_ADD ;

                                                           }
                                     $UPSTREAM_ALIGNED_SEQ =
$UPSTREAM_ALIGNED_SEQ.="$UPSTREAM_CURRENT_READ_SEQ_2" ;

                                     my $UPSTREAM_LENGTH_ALIGNED_SEQ =
length($UPSTREAM_ALIGNED_SEQ) ;

                                     my $UPSTREAM_HASH_INFO_TO_UPDATE_2 =
$UPSTREAM_HASH_LENGTH_SORTED{$UPSTREAM_LENGTH_ALIGNED_SEQ} ;
                                     $UPSTREAM_HASH_INFO_TO_UPDATE_2 =
$UPSTREAM_HASH_INFO_TO_UPDATE_2.="$UPSTREAM_CURRENT_READ_NAME_2\t$UPSTREAM_ALIGNED_SEQ\n" ;
                                     $UPSTREAM_HASH_LENGTH_SORTED{$UPSTREAM_LENGTH_ALIGNED_SEQ}
= $UPSTREAM_HASH_INFO_TO_UPDATE_2 ;

                                     if ($UPSTREAM_LENGTH_ALIGNED_SEQ >
$UPSTREAM_LONGEST_ALIGNED)    {
                                            $UPSTREAM_LONGEST_ALIGNED =
$UPSTREAM_LENGTH_ALIGNED_SEQ ;

                                                           }
                                            $UPSTREAM_COUNTER_ARRAY_SORTING =
$UPSTREAM_COUNTER_ARRAY_SORTING + 1 ;

                                                                          }

               }
                      $UPSTREAM_COUNTER_SORTING =  $UPSTREAM_COUNTER_SORTING - 1 ;
                      $UPSTREAM_DASHES_TO_ADD = $UPSTREAM_DASHES_TO_ADD + 1 ;
                                                                                         }



               print "Upstream building Creating output and preparing for next round\n"  ;
               my $UPSTREAM_OUTPUT_INDIVIDUAL_ROUND = "" ;
               my $UPSTREAM_COUNTER_OUTPUT = 0 ; my $UPSTREAM_LOWEST_NUMBER_DASHES = 10000 ;
               $UPSTREAM_OUTPUT = $UPSTREAM_OUTPUT.="Round
$UPSTREAM_NUMBER_BUILDING_CYCLES\t$UPSTREAM_SHORT_SEQ_FOR_SCREENING\t$UPSTREAM_NUMBER_READS_IN_CU
RRENT_SET\n" ;
```

```
		while ($UPSTREAM_COUNTER_OUTPUT < $UPSTREAM_LONGEST_ALIGNED)	{
			my $UPSTREAM_CURRENT_SET_TO_OUTPUT =
$UPSTREAM_HASH_LENGTH_SORTED{$UPSTREAM_COUNTER_OUTPUT} ;
			chomp($UPSTREAM_CURRENT_SET_TO_OUTPUT) ;
			my $UPSTREAM_SIZE_TEST_FOR_OUTPUT =
length($UPSTREAM_CURRENT_SET_TO_OUTPUT) ;
			if ($UPSTREAM_SIZE_TEST_FOR_OUTPUT > 5)	{
				if ($UPSTREAM_COUNTER_OUTPUT < $UPSTREAM_LOWEST_NUMBER_DASHES)
	{
					$UPSTREAM_LOWEST_NUMBER_DASHES = $UPSTREAM_COUNTER_OUTPUT ;

								}
				$UPSTREAM_OUTPUT_INDIVIDUAL_ROUND =
$UPSTREAM_OUTPUT_INDIVIDUAL_ROUND.="$UPSTREAM_CURRENT_SET_TO_OUTPUT\n" ;
				$UPSTREAM_OUTPUT =
$UPSTREAM_OUTPUT.="$UPSTREAM_CURRENT_SET_TO_OUTPUT\n" ;

	}
			$UPSTREAM_COUNTER_OUTPUT = $UPSTREAM_COUNTER_OUTPUT + 1 ;

					}

		if ($UPSTREAM_OUTPUT_SUMMARY =~
/\t$UPSTREAM_SHORT_SEQ_FOR_SCREENING\t$UPSTREAM_NUMBER_READS_IN_CURRENT_SET/)	{
			$UPSTREAM_END_OF_CONTIG_REACHED = 2 ;
			print "Identical kmer selected for successive cycles - End of contig
upstream reached\n" ;


				}
		$UPSTREAM_OUTPUT_SUMMARY =
$UPSTREAM_OUTPUT_SUMMARY.="$UPSTREAM_NUMBER_BUILDING_CYCLES\t$UPSTREAM_SHORT_SEQ_FOR_SCREENING\t$
UPSTREAM_NUMBER_READS_IN_CURRENT_SET\n" ;

		$UPSTREAM_OUTPUT_SUMMARY_2 = $UPSTREAM_OUTPUT_SUMMARY_2.="Round
$UPSTREAM_NUMBER_BUILDING_CYCLES\n" ;

		my $UPSTREAM_FURTHEST_UPSTREAM_ALIGNED_SET =
$UPSTREAM_HASH_LENGTH_SORTED{$UPSTREAM_LOWEST_NUMBER_DASHES} ;
		my @UPSTREAM_ARRAY_FURTHEST_UPDTREAM_ALIGNED_SET =
split('\n',$UPSTREAM_FURTHEST_UPSTREAM_ALIGNED_SET) ;
		my $UPSTREAM_FURTHEST_UPSTREAM_ALIGNED_READ =
$UPSTREAM_ARRAY_FURTHEST_UPDTREAM_ALIGNED_SET[0] ;

		my @UPSTREAM_ARRAY_FURTHEST_UPSTREAM_ALIGNED_READ =
split('\t',$UPSTREAM_FURTHEST_UPSTREAM_ALIGNED_READ) ;
		my $UPSTREAM_FURTHEST_UPSTREAM_ALIGNED_SEQUENCE =
$UPSTREAM_ARRAY_FURTHEST_UPSTREAM_ALIGNED_READ[1] ;
		my $UPSTREAM_LENGTH_FURTHEST_UPSTREAM_ALIGNED_SEQUENCE =
length($UPSTREAM_FURTHEST_UPSTREAM_ALIGNED_SEQUENCE) ;
		my $UPSTREAM_STARTING_POSITION_NEXT_SHORT_SEQ_TO_SCREEN = 120 ;

		my $UPSTREAM_LIST_CANDIDATES_SHORT_SEQS = "" ; my
%UPSTREAM_HASH_CANDIDATES_SHORT_SEQS ;
		my $UPSTREAM_COUNTER_SHORT_SEQ_SCREEN = $UPSTREAM_LONGEST_ALIGNED ;
		while ($UPSTREAM_COUNTER_SHORT_SEQ_SCREEN > 0)	{
			my $UPSTREAM_CURRENT_SET_TO_TEST =
$UPSTREAM_HASH_LENGTH_SORTED{$UPSTREAM_COUNTER_SHORT_SEQ_SCREEN} ;
			chomp($UPSTREAM_CURRENT_SET_TO_TEST) ;
			my $UPSTREAM_SIZE_TEST_FOR_CURRENT_SET_TO_TEST =
length($UPSTREAM_CURRENT_SET_TO_TEST) ;
			if ($UPSTREAM_SIZE_TEST_FOR_CURRENT_SET_TO_TEST > 5)	{
				my @UPSTREAM_ARRAY_CURRENT_SET_TO_TEST =
split('\n',$UPSTREAM_CURRENT_SET_TO_TEST) ;
				my $UPSTREAM_NUMBER_SEQS_CURRENT_SET_TO_TEST =
scalar(@UPSTREAM_ARRAY_CURRENT_SET_TO_TEST) ;
				my $UPSTREAM_COUNTER_ARRAY_TO_TEST = 0 ;
				while ($UPSTREAM_COUNTER_ARRAY_TO_TEST <
$UPSTREAM_NUMBER_SEQS_CURRENT_SET_TO_TEST)	{
					my $UPSTREAM_CURRENT_SEQ_TO_TEST =
$UPSTREAM_ARRAY_CURRENT_SET_TO_TEST[$UPSTREAM_COUNTER_ARRAY_TO_TEST] ;
```

```
                                     my $UPSTREAM_CURRENT_CANDIDATE_SHORT_SEQ =
substr($UPSTREAM_CURRENT_SEQ_TO_TEST,$UPSTREAM_STARTING_POSITION_NEXT_SHORT_SEQ_TO_SCREEN,$LENGTH
_SHORT_SEQ_TO_SCREEN) ;
                                     $UPSTREAM_CURRENT_CANDIDATE_SHORT_SEQ =~ s/\-//g  ;
                                     my $UPSTREAM_LENGTH_TEST_CANDIDATE_SHORT_SEQ =
length($UPSTREAM_CURRENT_CANDIDATE_SHORT_SEQ) ;
                                     if ($UPSTREAM_LENGTH_TEST_CANDIDATE_SHORT_SEQ ==
$LENGTH_SHORT_SEQ_TO_SCREEN)  {
                                             my $UPSTREAM_UPDATE_INFO_HASH_CANDIDATES =
$UPSTREAM_HASH_CANDIDATES_SHORT_SEQS{$UPSTREAM_CURRENT_CANDIDATE_SHORT_SEQ} ;
                                             $UPSTREAM_UPDATE_INFO_HASH_CANDIDATES =
$UPSTREAM_UPDATE_INFO_HASH_CANDIDATES.="h," ;

        $UPSTREAM_HASH_CANDIDATES_SHORT_SEQS{$UPSTREAM_CURRENT_CANDIDATE_SHORT_SEQ} =
$UPSTREAM_UPDATE_INFO_HASH_CANDIDATES ;

                                             if ($UPSTREAM_LIST_CANDIDATES_SHORT_SEQS !~
/$UPSTREAM_CURRENT_CANDIDATE_SHORT_SEQ,/)     {
                                                     $UPSTREAM_LIST_CANDIDATES_SHORT_SEQS =
$UPSTREAM_LIST_CANDIDATES_SHORT_SEQS.="$UPSTREAM_CURRENT_CANDIDATE_SHORT_SEQ," ;


                }

                                                                     }
                                     $UPSTREAM_COUNTER_ARRAY_TO_TEST =
$UPSTREAM_COUNTER_ARRAY_TO_TEST + 1 ;


        }

                             }
                     $UPSTREAM_COUNTER_SHORT_SEQ_SCREEN = $UPSTREAM_COUNTER_SHORT_SEQ_SCREEN- 1
;

        }
             my @UPSTREAM_ARRAY_LIST_CANDIDATES_SHORT_SEQS =
split(',',$UPSTREAM_LIST_CANDIDATES_SHORT_SEQS) ;
             my $UPSTREAM_NUMBER_CANDIDATES_SHORT_SEQS =
scalar(@UPSTREAM_ARRAY_LIST_CANDIDATES_SHORT_SEQS) ;
             my $UPSTREAM_MOST_FREQUENT_SHORT_SEQ = "" ; my
$UPSTREAM_HIGHEST_FREQUENCY_SHORT_SEQ = 0 ;
             my $UPSTREAM_COUNTER_SCREEN_CANDIDATES_SHORT_SEQS = 0 ;
             while ($UPSTREAM_COUNTER_SCREEN_CANDIDATES_SHORT_SEQS <
$UPSTREAM_NUMBER_CANDIDATES_SHORT_SEQS)      {
                     my $UPSTREAM_CURRENT_CANDIDATE_SHORT_SEQ_TO_SCREEN =
$UPSTREAM_ARRAY_LIST_CANDIDATES_SHORT_SEQS[$UPSTREAM_COUNTER_SCREEN_CANDIDATES_SHORT_SEQS] ;
                     my $UPSTREAM_HASH_INFO_CANDIDATE_SHORT_SEQ_TO_SCREEN =
$UPSTREAM_HASH_CANDIDATES_SHORT_SEQS{$UPSTREAM_CURRENT_CANDIDATE_SHORT_SEQ_TO_SCREEN} ;
                     my @UPSTREAM_ARRAY_HASH_INFO_CANDIDATE_SHORT_SEQ_TO_SCREEN =
split(',',$UPSTREAM_HASH_INFO_CANDIDATE_SHORT_SEQ_TO_SCREEN) ;
                     my $UPSTREAM_FREQUENCY_CURRENT_CANDIDATE_SHORT_SEQ_TO_SCREEN =
scalar(@UPSTREAM_ARRAY_HASH_INFO_CANDIDATE_SHORT_SEQ_TO_SCREEN) ;
                     if ($UPSTREAM_FREQUENCY_CURRENT_CANDIDATE_SHORT_SEQ_TO_SCREEN >
$UPSTREAM_HIGHEST_FREQUENCY_SHORT_SEQ)       {
                             $UPSTREAM_HIGHEST_FREQUENCY_SHORT_SEQ =
$UPSTREAM_FREQUENCY_CURRENT_CANDIDATE_SHORT_SEQ_TO_SCREEN ;
                             $UPSTREAM_MOST_FREQUENT_SHORT_SEQ =
$UPSTREAM_CURRENT_CANDIDATE_SHORT_SEQ_TO_SCREEN ;


                     }
                     $UPSTREAM_OUTPUT_SUMMARY_2 =
$UPSTREAM_OUTPUT_SUMMARY_2.="\t$UPSTREAM_CURRENT_CANDIDATE_SHORT_SEQ_TO_SCREEN\t$UPSTREAM_FREQUEN
CY_CURRENT_CANDIDATE_SHORT_SEQ_TO_SCREEN\n" ;
                     $UPSTREAM_COUNTER_SCREEN_CANDIDATES_SHORT_SEQS =
$UPSTREAM_COUNTER_SCREEN_CANDIDATES_SHORT_SEQS + 1 ;


        }
             $UPSTREAM_SHORT_SEQ_FOR_SCREENING = $UPSTREAM_MOST_FREQUENT_SHORT_SEQ ;
```

```
                $UPSTREAM_OUTPUT = $UPSTREAM_OUTPUT.="End of round\n";

                #################### TEST FOR PROGRESS OUTPUT ####
                my $UPSTREAM_BUILDING_PROGRESS_TALLY =
$UPSTREAM_NUMBER_BUILDING_CYCLES/$BUILDING_CYCLE_UPDATE_INCREMENT ;
                my $UPSTREAM_BUILDING_PROGRESS_TALLY_INTEGER =
int($UPSTREAM_BUILDING_PROGRESS_TALLY) ;
                my $UPSTREAM_BUILDING_PROGRESS_INTEGER_CHECK = $UPSTREAM_BUILDING_PROGRESS_TALLY -
$UPSTREAM_BUILDING_PROGRESS_TALLY_INTEGER;

                if ($UPSTREAM_BUILDING_PROGRESS_INTEGER_CHECK == 0) {
                        open (OUTPUT_UPS_PROGRESS, ">OUTPUT $CONTIG_NAME
progress_upstream_building cycle $UPSTREAM_NUMBER_BUILDING_CYCLES.phylip") or die "Can't open
$file for reading" ;
                        print OUTPUT_UPS_PROGRESS "$UPSTREAM_OUTPUT" ;

                        close (OUTPUT_UPS_PROGRESS);

                }
                #################       END TEST FOR PROGRESS OUTPUT ####

                $UPSTREAM_NUMBER_BUILDING_CYCLES = $UPSTREAM_NUMBER_BUILDING_CYCLES + 1 ;


                                                                }
                print "Screen completed for upstream end\n" ;


                open (OUTPUT_UPS_END, ">OUTPUT $CONTIG_NAME end_upstream_building cycle
$UPSTREAM_NUMBER_BUILDING_CYCLES.phylip") or die "Can't open $file for reading" ;

                print OUTPUT_UPS_END "$UPSTREAM_OUTPUT" ;
                close (OUTPUT_UPS_END);

###### UPSTREAM BUILDING END#######

###### DOWNSTREAM BUILDING #######
        print "Starting kmer from first read:\t$STARTING_SHORT_SEQ_FOR_SCREENING\n" ;
        print "Downstream building in progress $CONTIG_NAME\n" ;
        my $NUMBER_BUILDING_CYCLES = 1 ;
        my $OUTPUT = "" ;
        my $OUTPUT_SUMMARY = "Building cycle\tScreening sequence\tNumber reads in set\tStarting
position\n" ;
        my $OUTPUT_SUMMARY_2 = "List of all candidate kmers for each round\n" ;
        my $SHORT_SEQ_FOR_SCREENING = $STARTING_SHORT_SEQ_FOR_SCREENING ;
        my $END_OF_CONTIG_REACHED = 0 ;
        while ($END_OF_CONTIG_REACHED  < 1 and $CONTIG_BUILDING_COMPLETED < 1) {
                print "Screening for kmer $SHORT_SEQ_FOR_SCREENING, cycle
$NUMBER_BUILDING_CYCLES\n" ;
                my $COUNT = 0 ; my $CURRENT_READ_SET = "" ;
                open (FASTA, "$INPUT_FASTA_FILE") or die "Can't open $file for reading" ;
                while (<FASTA>)        {
                        my $LINE = $_; chomp ($LINE);
                        if ($LINE =~ />/)      {
                                $COUNT = $COUNT + 1 ;  my $COUNT_THOUSAND = $COUNT/20000 ;
                                my $COUNT_INTEGER = int($COUNT_THOUSAND) ; my $INTEGER_CHECK =
$COUNT_THOUSAND - $COUNT_INTEGER;
                                if ($INTEGER_CHECK == 0) {print "Downstream building $CONTIG_NAME
round $NUMBER_BUILDING_CYCLES\tScreening sequence $COUNT\n";        }
                                my $NAME = $LINE ; my $SEQUENCE = <FASTA> ; chomp ($SEQUENCE) ; my
$LENGTH_SEQ = length($SEQUENCE) ;
                                if ($SEQUENCE =~ /$SHORT_SEQ_FOR_SCREENING/)         {
                                        if ($NUMBER_BUILDING_CYCLES < 11)            {
                                                $CURRENT_READ_SET =
$CURRENT_READ_SET.="$NAME\t$SEQUENCE\n" ;

                }

                                        if ($NUMBER_BUILDING_CYCLES > 10)            {
                                                my @ARRAY_DOWNSTREAM_NAME = split('\_rev',$NAME) ;
```

```
                                        my $DOWNSTREAM_NAME_FOR = $ARRAY_DOWNSTREAM_NAME[0]
;
                                        my $DOWNSTREAM_NAME_REV =
"$DOWNSTREAM_NAME_FOR\_rev" ;

                                        my $CHECK_IF_READ_USED_ALREADY_FOR =
$HASH_READS_ALREADY_USED_FOR_KMERS{$DOWNSTREAM_NAME_FOR} ;
                                        my $CHECK_IF_READ_USED_ALREADY_REV =
$HASH_READS_ALREADY_USED_FOR_KMERS{$DOWNSTREAM_NAME_REV} ;

                                        if ($CHECK_IF_READ_USED_ALREADY_FOR !~ /Used/ and
$CHECK_IF_READ_USED_ALREADY_REV !~ /Used/)   {
                                                $CURRENT_READ_SET =
$CURRENT_READ_SET.="$NAME\t$SEQUENCE\n" ;
                                                $READS_USED_FOR_BUILDING =
$READS_USED_FOR_BUILDING.="$NAME\t$SEQUENCE\n" ;

        $HASH_READS_ALREADY_USED_FOR_KMERS{$DOWNSTREAM_NAME_FOR} = "Used" ;

        $HASH_READS_ALREADY_USED_FOR_KMERS{$DOWNSTREAM_NAME_REV} = "Used" ;
                                                $READS_USED_FOR_BUILDING_ACCNOS =
$READS_USED_FOR_BUILDING_ACCNOS.="$DOWNSTREAM_NAME_FOR\n$DOWNSTREAM_NAME_REV\n" ;


                                }

                }


                        }
                                                        }
                                                }
                close (FASTA) ;

                print "Downstream building: Determining position of reads\n"  ;
                my %HASH_POSITION_MAPPING ; my $HIGHEST_POSITION = 0 ;
                my @ARRAY_CURRENT_READ_SET = split('\n',$CURRENT_READ_SET) ;
                my $NUMBER_READS_IN_CURRENT_SET = scalar(@ARRAY_CURRENT_READ_SET) ;
                my $COUNTER_ELEMENTS_CURRENT_READ_SET = 0 ;
                while ($COUNTER_ELEMENTS_CURRENT_READ_SET < $NUMBER_READS_IN_CURRENT_SET) {
                        my $CURRENT_READ =
$ARRAY_CURRENT_READ_SET[$COUNTER_ELEMENTS_CURRENT_READ_SET] ;
                        my @ARRAY_CURRENT_READ = split('\t',$CURRENT_READ) ;
                        my $CURRENT_NAME = $ARRAY_CURRENT_READ[0] ;
                        my $CURRENT_SEQUENCE = $ARRAY_CURRENT_READ[1] ;
                        my $LENGTH_CURRRENT_SEQ = length($CURRENT_SEQUENCE) ;
                        my $LENGTH_SEQ_FOR_MAPPING = length($SHORT_SEQ_FOR_SCREENING) ;

                        if ($CURRENT_SEQUENCE =~ /$SHORT_SEQ_FOR_SCREENING/)        {
                                my $SEQ_POSITION = 0 ;
                                my $MAPPING_SEQ_FOUND = 0 ;
                                my $MAX_LENGTH_TO_SCREEN = $LENGTH_CURRRENT_SEQ -
$LENGTH_SEQ_FOR_MAPPING ;
                                while ($SEQ_POSITION < $MAX_LENGTH_TO_SCREEN and $MAPPING_SEQ_FOUND
< 1) {
                                        my $LENGTH_SEQ_TO_SCREEN = $SEQ_POSITION +
$LENGTH_SEQ_FOR_MAPPING ;
                                        my $REGION_TO_SCREEN =
substr($CURRENT_SEQUENCE,0,$LENGTH_SEQ_TO_SCREEN) ;

                                        if ($REGION_TO_SCREEN =~ /$SHORT_SEQ_FOR_SCREENING/)
        {
                                                $MAPPING_SEQ_FOUND = 2 ;
                                                my $HASH_INFO_TO_UPDATE =
$HASH_POSITION_MAPPING{$SEQ_POSITION} ;
                                                $HASH_INFO_TO_UPDATE =
$HASH_INFO_TO_UPDATE.="$CURRENT_READ\n" ;
                                                $HASH_POSITION_MAPPING{$SEQ_POSITION} =
$HASH_INFO_TO_UPDATE ;

                                                if ($SEQ_POSITION > $HIGHEST_POSITION)       {
```

```
                                                    $HIGHEST_POSITION = $SEQ_POSITION;

                        }

                                        }
                                $SEQ_POSITION = $SEQ_POSITION + 1 ;


                                                        }

                                }
                $COUNTER_ELEMENTS_CURRENT_READ_SET = $COUNTER_ELEMENTS_CURRENT_READ_SET + 1 ;

                                                }
                print "Downstream building: Aligning and sorting reads\n"  ;
                my $COUNTER_SORTING = $HIGHEST_POSITION ;
                my $LONGEST_ALIGNED = 0 ;
                my %HASH_LENGTH_SORTED ;
                my $DASHES_TO_ADD = 0 ;
                while ($COUNTER_SORTING >= 0) {
                        my $CONTENT_TO_SORT = $HASH_POSITION_MAPPING{$COUNTER_SORTING} ;
                        my $SIZE_CONTENT_TO_SORT = length($CONTENT_TO_SORT) ;
                        if ($SIZE_CONTENT_TO_SORT > 1)              {
                                my @ARRAY_CONTENT_TO_SORT = split('\n',$CONTENT_TO_SORT) ;
                                my $NUMBER_ARRAY_ELEMENTS = scalar(@ARRAY_CONTENT_TO_SORT) ;
                                my $COUNTER_ARRAY_SORTING = 0 ;
                                while ($COUNTER_ARRAY_SORTING < $NUMBER_ARRAY_ELEMENTS)     {
                                        my $CURRENT_ARRAY_ELEMENT_TO_SORT =
$ARRAY_CONTENT_TO_SORT[$COUNTER_ARRAY_SORTING] ;

                                        my @ARRAY_CURRENT_READ_2 =
split('\t',$CURRENT_ARRAY_ELEMENT_TO_SORT) ;
                                        my $CURRENT_READ_NAME_2 = $ARRAY_CURRENT_READ_2[0] ;
                                        my $CURRENT_READ_SEQ_2 = $ARRAY_CURRENT_READ_2[1] ;

                                        my $COUNTER_DASHES = 0 ; my $ALIGNED_SEQ = "" ;
                                        while ($COUNTER_DASHES <= $DASHES_TO_ADD)    {
                                                $ALIGNED_SEQ = $ALIGNED_SEQ.="\-" ;
                                                $COUNTER_DASHES = $COUNTER_DASHES + 1 ;


                        }
                                        $ALIGNED_SEQ = $ALIGNED_SEQ.="$CURRENT_READ_SEQ_2" ;
                                        my $LENGTH_ALIGNED_SEQ = length($ALIGNED_SEQ) ;
                                        my $HASH_INFO_TO_UPDATE_2 =
$HASH_LENGTH_SORTED{$LENGTH_ALIGNED_SEQ} ;
                                        $HASH_INFO_TO_UPDATE_2 =
$HASH_INFO_TO_UPDATE_2.="$CURRENT_READ_NAME_2\t$ALIGNED_SEQ\n" ;
                                        $HASH_LENGTH_SORTED{$LENGTH_ALIGNED_SEQ} =
$HASH_INFO_TO_UPDATE_2 ;

                                        if ($LENGTH_ALIGNED_SEQ > $LONGEST_ALIGNED) {
                                                $LONGEST_ALIGNED = $LENGTH_ALIGNED_SEQ ;

                        }
                                        $COUNTER_ARRAY_SORTING = $COUNTER_ARRAY_SORTING + 1 ;

                                                }
                                                                                        }
                        $COUNTER_SORTING =  $COUNTER_SORTING - 1 ;
                        $DASHES_TO_ADD = $DASHES_TO_ADD + 1 ;
                                                                        }




                print "Downstream building: Creating output and preparing for next round\n"  ;
                my $OUTPUT_INDIVIDUAL_ROUND = "" ;
                my $COUNTER_OUTPUT = $LONGEST_ALIGNED ;
                $OUTPUT = $OUTPUT.="Round
$NUMBER_BUILDING_CYCLES\t$SHORT_SEQ_FOR_SCREENING\t$NUMBER_READS_IN_CURRENT_SET\n" ;
```

```
		while ($COUNTER_OUTPUT > 0)	{
			my $CURRENT_SET_TO_OUTPUT = $HASH_LENGTH_SORTED{$COUNTER_OUTPUT} ;
			chomp($CURRENT_SET_TO_OUTPUT) ;
			my $SIZE_TEST_FOR_OUTPUT = length($CURRENT_SET_TO_OUTPUT) ;
			if ($SIZE_TEST_FOR_OUTPUT > 5)		{
				$OUTPUT_INDIVIDUAL_ROUND =
$OUTPUT_INDIVIDUAL_ROUND.="$CURRENT_SET_TO_OUTPUT\n" ;
				$OUTPUT = $OUTPUT.="$CURRENT_SET_TO_OUTPUT\n" ;
										}
			$COUNTER_OUTPUT = $COUNTER_OUTPUT - 1 ;
								}
		if ($OUTPUT_SUMMARY =~ /\t$SHORT_SEQ_FOR_SCREENING\t$NUMBER_READS_IN_CURRENT_SET/)
	{
			$END_OF_CONTIG_REACHED = 2 ;
			print "Identical kmer selected for successive cycles - End of $CONTIG_NAME
downstream reached\n" ;

								}
		$OUTPUT_SUMMARY =
$OUTPUT_SUMMARY.="$NUMBER_BUILDING_CYCLES\t$SHORT_SEQ_FOR_SCREENING\t$NUMBER_READS_IN_CURRENT_SET
\n" ;
		$OUTPUT_SUMMARY_2 = $OUTPUT_SUMMARY_2.="Round $NUMBER_BUILDING_CYCLES\n" ;
		my $FURTHEST_DOWNSTREAM_ALIGNED_SET = $HASH_LENGTH_SORTED{$LONGEST_ALIGNED} ;
		my @ARRAY_FURTHEST_DOWNSTREAM_ALIGNED_SET =
split('\n',$FURTHEST_DOWNSTREAM_ALIGNED_SET) ;
		my $FURTHEST_DOWNSTREAM_ALIGNED_READ = $ARRAY_FURTHEST_DOWNSTREAM_ALIGNED_SET[0] ;
		my @ARRAY_FURTHEST_DOWNSTREAM_ALIGNED_READ =
split('\t',$FURTHEST_DOWNSTREAM_ALIGNED_READ) ;
		my $FURTHEST_DOWNSTREAM_ALIGNED_SEQUENCE =
$ARRAY_FURTHEST_DOWNSTREAM_ALIGNED_READ[1] ;
		my $LENGTH_FURTHEST_DOWNSTREAM_ALIGNED_SEQUENCE =
length($FURTHEST_DOWNSTREAM_ALIGNED_SEQUENCE) ;
		my $STARTING_POSITION_NEXT_SHORT_SEQ_TO_SCREEN =
$LENGTH_FURTHEST_DOWNSTREAM_ALIGNED_SEQUENCE - $LENGTH_SHORT_SEQ_TO_SCREEN - 100 ;

		my $LIST_CANDIDATES_SHORT_SEQS = "" ; my %HASH_CANDIDATES_SHORT_SEQS ;
		my $COUNTER_SHORT_SEQ_SCREEN = $LONGEST_ALIGNED ;
		while ($COUNTER_SHORT_SEQ_SCREEN > 0)		{
			my $CURRENT_SET_TO_TEST = $HASH_LENGTH_SORTED{$COUNTER_SHORT_SEQ_SCREEN} ;
			chomp($CURRENT_SET_TO_TEST) ;
			my $SIZE_TEST_FOR_CURRENT_SET_TO_TEST = length($CURRENT_SET_TO_TEST) ;
			if ($SIZE_TEST_FOR_CURRENT_SET_TO_TEST > 5)	{
				my @ARRAY_CURRENT_SET_TO_TEST = split('\n',$CURRENT_SET_TO_TEST) ;
				my $NUMBER_SEQS_CURRENT_SET_TO_TEST =
scalar(@ARRAY_CURRENT_SET_TO_TEST) ;
				my $COUNTER_ARRAY_TO_TEST = 0 ;
				while ($COUNTER_ARRAY_TO_TEST < $NUMBER_SEQS_CURRENT_SET_TO_TEST)
	{
					my $CURRENT_SEQ_TO_TEST =
$ARRAY_CURRENT_SET_TO_TEST[$COUNTER_ARRAY_TO_TEST] ;
					my $CURRENT_CANDIDATE_SHORT_SEQ =
substr($CURRENT_SEQ_TO_TEST,$STARTING_POSITION_NEXT_SHORT_SEQ_TO_SCREEN,$LENGTH_SHORT_SEQ_TO_SCRE
EN) ;
					my $LENGTH_TEST_CANDIDATE_SHORT_SEQ =
length($CURRENT_CANDIDATE_SHORT_SEQ) ;
					if ($LENGTH_TEST_CANDIDATE_SHORT_SEQ ==
$LENGTH_SHORT_SEQ_TO_SCREEN)	{
						my $UPDATE_INFO_HASH_CANDIDATES =
$HASH_CANDIDATES_SHORT_SEQS{$CURRENT_CANDIDATE_SHORT_SEQ} ;
						$UPDATE_INFO_HASH_CANDIDATES =
$UPDATE_INFO_HASH_CANDIDATES.="h," ;

	$HASH_CANDIDATES_SHORT_SEQS{$CURRENT_CANDIDATE_SHORT_SEQ} = $UPDATE_INFO_HASH_CANDIDATES
;

						if ($LIST_CANDIDATES_SHORT_SEQS !~
/$CURRENT_CANDIDATE_SHORT_SEQ,/)	{
							$LIST_CANDIDATES_SHORT_SEQS =
$LIST_CANDIDATES_SHORT_SEQS.="$CURRENT_CANDIDATE_SHORT_SEQ," ;

										}
```

```
                                                                  }
                                    $COUNTER_ARRAY_TO_TEST = $COUNTER_ARRAY_TO_TEST + 1 ;

                                                        }

               }
                       $COUNTER_SHORT_SEQ_SCREEN = $COUNTER_SHORT_SEQ_SCREEN- 1 ;
                                                                                      }
               my @ARRAY_LIST_CANDIDATES_SHORT_SEQS = split(',',$LIST_CANDIDATES_SHORT_SEQS) ;
               my $NUMBER_CANDIDATES_SHORT_SEQS = scalar(@ARRAY_LIST_CANDIDATES_SHORT_SEQS) ;
               my $MOST_FREQUENT_SHORT_SEQ = "" ; my $HIGHEST_FREQUENCY_SHORT_SEQ = 0 ;
               my $COUNTER_SCREEN_CANDIDATES_SHORT_SEQS = 0 ;
               while ($COUNTER_SCREEN_CANDIDATES_SHORT_SEQS < $NUMBER_CANDIDATES_SHORT_SEQS)
       {
                       my $CURRENT_CANDIDATE_SHORT_SEQ_TO_SCREEN =
$ARRAY_LIST_CANDIDATES_SHORT_SEQS[$COUNTER_SCREEN_CANDIDATES_SHORT_SEQS] ;
                       my $HASH_INFO_CANDIDATE_SHORT_SEQ_TO_SCREEN =
$HASH_CANDIDATES_SHORT_SEQS{$CURRENT_CANDIDATE_SHORT_SEQ_TO_SCREEN} ;
                       my @ARRAY_HASH_INFO_CANDIDATE_SHORT_SEQ_TO_SCREEN =
split(',',$HASH_INFO_CANDIDATE_SHORT_SEQ_TO_SCREEN) ;
                       my $FREQUENCY_CURRENT_CANDIDATE_SHORT_SEQ_TO_SCREEN =
scalar(@ARRAY_HASH_INFO_CANDIDATE_SHORT_SEQ_TO_SCREEN) ;
                       if ($FREQUENCY_CURRENT_CANDIDATE_SHORT_SEQ_TO_SCREEN >
$HIGHEST_FREQUENCY_SHORT_SEQ)        {
                               $HIGHEST_FREQUENCY_SHORT_SEQ =
$FREQUENCY_CURRENT_CANDIDATE_SHORT_SEQ_TO_SCREEN ;
                               $MOST_FREQUENT_SHORT_SEQ = $CURRENT_CANDIDATE_SHORT_SEQ_TO_SCREEN ;

                                                                                      }
                       $OUTPUT_SUMMARY_2 =
$OUTPUT_SUMMARY_2.="\t$CURRENT_CANDIDATE_SHORT_SEQ_TO_SCREEN\t$FREQUENCY_CURRENT_CANDIDATE_SHORT_
SEQ_TO_SCREEN\n" ;
                       $COUNTER_SCREEN_CANDIDATES_SHORT_SEQS =
$COUNTER_SCREEN_CANDIDATES_SHORT_SEQS + 1 ;

                                                                     }
               $SHORT_SEQ_FOR_SCREENING = $MOST_FREQUENT_SHORT_SEQ ;
               $OUTPUT = $OUTPUT.="End of round\n";

                               ##################### TEST FOR PROGRESS OUTPUT ####
               my $DOWNSTREAM_BUILDING_PROGRESS_TALLY =
$NUMBER_BUILDING_CYCLES/$BUILDING_CYCLE_UPDATE_INCREMENT ;
               my $DOWNSTREAM_BUILDING_PROGRESS_TALLY_INTEGER =
int($DOWNSTREAM_BUILDING_PROGRESS_TALLY) ;
               my $DOWNSTREAM_BUILDING_PROGRESS_INTEGER_CHECK =
$DOWNSTREAM_BUILDING_PROGRESS_TALLY - $DOWNSTREAM_BUILDING_PROGRESS_TALLY_INTEGER;

               if ($DOWNSTREAM_BUILDING_PROGRESS_INTEGER_CHECK == 0) {
                       open (OUTPUT_DOWN_PROGRESS, ">OUTPUT $CONTIG_NAME
progress_downstream_building cycle $NUMBER_BUILDING_CYCLES.phylip") or die "Can't open $file for
reading" ;
                       print OUTPUT_DOWN_PROGRESS "$OUTPUT" ;

               close (OUTPUT_DOWN_PROGRESS);                                         }
               #################      END TEST FOR PROGRESS OUTPUT ####

               $NUMBER_BUILDING_CYCLES = $NUMBER_BUILDING_CYCLES + 1 ;

                                             }
               print "Screen completed for $CONTIG_NAME downstream end\n" ;
               ###### DOWNSTREAM BUILDING END #######

               #### PREPARING OUTPUT FILES #####
               if ($UPSTREAM_NUMBER_BUILDING_CYCLES >= 15 or $NUMBER_BUILDING_CYCLES >= 15)
       {
                       open (OUTPUT_SUMMARY, ">OUTPUT_kmers_used $CONTIG_NAME
downstream_building.txt") or die "Can't open $file for reading" ;
                       print OUTPUT_SUMMARY "$OUTPUT_SUMMARY" ;
```

```
                        close (OUTPUT_SUMMARY);

                        open (OUTPUT_SUMMARY_2, ">OUTPUT_kmers_list $CONTIG_NAME
downstream_building.txt") or die "Can't open $file for reading" ;
                        print OUTPUT_SUMMARY_2 "$OUTPUT_SUMMARY_2" ;

                        close (OUTPUT_SUMMARY_2);
                        open (OUTPUT, ">OUTPUT $CONTIG_NAME downstream_building.phylip") or die
"Can't open $file for reading" ;
                        print OUTPUT "$OUTPUT" ;
                        close (OUTPUT);

                        $LIST_DOWNSTREAM_BUILT_FILES = $LIST_DOWNSTREAM_BUILT_FILES.="OUTPUT
$CONTIG_NAME downstream_building.phylip\n" ;
                        open (OUTPUT_DOWNSTREAM_LIST, ">OUTPUT Master downstream_contigs files
$CONTIG_CODE.txt") or die "Can't open $file for reading" ;
                        print OUTPUT_DOWNSTREAM_LIST "$LIST_DOWNSTREAM_BUILT_FILES" ;

                        close (OUTPUT_DOWNSTREAM_LIST);

                        open (UPSTREAM_OUTPUT, ">OUTPUT $CONTIG_NAME upstream_building.phylip") or
die "Can't open $file for reading" ;
                        print UPSTREAM_OUTPUT "$UPSTREAM_OUTPUT" ;

                        close (UPSTREAM_OUTPUT);
                        open (UPSTREAM_OUTPUT_SUMMARY, ">OUTPUT_kmers_used $CONTIG_NAME
upstream_building.txt") or die "Can't open $file for reading" ;
                        print UPSTREAM_OUTPUT_SUMMARY "$UPSTREAM_OUTPUT_SUMMARY" ;

                        close (UPSTREAM_OUTPUT_SUMMARY);

                        open (UPSTREAM_OUTPUT_SUMMARY_2, ">OUTPUT_kmers_list $CONTIG_NAME
upstream_building.txt") or die "Can't open $file for reading" ;
                        print UPSTREAM_OUTPUT_SUMMARY_2 "$UPSTREAM_OUTPUT_SUMMARY_2" ;

                        close (UPSTREAM_OUTPUT_SUMMARY_2);

                        $LIST_UPSTREAM_BUILT_FILES = $LIST_UPSTREAM_BUILT_FILES.="OUTPUT
$CONTIG_NAME upstream_building.phylip\n" ;
                        open (OUTPUT_UPSTREAM_LIST, ">OUTPUT Master upstream_contigs files
$CONTIG_CODE.txt") or die "Can't open $file for reading" ;
                        print OUTPUT_UPSTREAM_LIST "$LIST_UPSTREAM_BUILT_FILES" ;

                        close (OUTPUT_UPSTREAM_LIST);


                                                                                            }
                if ($UPSTREAM_NUMBER_BUILDING_CYCLES < 15 and $NUMBER_BUILDING_CYCLES < 15)
        {
                        $OUTPUT_ASSEMBLIES_TOO_SHORT =
$OUTPUT_ASSEMBLIES_TOO_SHORT.="$CONTIG_NAME\n" ;
                        $OUTPUT_ASSEMBLIES_TOO_SHORT =
$OUTPUT_ASSEMBLIES_TOO_SHORT.="$UPSTREAM_OUTPUT_SUMMARY\n$UPSTREAM_OUTPUT\n" ;
                        $OUTPUT_ASSEMBLIES_TOO_SHORT =
$OUTPUT_ASSEMBLIES_TOO_SHORT.="$OUTPUT_SUMMARY\n$OUTPUT\n\n" ;

                        open (TOO_SHORT_OUTPUT, ">OUTPUT $CONTIG_CODE assemblies too short.txt")
or die "Can't open $file for reading" ;
                        print TOO_SHORT_OUTPUT "$OUTPUT_ASSEMBLIES_TOO_SHORT" ;

                        close (TOO_SHORT_OUTPUT);

                                                                            }


        open (READS_USED_OUTPUT, ">OUTPUT $CONTIG_CODE reads already used.phylip") or die "Can't
open $file for reading" ;
        print READS_USED_OUTPUT "$READS_USED_FOR_BUILDING" ;
        close (READS_USED_OUTPUT) ;
```

```
        open (SKIPPED_KMERS_OUTPUT, ">OUTPUT $CONTIG_CODE redundant kmers from seed file.kmer")
or die "Can't open $file for reading" ;
        print SKIPPED_KMERS_OUTPUT "$REDUNDANT_KMERS_FROM_INPUT_FILE" ;
        close (SKIPPED_KMERS_OUTPUT) ;

        open (READS_USED_ACCNOS_OUTPUT, ">OUTPUT $CONTIG_CODE reads already used.accnos") or die
"Can't open $file for reading" ;
        print READS_USED_ACCNOS_OUTPUT "$READS_USED_FOR_BUILDING_ACCNOS" ;
        close (READS_USED_ACCNOS_OUTPUT) ;

                                                                   }
        $COUNTER_KMERS_FOR_BUILDING = $COUNTER_KMERS_FOR_BUILDING + 1 ;

                                    }
```

D.2_Contig_consensus_from_Upstream_building_outputs

```
$NAME_OF_MASTER_FILE = "Master_file_input_name.txt" ;

open (MASTER, "$NAME_OF_MASTER_FILE") or die "Can't open $file for reading" ;
while (<MASTER>) {
	my $LINE_MASTER_FILE = $_ ; chomp($LINE_MASTER_FILE) ;
	my $INPUT_FILE = $LINE_MASTER_FILE ;
	my @ARRAY_INPUT_FILE = split('\.',$INPUT_FILE) ;
	my	$NAME_OF_FILE = $ARRAY_INPUT_FILE[0] ;

	my @ARRAY_NAME_OF_FILE = split(' ',$NAME_OF_FILE) ;
	my $CONTIG_NAME = $ARRAY_NAME_OF_FILE[1] ;

	my $PRE_OUTPUT = "" ; my $OUTPUT = "" ; my $OUTPUT_TEST = "" ;
	my $CONTIG_CONSENSUS = "" ;   my $OUTPUT_SUMMARY = "" ;

	print "Processing file $NAME_OF_FILE, assessing nucleotide consensus by position\n" ;
	my $COUNT = 0 ;
	open (FILE, "$INPUT_FILE") or print "Can't open $file for reading\n" ;
	while (<FILE>)			{
			$COUNT = $COUNT + 1 ;  my $COUNT_THOUSAND = $COUNT/10 ;
			my $COUNT_INTEGER = int($COUNT_THOUSAND) ; my $INTEGER_CHECK = 
$COUNT_THOUSAND - $COUNT_INTEGER;
#			if ($INTEGER_CHECK == 0) {print "Processing $COUNT\n";	}

			my $LINE = $_; chomp ($LINE);

			if ($LINE =~ /Round/)		{
				print "\tProcessing round $LINE\n" ;
				my $END_OF_ROUND_FOUND = 0 ;
				my %HASH_NT_BY_POSITION ; my $LONGEST_READ = 0 ;
				my $CONSENSUS = "" ;
				while ($END_OF_ROUND_FOUND < 1) {
					my $READ_LINE = <FILE> ; chomp($READ_LINE) ;
					if ($READ_LINE =~ /End of round/)	{
						$END_OF_ROUND_FOUND = 2 ;

	}
					if ($READ_LINE !~ /End of Round/)	{
						my @ARRAY_READ_LINE = split('\t',$READ_LINE) ;
						my $READ_NAME = $ARRAY_READ_LINE[0] ;
						my $READ_SEQ = $ARRAY_READ_LINE[1] ;
						my $READ_SEQ_LENGTH = length($READ_SEQ) ;
						if ($READ_SEQ_LENGTH > $LONGEST_READ)	{
							$LONGEST_READ = $READ_SEQ_LENGTH ;

			}
						my @ARRAY_NT = split('',$READ_SEQ) ;
						my $NUMBER_NT = scalar(@ARRAY_NT) ;
						my $COUNTER_NT = 0 ;
						while ($COUNTER_NT < $NUMBER_NT)	{
							my $CURRENT_NT = $ARRAY_NT[$COUNTER_NT] ;
							my $HASH_NT_UPDATE = 
$HASH_NT_BY_POSITION{$COUNTER_NT} ;
							$HASH_NT_UPDATE = 
$HASH_NT_UPDATE.="$CURRENT_NT" ;
							$HASH_NT_BY_POSITION{$COUNTER_NT} = 
$HASH_NT_UPDATE ;
							$COUNTER_NT = $COUNTER_NT + 1 ;

		}

	}

											}
				my $COUNTER_HASH_NT_TALLY = 0 ;
				my $A_TALLY = "" ; my $C_TALLY = "" ; my $G_TALLY = "" ; my 
$T_TALLY = "" ;

				while ($COUNTER_HASH_NT_TALLY < $LONGEST_READ)	{
```

```
                                    $HASH_INFO_CURRENT_NT_POSITION =
$HASH_NT_BY_POSITION{$COUNTER_HASH_NT_TALLY} ;
                                    $A_TALLY = $HASH_INFO_CURRENT_NT_POSITION =~ tr/A// ;
                                    $C_TALLY = $HASH_INFO_CURRENT_NT_POSITION =~ tr/C// ;
                                    $G_TALLY = $HASH_INFO_CURRENT_NT_POSITION =~ tr/G// ;
                                    $T_TALLY = $HASH_INFO_CURRENT_NT_POSITION =~ tr/T// ;
                                    my $CONSENSUS_NT_FOUND = 0 ; $CONSENSUS_NT = N ;
                                    if ($A_TALLY > $C_TALLY and $A_TALLY > $G_TALLY and
$A_TALLY > $T_TALLY)   {
                                            $CONSENSUS_NT_FOUND = 2 ;
                                            $CONSENSUS_NT = "A" ;

                                                                                    }
                                    if ($C_TALLY > $A_TALLY and $C_TALLY > $G_TALLY and
$C_TALLY > $T_TALLY)   {
                                            $CONSENSUS_NT_FOUND = 2 ;
                                            $CONSENSUS_NT = "C" ;

                                                                                    }
                                    if ($G_TALLY > $A_TALLY and $G_TALLY > $C_TALLY and
$G_TALLY > $T_TALLY)   {
                                            $CONSENSUS_NT_FOUND = 2 ;
                                            $CONSENSUS_NT = "G" ;

                                                                                    }
                                    if ($T_TALLY > $A_TALLY and $T_TALLY > $C_TALLY and
$T_TALLY > $G_TALLY)   {
                                            $CONSENSUS_NT_FOUND = 2 ;
                                            $CONSENSUS_NT = "T" ;

                                                                                    }
                                    $CONSENSUS = $CONSENSUS."$CONSENSUS_NT" ;

                                    $COUNTER_HASH_NT_TALLY = $COUNTER_HASH_NT_TALLY + 1 ;

                        }
                              $PRE_OUTPUT = $PRE_OUTPUT.="\>Consensus $LINE,$CONSENSUS\n" ;

                                                                            }

                                            }

        print "Creating output\n" ;

        my @ARRAY_PRE_OUTPUT = split('\n',$PRE_OUTPUT) ;
        my $NUMBER_PRE_OUTPUT_ELEMENTS = scalar(@ARRAY_PRE_OUTPUT) ;
        my $COUNTER_OUTPUT = $NUMBER_PRE_OUTPUT_ELEMENTS - 1 ;
        while ($COUNTER_OUTPUT >= 0)  {
                my $CURRENT_PRE_OUTPUT_ELEMENT = $ARRAY_PRE_OUTPUT[$COUNTER_OUTPUT] ;
                my @ARRAY_PRE_OUTPUT_ELEMENT = split(',',$CURRENT_PRE_OUTPUT_ELEMENT) ;
                my $CONSENSUS_NAME = $ARRAY_PRE_OUTPUT_ELEMENT[0] ;
                my $CONSENSUS_SEQ = $ARRAY_PRE_OUTPUT_ELEMENT[1] ;
                $OUTPUT = $OUTPUT.="$CONSENSUS_NAME\n$CONSENSUS_SEQ\n" ;
                $COUNTER_OUTPUT = $COUNTER_OUTPUT - 1 ;
                                                                    }


        #open (OUTPUT_SUMMARY, ">OUTPUT_SUMMARY_Contig_assembly_from_outputs.txt") or die "Can't
open $file for reading" ;
        #print OUTPUT_SUMMARY "$OUTPUT_SUMMARY" ;
        #close (OUTPUT_SUMMARY);



        open (OUTPUT, ">OUTPUT_nucleotide_consensus_upstream $CONTIG_NAME\_A.fasta") or die
"Can't open $file for reading" ;
        print OUTPUT "$OUTPUT" ;
        close (OUTPUT);

        #open (OUTPUT_TEST, ">OUTPUT_TEST_contig nucleotide consensus.txt") or die "Can't open
$file for reading" ;
```

```
#print OUTPUT_TEST "$PRE_OUTPUT" ;
#close (OUTPUT_TEST);


                              }
```

D.3_Contig_consensus_from_Downstream_building_outputs

```
$NAME_OF_MASTER_FILE = "Master_file_input_name.txt" ;

open (MASTER, "$NAME_OF_MASTER_FILE") or die "Can't open $file for reading" ;
while (<MASTER>) {
	my $LINE_MASTER_FILE = $_ ; chomp($LINE_MASTER_FILE) ;
	my $INPUT_FILE = $LINE_MASTER_FILE ;
	my @ARRAY_INPUT_FILE = split('\.',$INPUT_FILE) ;
	my	$NAME_OF_FILE = $ARRAY_INPUT_FILE[0] ;

	my @ARRAY_NAME_OF_FILE = split(' ',$NAME_OF_FILE) ;
	my $CONTIG_NAME = $ARRAY_NAME_OF_FILE[1] ;

	my $PRE_OUTPUT = "" ; my $OUTPUT = "" ; my $OUTPUT_TEST = "" ;
	my $CONTIG_CONSENSUS = "" ;   my $OUTPUT_SUMMARY = "" ;

	print "Processing file $NAME_OF_FILE, assessing nucleotide consensus by position\n" ;
	my $COUNT = 0 ;
	open (FILE, "$INPUT_FILE") or print "Can't open $file for reading\n" ;
	while (<FILE>)			{
		$COUNT = $COUNT + 1 ;  my $COUNT_THOUSAND = $COUNT/10 ;
		my $COUNT_INTEGER = int($COUNT_THOUSAND) ; my $INTEGER_CHECK = $COUNT_THOUSAND -
$COUNT_INTEGER;
#		if ($INTEGER_CHECK == 0) {print "Processing $COUNT\n";	}

		my $LINE = $_; chomp ($LINE);

		if ($LINE =~ /Round/)		{
			print "\tProcessing round $LINE\n" ;
			my $END_OF_ROUND_FOUND = 0 ;
			my %HASH_NT_BY_POSITION ; my $LONGEST_READ = 0 ;
			my $CONSENSUS = "" ;
			while ($END_OF_ROUND_FOUND < 1) {
				my $READ_LINE = <FILE> ; chomp($READ_LINE) ;
				if ($READ_LINE =~ /End of round/)	{
					$END_OF_ROUND_FOUND = 2 ;

	}
				if ($READ_LINE !~ /End of Round/)	{
					my @ARRAY_READ_LINE = split('\t',$READ_LINE) ;
					my $READ_NAME = $ARRAY_READ_LINE[0] ;
					my $READ_SEQ = $ARRAY_READ_LINE[1] ;
					my $READ_SEQ_LENGTH = length($READ_SEQ) ;
					if ($READ_SEQ_LENGTH > $LONGEST_READ)		{
						$LONGEST_READ = $READ_SEQ_LENGTH ;

		}
					my @ARRAY_NT = split('',$READ_SEQ) ;
					my $NUMBER_NT = scalar(@ARRAY_NT) ;
					my $COUNTER_NT = 0 ;
					while ($COUNTER_NT < $NUMBER_NT)	{
						my $CURRENT_NT = $ARRAY_NT[$COUNTER_NT] ;
						my $HASH_NT_UPDATE =
$HASH_NT_BY_POSITION{$COUNTER_NT} ;
						$HASH_NT_UPDATE = $HASH_NT_UPDATE.="$CURRENT_NT" ;
						$HASH_NT_BY_POSITION{$COUNTER_NT} = $HASH_NT_UPDATE
;
						$COUNTER_NT = $COUNTER_NT + 1 ;

	}

	}

											}
			my $COUNTER_HASH_NT_TALLY = 0 ;
			my $A_TALLY = "" ; my $C_TALLY = "" ; my $G_TALLY = "" ; my $T_TALLY = ""
;

			while ($COUNTER_HASH_NT_TALLY < $LONGEST_READ)		{
```

```
                              $HASH_INFO_CURRENT_NT_POSITION =
$HASH_NT_BY_POSITION{$COUNTER_HASH_NT_TALLY} ;
                              $A_TALLY = $HASH_INFO_CURRENT_NT_POSITION =~ tr/A// ;
                              $C_TALLY = $HASH_INFO_CURRENT_NT_POSITION =~ tr/C// ;
                              $G_TALLY = $HASH_INFO_CURRENT_NT_POSITION =~ tr/G// ;
                              $T_TALLY = $HASH_INFO_CURRENT_NT_POSITION =~ tr/T// ;
                              my $CONSENSUS_NT_FOUND = 0 ; $CONSENSUS_NT = N ;
                              if ($A_TALLY > $C_TALLY and $A_TALLY > $G_TALLY and $A_TALLY >
$T_TALLY)      {
                                     $CONSENSUS_NT_FOUND = 2 ;
                                     $CONSENSUS_NT = "A" ;

                                                                          }
                              if ($C_TALLY > $A_TALLY and $C_TALLY > $G_TALLY and $C_TALLY >
$T_TALLY)      {
                                     $CONSENSUS_NT_FOUND = 2 ;
                                     $CONSENSUS_NT = "C" ;

                                                                          }
                              if ($G_TALLY > $A_TALLY and $G_TALLY > $C_TALLY and $G_TALLY >
$T_TALLY)      {
                                     $CONSENSUS_NT_FOUND = 2 ;
                                     $CONSENSUS_NT = "G" ;

                                                                          }
                              if ($T_TALLY > $A_TALLY and $T_TALLY > $C_TALLY and $T_TALLY >
$G_TALLY)      {
                                     $CONSENSUS_NT_FOUND = 2 ;
                                     $CONSENSUS_NT = "T" ;

                                                                          }
                              $CONSENSUS = $CONSENSUS."$CONSENSUS_NT" ;

                              $OUTPUT_TEST = $OUTPUT_TEST."$LINE\n" ;
                              $OUTPUT_TEST = $OUTPUT_TEST."Position
$COUNTER_HASH_NT_TALLY\tA:$A_TALLY\tC:$C_TALLY\t" ;
                              $OUTPUT_TEST = $OUTPUT_TEST."G:$G_TALLY\tT:$T_TALLY\n" ;

                              $COUNTER_HASH_NT_TALLY = $COUNTER_HASH_NT_TALLY + 1 ;

               }
                       $OUTPUT = $OUTPUT.="\>Consensus $LINE\n$CONSENSUS\n" ;

                                                                  }

                                     }

       #open (OUTPUT_SUMMARY, ">OUTPUT_SUMMARY_Contig_assembly_from_outputs.txt") or die "Can't
open $file for reading" ;
       #print OUTPUT_SUMMARY "$OUTPUT_SUMMARY" ;
       #close (OUTPUT_SUMMARY);



       open (OUTPUT, ">OUTPUT_nucleotide_consensus_downstream $CONTIG_NAME\_A.fasta") or die
"Can't open $file for reading" ;
       print OUTPUT "$OUTPUT" ;
       close (OUTPUT);

       #open (OUTPUT_TEST, ">OUTPUT_TEST_contig nucleotide consensus.txt") or die "Can't open
$file for reading" ;
       #print OUTPUT_TEST "$OUTPUT_TEST" ;
       #close (OUTPUT_TEST);

                                             }
```

D.4_Contig_assembly_consensus_from_building_outputs_with_N_check

```
$NAME_OF_MASTER_FILE = "Master_input_file_name.txt" ;
open (MASTER, "$NAME_OF_MASTER_FILE") or die "Can't open $file for reading" ;

$SUBFILE_DESIGNATOR = "Z" ; #TO UPDATE ###

$CONSENSUS_SUBFILES_TO_REASSEMBLE = "" ;
$MASTER_FILE_ALIGN_FILES = "" ;

while (<MASTER>) {
        my $LINE_MASTER_FILE = $_ ; chomp($LINE_MASTER_FILE) ;
        my $INPUT_FILE = $LINE_MASTER_FILE ;
        my @ARRAY_INPUT_FILE = split('\.',$INPUT_FILE) ;
        my $NAME_OF_FILE = $ARRAY_INPUT_FILE[0] ;

        my @ARRAY_NAME_OF_FILE = split(' ',$NAME_OF_FILE) ;
        my $FILE_TYPE = $ARRAY_NAME_OF_FILE[0] ;
        my $CONTIG_NAME_FULL = $ARRAY_NAME_OF_FILE[1] ;
        my @ARRAY_CONTIG_NAME_FULL = split('\_',$CONTIG_NAME_FULL) ;

        my $CONTIG_SERIES_DESIGNATOR_1 = $ARRAY_CONTIG_NAME_FULL[0] ;
        my $CONTIG_SERIES_DESIGNATOR_2 = $ARRAY_CONTIG_NAME_FULL[1] ;
        my $CONTIG_NUMBER_DESIGNATOR = $ARRAY_CONTIG_NAME_FULL[2] ;
        my $CONTIG_SUBASSEMBLY_DESIGNATOR = $ARRAY_CONTIG_NAME_FULL[3] ;

        my $CONTIG_NAME =
$CONTIG_NAME.="$CONTIG_SERIES_DESIGNATOR_1\_$CONTIG_SERIES_DESIGNATOR_2\_$CONTIG_NUMBER_DESIGNATO
R" ;

        my $PRE_OUTPUT = "" ; my $OUTPUT = "" ; my $OUTPUT_TEST = "" ;
        my $CONTIG_CONSENSUS = "" ;   my $OUTPUT_SUMMARY = "" ;
        my $OUTPUT_ALIGNMENT = "" ; my $ALIGNMENT = "" ;
        my $OUTPUT_SUMMARY = "Contig Name\tMultiple Assembly\tLength 1st consensus\tMulti-
assembly threshold\tTotal number consensus\n" ;

        my $MULTIPLE_ASSEMBLIES_CHECK = 0 ;
        my $SEQUENCE_START_OF_NEW_ASSEMBLY = 0 ;
        my $CONSENSUS_MAX_LENGTH = 0 ;
        my $OTHER_SEQ_SET_TO_ASSEMBLE = "" ;

        print "Processing file $NAME_OF_FILE, assembly from consensus\n" ;
        my $COUNT = 0 ;
        open (FILE, "$INPUT_FILE") or print "Can't open $file for reading\n" ;

        while (<FILE>)        {
                $COUNT = $COUNT + 1 ;  my $COUNT_THOUSAND = $COUNT/10 ;
                my $COUNT_INTEGER = int($COUNT_THOUSAND) ; my $INTEGER_CHECK = $COUNT_THOUSAND -
$COUNT_INTEGER;
                if ($INTEGER_CHECK == 0) {print "Adding sequence $COUNT to consensus\n";   }
        #       print "\tProcessing line $COUNT\n" ;
                my $LINE = $_; chomp ($LINE);
                if ($LINE =~ />/)      {
                        my $NAME = $LINE ;
                        my $SEQUENCE = <FILE> ; chomp($SEQUENCE) ;
                        my $LENGTH_SEQ = length($SEQUENCE) ;
                        my $CONSENSUS_LENGTH = length($CONTIG_CONSENSUS) ;
                        if ($CONSENSUS_LENGTH > 5)           {
                        print "\t\tconsensus sequence length is $CONSENSUS_LENGTH\n" ;
                        my $LENGTH_SHARED_NT_MOTIF = 30 ; my $SHARED_NT_MOTIF_FOUND = 0 ;
                        my $SHARED_NT_SEQ = "" ;

                        my $COUNTER_FOR_CONSENSUS = 0 ;
                        my $LENGTH_CONSENSUS_SCREENED = $LENGTH_SHARED_NT_MOTIF ;
                        my $MAX_LENGTH_CONSENSUS_TO_SCREEN = $CONSENSUS_LENGTH -
$LENGTH_SHARED_NT_MOTIF ;

                        while ($SHARED_NT_MOTIF_FOUND < 1 and $LENGTH_CONSENSUS_SCREENED <
$MAX_LENGTH_CONSENSUS_TO_SCREEN)      {
                                my $CONSENSUS_REGION_TO_SEARCH =
substr($CONTIG_CONSENSUS,$COUNTER_FOR_CONSENSUS,$LENGTH_SHARED_NT_MOTIF) ;
```

```
                              if ($SEQUENCE =~ /$CONSENSUS_REGION_TO_SEARCH/)      {
                                     $SHARED_NT_MOTIF_FOUND = 2 ;
                                     $SHARED_NT_SEQ = $SHARED_NT_SEQ
.="$CONSENSUS_REGION_TO_SEARCH"  ;
                                     $START_POSITION_FOR_CONSENSUS = $COUNTER_FOR_CONSENSUS ;

                              }
                              $LENGTH_CONSENSUS_SCREENED = $LENGTH_CONSENSUS_SCREENED + 1 ;

                              $COUNTER_FOR_CONSENSUS = $COUNTER_FOR_CONSENSUS + 1 ;


                       }
                       $OUTPUT_TEST = $OUTPUT_TEST.="Sequence $COUNT\t Shared
nt:$SHARED_NT_SEQ\n" ;

                       my $START_POSITION_FOR_SEQ = 0 ; my $SHARED_NT_MOTIF_FOUND_2 = 0 ;
                       my $COUNTER_FOR_SEQ = 0 ;
                       my $LENGTH_SEQ_SCREENED = $LENGTH_SHARED_NT_MOTIF ;
                       my $MAX_LENGTH_SEQ_TO_SCREEN = $LENGTH_SEQ - $LENGTH_SHARED_NT_MOTIF ;

                       while ($SHARED_NT_MOTIF_FOUND_2 < 1 and $LENGTH_SEQ_SCREENED <
$MAX_LENGTH_SEQ_TO_SCREEN)    {
                              my $SEQ_REGION_TO_SEARCH =
substr($SEQUENCE,$COUNTER_FOR_SEQ,$LENGTH_SHARED_NT_MOTIF) ;
                              if ($SEQ_REGION_TO_SEARCH eq $SHARED_NT_SEQ)         {
                                     $SHARED_NT_MOTIF_FOUND_2 = 2 ;
                                     $START_POSITION_FOR_SEQ = $COUNTER_FOR_SEQ ;

                       }
                              $COUNTER_FOR_SEQ = $COUNTER_FOR_SEQ + 1 ;
                              $LENGTH_SEQ_SCREENED = $LENGTH_SEQ_SCREENED + 1 ;


       }
                       my $UPSTREAM_POSITION_OF_SEQ_TO_CONSENSUS = $START_POSITION_FOR_CONSENSUS
- $START_POSITION_FOR_SEQ  ;
                       my $NUMBER_DASHES_TO_ADD = $UPSTREAM_POSITION_OF_SEQ_TO_CONSENSUS ;
                       my $COUNTER_DASHES = 0 ; my $DASHES_TO_ADD = "" ;
                       while ($COUNTER_DASHES < $NUMBER_DASHES_TO_ADD)      {
                              $DASHES_TO_ADD = $DASHES_TO_ADD.="\-" ;
                              $COUNTER_DASHES = $COUNTER_DASHES + 1 ;

                       }

                       my $DISTANCE_CONSENSUS_DOWNSTREAM = $CONSENSUS_LENGTH -
$START_POSITION_FOR_CONSENSUS ;
                       my $START_POSITION_SEQ_TO_ADD_TO_CONSENSUS = $START_POSITION_FOR_SEQ +
$DISTANCE_CONSENSUS_DOWNSTREAM ;
                       my $LENGTH_SEQ_DOWNSTREAM_TO_ADD_TO_CONSENSUS = $LENGTH_SEQ -
$START_POSITION_SEQ_TO_ADD_TO_CONSENSUS ;
                       my $SEQ_DOWNSTREAM_TO_ADD_TO_CONSENSUS =
substr($SEQUENCE,$START_POSITION_SEQ_TO_ADD_TO_CONSENSUS,$LENGTH_SEQ_DOWNSTREAM_TO_ADD_TO_CONSENS
US) ;
                       my $SEQ_TO_ADD_TO_CONSENSUS_N_CHECKED = "" ;
                       if ($SEQ_DOWNSTREAM_TO_ADD_TO_CONSENSUS !~ /N/) {
                              $SEQ_TO_ADD_TO_CONSENSUS_N_CHECKED =
$SEQ_DOWNSTREAM_TO_ADD_TO_CONSENSUS ;

               }
                       if ($SEQ_DOWNSTREAM_TO_ADD_TO_CONSENSUS =~ /N/) {
                              my $LENGTH_FOR_N_SEARCH = 1 ; my $N_FOUND = 0 ;
                              while ($N_FOUND < 1)   {
                                     my $REGION_BEING_SCREENED =
substr($SEQ_DOWNSTREAM_TO_ADD_TO_CONSENSUS,0,$LENGTH_FOR_N_SEARCH) ;
                                     if ($REGION_BEING_SCREENED =~ /N/)           {
                                            $N_FOUND = 2 ;
                                            my $LENGTH_WITHOUT_N = $LENGTH_FOR_N_SEARCH - 1 ;
                                            $SEQ_TO_ADD_TO_CONSENSUS_N_CHECKED =
substr($SEQ_DOWNSTREAM_TO_ADD_TO_CONSENSUS,0,$LENGTH_WITHOUT_N) ;
```

```
                }
                                        $LENGTH_FOR_N_SEARCH = $LENGTH_FOR_N_SEARCH + 1 ;
                                                                              }

                }
                        $CONTIG_CONSENSUS =
$CONTIG_CONSENSUS.="$SEQ_TO_ADD_TO_CONSENSUS_N_CHECKED" ;

                        $OUTPUT_ALIGNMENT = $OUTPUT_ALIGNMENT.="Consensus
start:$START_POSITION_FOR_CONSENSUS" ;
                        $OUTPUT_ALIGNMENT = $OUTPUT_ALIGNMENT.="\tSequence start
position:$START_POSITION_FOR_SEQ\n" ;
                        $OUTPUT_ALIGNMENT =
$OUTPUT_ALIGNMENT.="$CONTIG_CONSENSUS\n$DASHES_TO_ADD$SEQUENCE\n" ;

                                                                                              }
                        if ($CONSENSUS_LENGTH < 5)      {
                                print "\t\tconsensus sequence length is $CONSENSUS_LENGTH\n" ;
                                $CONTIG_CONSENSUS = $CONTIG_CONSENSUS.="$SEQUENCE" ;
                                                                        }
                        my $UPDATED_CONSENSUS_LENGTH = length($CONTIG_CONSENSUS) ;


                        if ($UPDATED_CONSENSUS_LENGTH > 600)  {
                                if ($CONSENSUS_MAX_LENGTH == $UPDATED_CONSENSUS_LENGTH and
$MULTIPLE_ASSEMBLIES_CHECK < 1)         {
                                        $MULTIPLE_ASSEMBLIES_CHECK = 2 ;  ;
                                        $SEQUENCE_START_OF_NEW_ASSEMBLY = $COUNT ;


        }


                                if ($CONSENSUS_MAX_LENGTH < $UPDATED_CONSENSUS_LENGTH)      {
#                               $OUTPUT_SUMMARY =
$OUTPUT_SUMMARY.="$CONSENSUS_MAX_LENGTH\t$UPDATED_CONSENSUS_LENGTH\n" ;    #
                                        $CONSENSUS_MAX_LENGTH = $UPDATED_CONSENSUS_LENGTH ;
#                               $OUTPUT_SUMMARY = $OUTPUT_SUMMARY.="Consensus Max length smaller
than updated, consensus $COUNT\n" ;#

                                        }

        }
                        if ($MULTIPLE_ASSEMBLIES_CHECK > 1) {
                                $OTHER_SEQ_SET_TO_ASSEMBLE =
$OTHER_SEQ_SET_TO_ASSEMBLE.="$NAME\n$SEQUENCE\n" ;
                                                                                              }

                                                }
        if ($MULTIPLE_ASSEMBLIES_CHECK < 1) { $MULTIPLE_ASSEMBLIES = "N";}
        if ($MULTIPLE_ASSEMBLIES_CHECK > 1) { $MULTIPLE_ASSEMBLIES = "Y";}

        $OUTPUT_SUMMARY =
$OUTPUT_SUMMARY.="$CONTIG_NAME\t$MULTIPLE_ASSEMBLIES\t$CONSENSUS_MAX_LENGTH\t" ;
        $OUTPUT_SUMMARY = $OUTPUT_SUMMARY.="$SEQUENCE_START_OF_NEW_ASSEMBLY\t$COUNT\n" ;


                                                        }
#       my $OUTPUT_SUMMARY = "Contig Name\tMultiple Assembly\tMulti-assembly threshold\tTotal
number consensus\n" ;

        my @ARRAY_OTHER_SEQ_SET_TO_ASSEMBLE = split('\n',$OTHER_SEQ_SET_TO_ASSEMBLE) ;
        my $NUMBER_LINE_OTHER_SEQ_SET_TO_ASSEMBLE = scalar(@ARRAY_OTHER_SEQ_SET_TO_ASSEMBLE) ;

        if ($NUMBER_LINE_OTHER_SEQ_SET_TO_ASSEMBLE > 10) {
                my $NAME_REASSEMBLY_FILE =
"$CONTIG_SERIES_DESIGNATOR_1\_$CONTIG_SERIES_DESIGNATOR_2\_" ;
                $NAME_REASSEMBLY_FILE =
$NAME_REASSEMBLY_FILE.="$CONTIG_NUMBER_DESIGNATOR\_$SUBFILE_DESIGNATOR.fasta" ;
```

```
                open (OUTPUT_MULTI_ASSEMBLIES, ">$FILE_TYPE $NAME_REASSEMBLY_FILE") or die "Can't
open $file for reading" ;
                print OUTPUT_MULTI_ASSEMBLIES "$OTHER_SEQ_SET_TO_ASSEMBLE" ;

                close (OUTPUT_MULTI_ASSEMBLIES);
                $CONSENSUS_SUBFILES_TO_REASSEMBLE = $CONSENSUS_SUBFILES_TO_REASSEMBLE.="$FILE_TYPE
$NAME_REASSEMBLY_FILE\n" ;

        }
        close (FILE) ;


        open (OUTPUT_ALIGN, ">assembly_$NAME_OF_FILE.align") or die "Can't open $file for
reading" ;
        print OUTPUT_ALIGN "$OUTPUT_ALIGNMENT" ;
        close (OUTPUT_ALIGN);


        $MASTER_FILE_ALIGN_FILES = $MASTER_FILE_ALIGN_FILES.="assembly_$NAME_OF_FILE.align\n" ;

        open (OUTPUT_ALIGN_SUMMARY, ">summary_assembly_$NAME_OF_FILE.txt") or die "Can't open
$file for reading" ;
        print OUTPUT_ALIGN_SUMMARY "$OUTPUT_SUMMARY" ;
        close (OUTPUT_ALIGN_SUMMARY);

        #open (OUTPUT_TEST, ">OUTPUT_TEST_contig assembly.txt") or die "Can't open $file for
reading" ;
        #print OUTPUT_TEST "$OUTPUT_TEST" ;
        #close (OUTPUT_TEST);
                                      }

        $REASSEMBLE_SIZE_CHECK = length($CONSENSUS_SUBFILES_TO_REASSEMBLE) ;
        if ($REASSEMBLE_SIZE_CHECK > 5) {
                open (OUTPUT_RE_ASSEMBLE, ">To_reassemble_set $SUBFILE_DESIGNATOR
$NAME_OF_MASTER_FILE") or die "Can't open $file for reading" ;
                print OUTPUT_RE_ASSEMBLE "$CONSENSUS_SUBFILES_TO_REASSEMBLE" ;

                close (OUTPUT_RE_ASSEMBLE);
                                                                    }

        open (OUTPUT_MASTER_ALIGN, ">Master file for names of align files from
$NAME_OF_MASTER_FILE") or die "Can't open $file for reading" ;
        print OUTPUT_MASTER_ALIGN "$MASTER_FILE_ALIGN_FILES" ;

        close (OUTPUT_MASTER_ALIGN);
```

D.5_Contig_consolidating_assembled_consensus

```
$NAME_OF_MASTER_FILE = "Master_input_file.txt" ;
open (MASTER, "$NAME_OF_MASTER_FILE") or die "Can't open $file for reading" ;

@ARRAY_NAME_OF_MASTER_FILE = split('\.',$NAME_OF_MASTER_FILE) ;
$NAME_OF_FILE_FOR_OUTPUT = $ARRAY_NAME_OF_MASTER_FILE[0] ;

$OUTPUT = "" ; $OUTPUT_TEST = "" ;
$OUTPUT_UPS_DOWNS = "" ;
$LIST_CONTIGS_NAMES = "" ;
$OUTPUT_ACCNOS = "" ;

%HASH_DOWNSTREAM_ALIGN ; %HASH_UPSTREAM_ALIGN ;
print "Retrieving fully assembled sequences from align files\n" ;
while (<MASTER>)      {
       my $LINE_MASTER_FILE = $_ ; chomp($LINE_MASTER_FILE) ;
       my $INPUT_FILE = $LINE_MASTER_FILE ;
       my @ARRAY_INPUT_FILE = split('\.',$INPUT_FILE) ;
       my $NAME_OF_FILE = $ARRAY_INPUT_FILE[0] ;

       my $DOWNSTREAM_FILE = 0 ; my $UPSTREAM_FILE = 0 ;
       if ($NAME_OF_FILE =~ /downstream/)    {
              $DOWNSTREAM_FILE = 2 ;
                                                                      }
       if ($NAME_OF_FILE =~ /upstream/)      {
              $UPSTREAM_FILE = 2 ;
                                                                      }

       my @ARRAY_NAME_OF_FILE = split(' ',$NAME_OF_FILE) ;
       my $CONTIG_NAME = $ARRAY_NAME_OF_FILE[1] ;
       if ($LIST_CONTIGS_NAMES !~ /$CONTIG_NAME,/)  {
              $LIST_CONTIGS_NAMES = $LIST_CONTIGS_NAMES.="$CONTIG_NAME," ;
                                                                                     }

       print "Processing file $NAME_OF_FILE\n" ;
       my $COUNT = 0 ; my $NUMBER_CONDENSED_CONSENSUS = 0 ;
       my %HASH_CONDENSED_CONSENSUS ;
       open (FILE, "$INPUT_FILE") or print "Can't open $file for reading\n" ;
       while (<FILE>)              {
              $COUNT = $COUNT + 1 ;  my $COUNT_THOUSAND = $COUNT/10 ;
              my $COUNT_INTEGER = int($COUNT_THOUSAND) ; my $INTEGER_CHECK = $COUNT_THOUSAND -
$COUNT_INTEGER;
              if ($INTEGER_CHECK == 0) {print "Adding sequence $COUNT to consensus\n";   }
       #      print "\tProcessing line $COUNT\n" ;
              my $LINE = $_; chomp ($LINE);
              if ($LINE =~ /Consensus/)     {
                     my $CONDENSED_CONSENSUS = <FILE> ; chomp($CONDENSED_CONSENSUS) ;
                     my $ALIGNMENT_LAST_READ = <FILE> ; chomp($ALIGNMENT_LAST_READ) ;
                     $NUMBER_CONDENSED_CONSENSUS = $NUMBER_CONDENSED_CONSENSUS + 1 ;
                     $HASH_CONDENSED_CONSENSUS{$NUMBER_CONDENSED_CONSENSUS} =
$CONDENSED_CONSENSUS ;
                                                                 }
                                          }
       close (FILE) ;
       my $CONDENSED_CONSENSUS_TO_KEEP = $HASH_CONDENSED_CONSENSUS{$NUMBER_CONDENSED_CONSENSUS}
;

       if ($DOWNSTREAM_FILE > 1)     {
              $HASH_DOWNSTREAM_ALIGN{$CONTIG_NAME} = $CONDENSED_CONSENSUS_TO_KEEP ;
                                                          }
       if ($UPSTREAM_FILE > 1)       {
              $HASH_UPSTREAM_ALIGN{$CONTIG_NAME} = $CONDENSED_CONSENSUS_TO_KEEP ;
                                                          }
                              }

close (MASTER) ;

print "Preparing output\n" ;
@ARRAY_LIST_CONTIGS_NAMES = split(',',$LIST_CONTIGS_NAMES) ;
#@ARRAY_LIST_CONTIGS_NAMES_SORTED = sort {$a cmp $b} @ARRAY_LIST_CONTIGS_NAMES ;
```

```
$NUMBER_CONTIG_NAMES = scalar(@ARRAY_LIST_CONTIGS_NAMES) ;
#my $NUMBER_CONTIG_NAMES = scalar(@ARRAY_LIST_CONTIGS_NAMES_SORTED) ;
my $COUNTER_MERGE = 0 ;
while ($COUNTER_MERGE < $NUMBER_CONTIG_NAMES)        {
        my $CURRENT_CONTIG = $ARRAY_LIST_CONTIGS_NAMES[$COUNTER_MERGE] ;
#       my $CURRENT_CONTIG = $ARRAY_LIST_CONTIGS_NAMES_SORTED[$COUNTER_MERGE] ;

#       $OUTPUT_UPS_DOWNS = $OUTPUT_UPS_DOWNS.=">$CURRENT_CONTIG\t" ;
#       $OUTPUT_TEST = $OUTPUT_TEST.=">$CURRENT_CONTIG\n" ;

        my $UPSTREAM_SEQUENCE = $HASH_UPSTREAM_ALIGN{$CURRENT_CONTIG} ;
        my $SIZE_CHECK_UPSTREAM_SEQUENCE = length($UPSTREAM_SEQUENCE) ;

        my $DOWNSTREAM_SEQUENCE = $HASH_DOWNSTREAM_ALIGN{$CURRENT_CONTIG} ;
        my $SIZE_CHECK_DOWNSTREAM_SEQUENCE = length($DOWNSTREAM_SEQUENCE) ;

        if ($SIZE_CHECK_UPSTREAM_SEQUENCE > 5)       {
               $OUTPUT_UPS_DOWNS =
$OUTPUT_UPS_DOWNS.=">$CURRENT_CONTIG\tupstream\n$UPSTREAM_SEQUENCE\n" ;
               $OUTPUT_ACCNOS = $OUTPUT_ACCNOS.="$CURRENT_CONTIG\tupstream\n" ;
                                                                                  }

        if ($SIZE_CHECK_DOWNSTREAM_SEQUENCE > 5)     {
               $OUTPUT_UPS_DOWNS =
$OUTPUT_UPS_DOWNS.=">$CURRENT_CONTIG\tdownstream\n$DOWNSTREAM_SEQUENCE\n" ;
               $OUTPUT_ACCNOS = $OUTPUT_ACCNOS.="$CURRENT_CONTIG\tdownstream\n" ;
                                                                                          }
        $COUNTER_MERGE = $COUNTER_MERGE + 1 ;
                                                                                          }


open (OUTPUT_UPS_DOWNS, ">OUTPUT_upstream and downstream $NAME_OF_FILE_FOR_OUTPUT.fasta") or die
"Can't open $file for reading" ;
print OUTPUT_UPS_DOWNS "$OUTPUT_UPS_DOWNS" ;
close (OUTPUT_UPS_DOWNS) ;

open (OUTPUT_ACCNOS, ">OUTPUT_upstream and downstream $NAME_OF_FILE_FOR_OUTPUT.accnos") or die
"Can't open $file for reading" ;
print OUTPUT_ACCNOS "$OUTPUT_ACCNOS" ;
close (OUTPUT_ACCNOS) ;
```

D.6_Contig_merging_from_upstream_downstream.txt

```
$NAME_OF_FILE = "Name_input_file.fasta" ;

@ARRAY_NAME_OF_FILE = split(' ',$NAME_OF_FILE) ;
$NUMBER_ELEMENTS_NAME_OF_FILE = scalar(@ARRAY_NAME_OF_FILE) ;
$POSITION_FOR_LAST_ELEMENT_ARRAY_NAME_OF_FILE = $NUMBER_ELEMENTS_NAME_OF_FILE - 1 ;
$NAME_OF_FILE_FOR_OUTPUT = $ARRAY_NAME_OF_FILE[$POSITION_FOR_LAST_ELEMENT_ARRAY_NAME_OF_FILE] ;

$OUTPUT = "" ; $OUTPUT_TEST = "" ;
$OUTPUT_UPS_DOWNS = "" ;
%HASH_DOWNSTREAM_ALIGN ; %HASH_UPSTREAM_ALIGN ;

$CONTIG_PAIRS_TO_MERGE = "" ;
$OUTPUT_CONTIG_PAIRS_NOT_MERGED = "Contig pairs not merged:\n" ;
$CONTIGS_TO_OUTPUT_NO_MERGING = "" ; $CONTIGS_TO_OUTPUT_NO_MERGING_FOR_ACCNOS = "" ;
$LIST_CONTIG_SERIES = "" ;
$LIST_ALL_CONTIGS_NAMES = "" ;
%HASH_PRE_MERGED_CONTIGS ; %HASH_SUBASSEMBLY_DESIGNATORS_UPSTREAM ;
%HASH_SUBASSEMBLY_DESIGNATORS_DOWNSTREAM ;

$OUTPUT_OVERLAP_NT_IDENTITIES = "\t\% identity overlap\tDownstream start position\t\%identity
full ups-down regions\tfull overlap length\n" ;

print "Matching upstream and downstream contigs for merging\n" ;

$COUNT = 0 ; $NUMBER_CONDENSED_CONSENSUS = 0 ;
%HASH_CONDENSED_CONSENSUS ;
open (FILE, "$NAME_OF_FILE") or print "Can't open $file for reading\n" ;
while (<FILE>)          {
        $COUNT = $COUNT + 1 ;  my $COUNT_THOUSAND = $COUNT/10 ;
        my $COUNT_INTEGER = int($COUNT_THOUSAND) ; my $INTEGER_CHECK = $COUNT_THOUSAND -
$COUNT_INTEGER;
        if ($INTEGER_CHECK == 0) {print "\tProcessing line $COUNT\n" };
        my $LINE = $_; chomp ($LINE);
        if ($LINE =~ />/)      {
                my $CONTIG_NAME_LINE = $LINE  ;
                my @ARRAY_CONTIG_NAME_LINE = split('\t',$CONTIG_NAME_LINE) ;
                my $CONTIG_ORIENTATION = $ARRAY_CONTIG_NAME_LINE[1] ;
                my $CONTIG_NAME = $ARRAY_CONTIG_NAME_LINE[0] ;
                my $CONTIG_NAME_ADJUSTED = "" ;

                if ($CONTIG_ORIENTATION =~ /upstream/)       {
                        $CONTIG_NAME_ADJUSTED = "$CONTIG_NAME\_u" ;
                                                                                        }
                if ($CONTIG_ORIENTATION =~ /downstream/)     {
                        $CONTIG_NAME_ADJUSTED = "$CONTIG_NAME\_d" ;

        }

                $LIST_ALL_CONTIGS_NAMES = $LIST_ALL_CONTIGS_NAMES.="$CONTIG_NAME_ADJUSTED," ;

                my @ARRAY_CONTIG_NAME = split('\_',$CONTIG_NAME) ;
                my $CONTIG_SERIES_DESIGNATOR_1 = $ARRAY_CONTIG_NAME[0] ;
                my $CONTIG_SERIES_DESIGNATOR_2 = $ARRAY_CONTIG_NAME[1] ;
                my $CONTIG_NUMBER_DESIGNATOR = $ARRAY_CONTIG_NAME[2] ;
                my $CONTIG_SERIE =
"$CONTIG_SERIES_DESIGNATOR_1\_$CONTIG_SERIES_DESIGNATOR_2\_$CONTIG_NUMBER_DESIGNATOR" ;
#               my $CONTIG_SERIE = "$CONTIG_SERIES_DESIGNATOR_1\_$CONTIG_NUMBER_DESIGNATOR" ;
                if ($LIST_CONTIG_SERIES !~ /$CONTIG_SERIE/)  {
                        $LIST_CONTIG_SERIES = $LIST_CONTIG_SERIES.="$CONTIG_SERIE," ;
                                                                        }
                my $CONTIG_SUBASSEMBLY_DESIGNATOR = $ARRAY_CONTIG_NAME[3] ;
                if ($CONTIG_ORIENTATION =~ /upstream/)       {
                        my $UPDATE_HASH_SUBASSEMBLY_DESIGNATORS =
$HASH_SUBASSEMBLY_DESIGNATORS_UPSTREAM{$CONTIG_SERIE} ;
                        $UPDATE_HASH_SUBASSEMBLY_DESIGNATORS =
$UPDATE_HASH_SUBASSEMBLY_DESIGNATORS.="$CONTIG_SUBASSEMBLY_DESIGNATOR," ;
                        $HASH_SUBASSEMBLY_DESIGNATORS_UPSTREAM{$CONTIG_SERIE} =
$UPDATE_HASH_SUBASSEMBLY_DESIGNATORS ;
                                                                                        }
```

```
		if ($CONTIG_ORIENTATION =~ /downstream/)	{
			my $UPDATE_HASH_SUBASSEMBLY_DESIGNATORS_DOWNSTREAM =
$HASH_SUBASSEMBLY_DESIGNATORS_DOWNSTREAM{$CONTIG_SERIE} ;
			$UPDATE_HASH_SUBASSEMBLY_DESIGNATORS_DOWNSTREAM =
$UPDATE_HASH_SUBASSEMBLY_DESIGNATORS_DOWNSTREAM.="$CONTIG_SUBASSEMBLY_DESIGNATOR," ;
			$HASH_SUBASSEMBLY_DESIGNATORS_DOWNSTREAM{$CONTIG_SERIE} =
$UPDATE_HASH_SUBASSEMBLY_DESIGNATORS_DOWNSTREAM ;
											}
		my $CONTIG_SEQ_LINE = <FILE> ; chomp($CONTIG_SEQ_LINE) ;

		$HASH_PRE_MERGED_CONTIGS{$CONTIG_NAME_ADJUSTED} = $CONTIG_SEQ_LINE ;

						}
					}
close (FILE) ;
print "Sorting contigs for merging or for strait output\n" ;
#$OUTPUT_TEST = $OUTPUT_TEST.="$LIST_CONTIG_SERIES\n" ;
@ARRAY_LIST_CONTIG_SERIES = split(',',$LIST_CONTIG_SERIES) ;
$NUMBER_ELEMENTS_CONTIGS_SERIES = scalar(@ARRAY_LIST_CONTIG_SERIES) ;
#$OUTPUT_TEST = $OUTPUT_TEST.="$NUMBER_ELEMENTS_CONTIGS_SERIES\n" ;
$COUNTER_CONTIG_SERIES = 0 ;
while ($COUNTER_CONTIG_SERIES < $NUMBER_ELEMENTS_CONTIGS_SERIES)	{
	my $CURRENT_ELEMENT_CONTIGS_SERIES = $ARRAY_LIST_CONTIG_SERIES[$COUNTER_CONTIG_SERIES] ;

	my $HASH_SUBASSEMBLY_INFO_FOR_CURRENT_ELEMENT_CONTIG_1 =
$HASH_SUBASSEMBLY_DESIGNATORS_UPSTREAM{$CURRENT_ELEMENT_CONTIGS_SERIES} ;
#	$OUTPUT_TEST = $OUTPUT_TEST.="$HASH_SUBASSEMBLY_INFO_FOR_CURRENT_ELEMENT_CONTIG_1\n\n" ;
#	$OUTPUT_TEST =
$OUTPUT_TEST.="$CURRENT_ELEMENT_CONTIGS_SERIES\t$HASH_SUBASSEMBLY_INFO_FOR_CURRENT_ELEMENT_CONTIG
\t" ;
	my @ARRAY_HASH_SUBASSEMBLY_INFO_1 =
split(',',$HASH_SUBASSEMBLY_INFO_FOR_CURRENT_ELEMENT_CONTIG_1) ;
	sort(@ARRAY_HASH_SUBASSEMBLY_INFO_1) ;
#	$OUTPUT_TEST = $OUTPUT_TEST.="$HASH_SUBASSEMBLY_INFO_FOR_CURRENT_ELEMENT_CONTIG_1\n" ;
	$NUMBER_ELEMENTS_SUBASSEMBLY_UPSTREAM = scalar(@ARRAY_HASH_SUBASSEMBLY_INFO_1) ;

	$ARRAY_POSITION_TO_MERGE_UPSTREAM = $NUMBER_ELEMENTS_SUBASSEMBLY_UPSTREAM - 1 ;
	$ARRAY_ELEMENT_TO_MERGE_UPSTREAM =
$ARRAY_HASH_SUBASSEMBLY_INFO_1[$ARRAY_POSITION_TO_MERGE_UPSTREAM] ;
	$UPSTREAM_PARTNER_TO_MERGE =
"$CURRENT_ELEMENT_CONTIGS_SERIES\_$ARRAY_ELEMENT_TO_MERGE_UPSTREAM\_u" ;
	$DOWNSTREAM_PARTNER_TO_MERGE = "$CURRENT_ELEMENT_CONTIGS_SERIES\_A\_d" ;
	$CONTIG_PAIRS_TO_MERGE =
$CONTIG_PAIRS_TO_MERGE.="$UPSTREAM_PARTNER_TO_MERGE\t$DOWNSTREAM_PARTNER_TO_MERGE\n" ;

	my $COUNTER_OTHER_CONTIGS_TO_OUTPUT_UPSTREAM = 0 ;
	while ($COUNTER_OTHER_CONTIGS_TO_OUTPUT_UPSTREAM < $NUMBER_ELEMENTS_SUBASSEMBLY_UPSTREAM)
	{
		my $CURRENT_ELEMENT_SUBASSEMBLY_TO_OUTPUT_UPSTREAM =
$ARRAY_HASH_SUBASSEMBLY_INFO_1[$COUNTER_OTHER_CONTIGS_TO_OUTPUT_UPSTREAM] ;
		my $NAME_UPSTREAM_CONTIG_TO_OUTPUT =
"$CURRENT_ELEMENT_CONTIGS_SERIES\_$CURRENT_ELEMENT_SUBASSEMBLY_TO_OUTPUT_UPSTREAM\_u" ;


		if ($COUNTER_OTHER_CONTIGS_TO_OUTPUT_UPSTREAM < $ARRAY_POSITION_TO_MERGE_UPSTREAM)
	{
			my $UPSTREAM_CONTIG_TO_OUTPUT =
$HASH_PRE_MERGED_CONTIGS{$NAME_UPSTREAM_CONTIG_TO_OUTPUT} ;
			my $LENGTH_TEST_UPSTREAM_CONTIG = length($UPSTREAM_CONTIG_TO_OUTPUT) ;
			if ($LENGTH_TEST_UPSTREAM_CONTIG > 5)	{
				$CONTIGS_TO_OUTPUT_NO_MERGING =
$CONTIGS_TO_OUTPUT_NO_MERGING.="$NAME_UPSTREAM_CONTIG_TO_OUTPUT\n$UPSTREAM_CONTIG_TO_OUTPUT\n" ;
				$CONTIGS_TO_OUTPUT_NO_MERGING_FOR_ACCNOS =
$CONTIGS_TO_OUTPUT_NO_MERGING_FOR_ACCNOS.="$NAME_UPSTREAM_CONTIG_TO_OUTPUT\n" ;

	}

								}
		$COUNTER_OTHER_CONTIGS_TO_OUTPUT_UPSTREAM =
$COUNTER_OTHER_CONTIGS_TO_OUTPUT_UPSTREAM + 1 ;
```

```
                                                                                }

       my $HASH_SUBASSEMBLY_INFO_FOR_CURRENT_ELEMENT_CONTIG_2 =
$HASH_SUBASSEMBLY_DESIGNATORS_DOWNSTREAM{$CURRENT_ELEMENT_CONTIGS_SERIES} ;
       my @ARRAY_HASH_SUBASSEMBLY_INFO_2 =
split(',',$HASH_SUBASSEMBLY_INFO_FOR_CURRENT_ELEMENT_CONTIG_2) ;
       sort(@ARRAY_HASH_SUBASSEMBLY_INFO_2) ;
       $NUMBER_ELEMENTS_SUBASSEMBLY_DOWNSTREAM = scalar(@ARRAY_HASH_SUBASSEMBLY_INFO_2) ;

       my $COUNTER_OTHER_CONTIGS_TO_OUTPUT_DOWNSTREAM = 1 ;
       while ($COUNTER_OTHER_CONTIGS_TO_OUTPUT_DOWNSTREAM <
$NUMBER_ELEMENTS_SUBASSEMBLY_DOWNSTREAM)     {
               my $CURRENT_ELEMENT_SUBASSEMBLY_TO_OUTPUT_DOWNSTREAM =
$ARRAY_HASH_SUBASSEMBLY_INFO_2[$COUNTER_OTHER_CONTIGS_TO_OUTPUT_DOWNSTREAM] ;
               my $NAME_DOWNSTREAM_CONTIG_TO_OUTPUT =
"$CURRENT_ELEMENT_CONTIGS_SERIES\_$CURRENT_ELEMENT_SUBASSEMBLY_TO_OUTPUT_DOWNSTREAM\_d" ;

               my $DOWNSTREAM_CONTIG_TO_OUTPUT =
$HASH_PRE_MERGED_CONTIGS{$NAME_DOWNSTREAM_CONTIG_TO_OUTPUT} ;
               $LENGTH_TEST_DOWNSTREAM_CONTIG = length($DOWNSTREAM_CONTIG_TO_OUTPUT) ;
               if ($LENGTH_TEST_DOWNSTREAM_CONTIG > 5)      {
                       $CONTIGS_TO_OUTPUT_NO_MERGING =
$CONTIGS_TO_OUTPUT_NO_MERGING.="$NAME_DOWNSTREAM_CONTIG_TO_OUTPUT\n$DOWNSTREAM_CONTIG_TO_OUTPUT\n
" ;
                       $CONTIGS_TO_OUTPUT_NO_MERGING_FOR_ACCNOS =
$CONTIGS_TO_OUTPUT_NO_MERGING_FOR_ACCNOS.="$NAME_DOWNSTREAM_CONTIG_TO_OUTPUT\n" ;

       }
               $COUNTER_OTHER_CONTIGS_TO_OUTPUT_DOWNSTREAM =
$COUNTER_OTHER_CONTIGS_TO_OUTPUT_DOWNSTREAM + 1 ;

                                                                                        }

       $COUNTER_CONTIG_SERIES = $COUNTER_CONTIG_SERIES + 1 ;

                               }

print "Merging upstream and downstream pairs" ;

@ARRAY_CONTIG_PAIRS_TO_MERGE = split('\n',$CONTIG_PAIRS_TO_MERGE) ;
$NUMBER_CONTIG_PAIRS_TO_MERGE = scalar(@ARRAY_CONTIG_PAIRS_TO_MERGE) ;
$COUNTER_MERGING_PAIRS = 0 ;
while ($COUNTER_MERGING_PAIRS < $NUMBER_CONTIG_PAIRS_TO_MERGE)      {
       my $CURRENT_PAIR_TO_MERGE = $ARRAY_CONTIG_PAIRS_TO_MERGE[$COUNTER_MERGING_PAIRS] ;
       my @ARRAY_CURRENT_PAIR_TO_MERGE = split('\t',$CURRENT_PAIR_TO_MERGE) ;
       my $NAME_UPSTREAM_TO_MERGE = $ARRAY_CURRENT_PAIR_TO_MERGE[0] ;
       my $NAME_DOWNSTREAM_TO_MERGE = $ARRAY_CURRENT_PAIR_TO_MERGE[1] ;

       $OUTPUT_OVERLAP_NT_IDENTITIES =
$OUTPUT_OVERLAP_NT_IDENTITIES.="$NAME_UPSTREAM_TO_MERGE,$NAME_DOWNSTREAM_TO_MERGE\t" ;


       my $UPSTREAM_SEQUENCE = $HASH_PRE_MERGED_CONTIGS{$NAME_UPSTREAM_TO_MERGE} ;
       my $LENGTH_UPSTREAM_SEQUENCE = length($UPSTREAM_SEQUENCE) ;
#      $OUTPUT_TEST = $OUTPUT_TEST.="upstream\n$UPSTREAM_SEQUENCE\n\n" ;

       my $DOWNSTREAM_SEQUENCE = $HASH_PRE_MERGED_CONTIGS{$NAME_DOWNSTREAM_TO_MERGE} ;
       my $LENGTH_DOWNSTREAM_SEQUENCE = length($DOWNSTREAM_SEQUENCE) ;
#      $OUTPUT_TEST = $OUTPUT_TEST.="downstream\n$DOWNSTREAM_SEQUENCE\n" ;

       print "Finding overlapping sequence to screen with\n" ;
       my $OVERLAP_FOUND = 0 ; my $START_POSITION_DOWNSTREAM_SEQ = 0 ; my $OVERLAP_TEST_LENGTH =
40 ;
       my $OVERLAP_SEQUENCE = "" ;
       my $MAXIMUM_LENGTH_TO_SCREEN = $LENGTH_DOWNSTREAM_SEQUENCE - 50 ;
       while ($OVERLAP_FOUND < 1 and $START_POSITION_DOWNSTREAM_SEQ <
$LENGTH_DOWNSTREAM_SEQUENCE)  {
               my $FIVEPRIME_OF_DOWNSTREAM =
substr($DOWNSTREAM_SEQUENCE,$START_POSITION_DOWNSTREAM_SEQ,$OVERLAP_TEST_LENGTH) ;
```

```
		if ($UPSTREAM_SEQUENCE =~ /$FIVEPRIME_OF_DOWNSTREAM/)	{
			$OVERLAP_FOUND = 2 ;
			$OVERLAP_SEQUENCE = $FIVEPRIME_OF_DOWNSTREAM ;
#			$OUTPUT_TEST = $OUTPUT_TEST.="overlap sequence\n$OVERLAP_SEQUENCE\n" ;

			}
		if ($UPSTREAM_SEQUENCE !~ /$FIVEPRIME_OF_DOWNSTREAM/)	{
			$START_POSITION_DOWNSTREAM_SEQ = $START_POSITION_DOWNSTREAM_SEQ + 1 ;

			}

											}

#	print "Mapping position of overlap sequence in upstream sequence\n" ;
	if ($OVERLAP_FOUND < 1)	{
		$OUTPUT_CONTIG_PAIRS_NOT_MERGED =
$OUTPUT_CONTIG_PAIRS_NOT_MERGED.="$CURRENT_PAIR_TO_MERGE\n" ;
								}

	my $LENGTH_OVERLAP_SEQUENCE = length($OVERLAP_SEQUENCE) ;
	my $START_POSITION_UPSTREAM_SEQ = 0 ;
	my $OVERLAP_POSITION_FOUND_IN_UPSTREAM = 0 ;
	if ($OVERLAP_FOUND > 1 and $LENGTH_OVERLAP_SEQUENCE > 20)	{
		while($OVERLAP_POSITION_FOUND_IN_UPSTREAM < 1)	{
			my $CURRENT_UPSTREAM_TRUNCATION_TO_TEST =
substr($UPSTREAM_SEQUENCE,$START_POSITION_UPSTREAM_SEQ,$OVERLAP_TEST_LENGTH) ;
#			$OUTPUT_TEST = $OUTPUT_TEST.="$CURRENT_UPSTREAM_TRUNCATION_TO_TEST\n" ;
			if ($CURRENT_UPSTREAM_TRUNCATION_TO_TEST =~ /$OVERLAP_SEQUENCE/)	{
				$OVERLAP_POSITION_FOUND_IN_UPSTREAM = 2 ;

								}
			if ($CURRENT_UPSTREAM_TRUNCATION_TO_TEST !~ /$OVERLAP_SEQUENCE/)	{
				$START_POSITION_UPSTREAM_SEQ = $START_POSITION_UPSTREAM_SEQ + 1 ;

								}

	}

			}
#### This section only focuses on the region of the overlap that shows highest identity #####
	if ($OVERLAP_POSITION_FOUND_IN_UPSTREAM > 1)	{
		my $LENGTH_FIVEPRIME_UPSTREAM = $START_POSITION_UPSTREAM_SEQ ;
		my $LENGTH_THREEPRIME_UPSTREAM = $LENGTH_UPSTREAM_SEQUENCE -
$START_POSITION_UPSTREAM_SEQ ;
#		$START_OVERLAP_UPSTREAM = $START_OVERLAP_UPSTREAM + 1 ;
		my $FIVEPRIME_UPSTREAM = substr($UPSTREAM_SEQUENCE,0,$LENGTH_FIVEPRIME_UPSTREAM) ;
		my $THREEPRIME_UPSTREAM =
substr($UPSTREAM_SEQUENCE,$START_POSITION_UPSTREAM_SEQ,$LENGTH_THREEPRIME_UPSTREAM) ;

		my $LENGTH_FIVEPRIME_DOWNSTREAM = $LENGTH_THREEPRIME_UPSTREAM ;
		my $FIVEPRIME_DOWNSTREAM =
substr($DOWNSTREAM_SEQUENCE,$START_POSITION_DOWNSTREAM_SEQ,$LENGTH_FIVEPRIME_DOWNSTREAM) ;

		my $START_POSITION_DOWNSTREAM_THREEPRIME = $START_POSITION_DOWNSTREAM_SEQ +
$LENGTH_FIVEPRIME_DOWNSTREAM ;
		my $LENGTH_THREEPRIME_DOWNSTREAM = $LENGTH_DOWNSTREAM_SEQUENCE -
$START_THREEPRIME_DOWNSTREAM;

		my $THREEPRIME_DOWNSTREAM =
substr($DOWNSTREAM_SEQUENCE,$START_POSITION_DOWNSTREAM_THREEPRIME,$LENGTH_THREEPRIME_DOWNSTREAM)
;

		my $TRUE_OVERLAP = 0 ; my $PERCENT_NT_IDENTITY = 0 ;
		my $LENGTH_THREEPRIME_UPSTREAM = length($THREEPRIME_UPSTREAM) ;
		my $LENGTH_FIVEPRIME_DOWNSTREAM = length($FIVEPRIME_DOWNSTREAM) ;
		if ($LENGTH_THREEPRIME_UPSTREAM == $LENGTH_FIVEPRIME_DOWNSTREAM)	{
			my @ARRAY_THREEPRIME_UPSTREAM = split('',$THREEPRIME_UPSTREAM) ;
			my @ARRAY_FIVEPRIME_DOWNSTREAM = split('',$FIVEPRIME_DOWNSTREAM) ;
			my $COUNTER_OVERLAP_COMPARISON = 0 ;
			my $IDENTICAL_NT = 0 ;
			while ($COUNTER_OVERLAP_COMPARISON < $LENGTH_THREEPRIME_UPSTREAM)	{
```

```
                              my $CURRENT_NT_THREEPRIME_UPSTREAM =
$ARRAY_THREEPRIME_UPSTREAM[$COUNTER_OVERLAP_COMPARISON] ;
                              my $CURRENT_NT_FIVEPRIME_DOWNSTREAM =
$ARRAY_FIVEPRIME_DOWNSTREAM[$COUNTER_OVERLAP_COMPARISON] ;
                              if ($CURRENT_NT_THREEPRIME_UPSTREAM eq
$CURRENT_NT_FIVEPRIME_DOWNSTREAM)     {
                                      $IDENTICAL_NT = $IDENTICAL_NT + 1 ;

                                                                          }
                              $COUNTER_OVERLAP_COMPARISON = $COUNTER_OVERLAP_COMPARISON + 1 ;

                                                      }
                      $PERCENT_NT_IDENTITY = $IDENTICAL_NT/$LENGTH_THREEPRIME_UPSTREAM ;
                      $OUTPUT_OVERLAP_NT_IDENTITIES =
$OUTPUT_OVERLAP_NT_IDENTITIES.="$PERCENT_NT_IDENTITY\t$START_POSITION_DOWNSTREAM_SEQ\t" ;

#                     if ($PERCENT_NT_IDENTITY > 0.9) {
#                             $TRUE_OVERLAP = 2 ;
#                                                                                 }

                                              }

              if ($LENGTH_THREEPRIME_UPSTREAM != $LENGTH_FIVEPRIME_DOWNSTREAM)    {
                      $OUTPUT_OVERLAP_NT_IDENTITIES = $OUTPUT_OVERLAP_NT_IDENTITIES.="NA\tNA\t"
;

                                              }
##############
### This section compares the entire regions that should overlap between downstream and upstream

              my $DOWNSTREAM_FULL_OVERLAP_REGION =
substr($DOWNSTREAM_SEQUENCE,0,$START_POSITION_DOWNSTREAM_THREEPRIME) ;
              my $LENGTH_DOWNSTREAM_FULL_OVERLAP_REGION =
length($DOWNSTREAM_FULL_OVERLAP_REGION) ;
              my $START_POSITION_UPSTREAM_FULL_OVERLAP_REGION = $LENGTH_UPSTREAM_SEQUENCE -
$LENGTH_DOWNSTREAM_FULL_OVERLAP_REGION ;
              my $UPSTREAM_FULL_OVERLAP_REGION =
substr($UPSTREAM_SEQUENCE,$START_POSITION_UPSTREAM_FULL_OVERLAP_REGION,$LENGTH_THREEPRIME_UPSTREA
M) ;

              my @ARRAY_UPSTREAM_FULL_OVERLAP_REGION = split('',$UPSTREAM_FULL_OVERLAP_REGION) ;
              my @ARRAY_DOWNSTREAM_FULL_OVERLAP_REGION =
split('',$DOWNSTREAM_FULL_OVERLAP_REGION) ;
                      my $COUNTER_FULL_OVERLAP_COMPARISON = 0 ;
                      my $IDENTICAL_NT_FULL_OVERLAP_COMPARISON = 0 ;
                      while ($COUNTER_FULL_OVERLAP_COMPARISON <
$LENGTH_DOWNSTREAM_FULL_OVERLAP_REGION)      {
                              my $CURRENT_NT_UPSTREAM_FULL_OVERLAP_REGION =
$ARRAY_UPSTREAM_FULL_OVERLAP_REGION[$COUNTER_FULL_OVERLAP_COMPARISON] ;
                              my $CURRENT_NT_DOWNSTREAM_FULL_OVERLAP_REGION =
$ARRAY_DOWNSTREAM_FULL_OVERLAP_REGION[$COUNTER_FULL_OVERLAP_COMPARISON] ;
                              if ($CURRENT_NT_UPSTREAM_FULL_OVERLAP_REGION eq
$CURRENT_NT_DOWNSTREAM_FULL_OVERLAP_REGION)  {
                                      $IDENTICAL_NT_FULL_OVERLAP_COMPARISON =
$IDENTICAL_NT_FULL_OVERLAP_COMPARISON + 1 ;


              }
                              $COUNTER_FULL_OVERLAP_COMPARISON = $COUNTER_FULL_OVERLAP_COMPARISON
+ 1 ;

                                                                                  }

              $PERCENT_NT_IDENTITY_FULL_OVERLAP_COMPARISON =
$IDENTICAL_NT_FULL_OVERLAP_COMPARISON/$LENGTH_DOWNSTREAM_FULL_OVERLAP_REGION ;
              $OUTPUT_OVERLAP_NT_IDENTITIES =
$OUTPUT_OVERLAP_NT_IDENTITIES.="$PERCENT_NT_IDENTITY_FULL_OVERLAP_COMPARISON\t" ;
              $OUTPUT_OVERLAP_NT_IDENTITIES =
$OUTPUT_OVERLAP_NT_IDENTITIES.="$LENGTH_DOWNSTREAM_FULL_OVERLAP_REGION\t" ;

              if ($PERCENT_NT_IDENTITY_FULL_OVERLAP_COMPARISON > 0.9) {
```

```
                        $TRUE_OVERLAP = 2 ;

                        }
###################
                if ($LENGTH_THREEPRIME_UPSTREAM != $LENGTH_FIVEPRIME_DOWNSTREAM)    {
                        $OUTPUT_TEST = $OUTPUT_TEST.="3 prime upstream\n$THREEPRIME_UPSTREAM\n" ;
                        $OUTPUT_TEST = $OUTPUT_TEST.="5 prime downstream\n$FIVEPRIME_DOWNSTREAM\n"
;
#                       $OUTPUT_OVERLAP_NT_IDENTITIES = $OUTPUT_OVERLAP_NT_IDENTITIES.="NA\tNA\t"
;

                }

                if ($TRUE_OVERLAP > 1)        {
                        my @ARRAY_FOR_NAME_DESIGNATORS = split('\_',$NAME_UPSTREAM_TO_MERGE) ;
                        my $CONTIG_SERIES_DESIGNATOR_1 = $ARRAY_FOR_NAME_DESIGNATORS[0] ;
                        my $CONTIG_SERIES_DESIGNATOR_2 = $ARRAY_FOR_NAME_DESIGNATORS[1] ;
                        my $CONTIG_NUMBER_DESIGNATOR = $ARRAY_FOR_NAME_DESIGNATORS[2] ;
                        my $NAME_FOR_MERGED_CONTIG =
"$CONTIG_SERIES_DESIGNATOR_1\_$CONTIG_SERIES_DESIGNATOR_2\_" ;
                        $NAME_FOR_MERGED_CONTIG =
$NAME_FOR_MERGED_CONTIG.="$CONTIG_NUMBER_DESIGNATOR\_m" ;
                        $OUTPUT = $OUTPUT.="$NAME_FOR_MERGED_CONTIG\n" ;
                        if ($LENGTH_UPSTREAM_SEQUENCE >= $LENGTH_DOWNSTREAM_SEQUENCE)       {
                                $OUTPUT = $OUTPUT.="$FIVEPRIME_UPSTREAM" ;
                                $OUTPUT = $OUTPUT.="$THREEPRIME_UPSTREAM" ;
                                $OUTPUT = $OUTPUT.="$THREEPRIME_DOWNSTREAM\n" ;

                                        }
                        if ($LENGTH_UPSTREAM_SEQUENCE < $LENGTH_DOWNSTREAM_SEQUENCE)        {
                                $OUTPUT = $OUTPUT.="$FIVEPRIME_UPSTREAM" ;
                                $OUTPUT = $OUTPUT.="$FIVEPRIME_DOWNSTREAM" ;
                                $OUTPUT = $OUTPUT.="$THREEPRIME_DOWNSTREAM\n" ;

                                        }
                                                        }

                if ($TRUE_OVERLAP < 1)        {
                        $OUTPUT_CONTIG_PAIRS_NOT_MERGED =
$OUTPUT_CONTIG_PAIRS_NOT_MERGED.="$CURRENT_PAIR_TO_MERGE\n" ;
                        $CONTIGS_TO_OUTPUT_NO_MERGING =
$CONTIGS_TO_OUTPUT_NO_MERGING.="$NAME_UPSTREAM_TO_MERGE\n$UPSTREAM_SEQUENCE\n" ;
                        $CONTIGS_TO_OUTPUT_NO_MERGING =
$CONTIGS_TO_OUTPUT_NO_MERGING.="$NAME_DOWNSTREAM_TO_MERGE\n$DOWNSTREAM_SEQUENCE\n" ;
                                                        }

        }
        if ($OVERLAP_POSITION_FOUND_IN_UPSTREAM < 1)        {
                        $OUTPUT_CONTIG_PAIRS_NOT_MERGED =
$OUTPUT_CONTIG_PAIRS_NOT_MERGED.="$CURRENT_PAIR_TO_MERGE\n" ;
                        $CONTIGS_TO_OUTPUT_NO_MERGING =
$CONTIGS_TO_OUTPUT_NO_MERGING.="$NAME_UPSTREAM_TO_MERGE\n$UPSTREAM_SEQUENCE\n" ;
                        $CONTIGS_TO_OUTPUT_NO_MERGING =
$CONTIGS_TO_OUTPUT_NO_MERGING.="$NAME_DOWNSTREAM_TO_MERGE\n$DOWNSTREAM_SEQUENCE\n" ;
                        $OUTPUT_OVERLAP_NT_IDENTITIES = $OUTPUT_OVERLAP_NT_IDENTITIES.="NA\tNA\t"
;

        }
        $OUTPUT_OVERLAP_NT_IDENTITIES = $OUTPUT_OVERLAP_NT_IDENTITIES.="\n" ;
############################ END OF COPIED AND PASTED CONTENT



        $COUNTER_MERGING_PAIRS = $COUNTER_MERGING_PAIRS + 1 ;

                        }

open (OUTPUT, ">OUTPUT contigs_merged $NAME_OF_FILE_FOR_OUTPUT") or die "Can't open $file for
reading" ;
print OUTPUT "$OUTPUT\n" ;
close (OUTPUT);
```

```
open (OUTPUT, ">OUTPUT contigs_NOT_merged $NAME_OF_FILE_FOR_OUTPUT") or die "Can't open $file for
reading" ;
print OUTPUT "$CONTIGS_TO_OUTPUT_NO_MERGING\n" ;
close (OUTPUT);

open (OUTPUT_IDENTITIES, ">OUTPUT_identity in overlap survey for merged contigs
$NAME_OF_FILE_FOR_OUTPUT.txt") or die "Can't open $file for reading" ;

print OUTPUT_IDENTITIES "$OUTPUT_OVERLAP_NT_IDENTITIES\n\n" ;
print OUTPUT_IDENTITIES "$OUTPUT_CONTIG_PAIRS_NOT_MERGED\n" ;
close (OUTPUT_IDENTITIES);

#open (OUTPUT_TEST, ">OUTPUT_TEST_merging script.txt") or die "Can't open $file for reading" ;

#print OUTPUT_TEST "$CONTIG_PAIRS_TO_MERGE" ;
#print OUTPUT_TEST "$OUTPUT_TEST" ;
#close (OUTPUT_TEST);

#open (OUTPUT_TEST_2, ">OUTPUT_TEST2_merging script.txt") or die "Can't open $file for reading" ;

#print OUTPUT_TEST_2 "$CONTIGS_TO_OUTPUT_NO_MERGING_FOR_ACCNOS" ;
#close (OUTPUT_TEST_2);
```
