# Supplementary material for: Enrichment of Candidate Formate and Acetaldehyde-Producing Ruminal Bacteria in Response to Corn-Based Diets
Source: Microorganisms. 2026 Sep 22;14(10):2134. doi: 10.3390/microorganisms14102134 (PMC13707844; doi:10.3390/microorganisms14102134)
Supplement: Supplementary file 1 [file microorganisms-14-02134-s001.zip › Anema et al_Supplementary File S4_aa and ATP pathways.pdf]

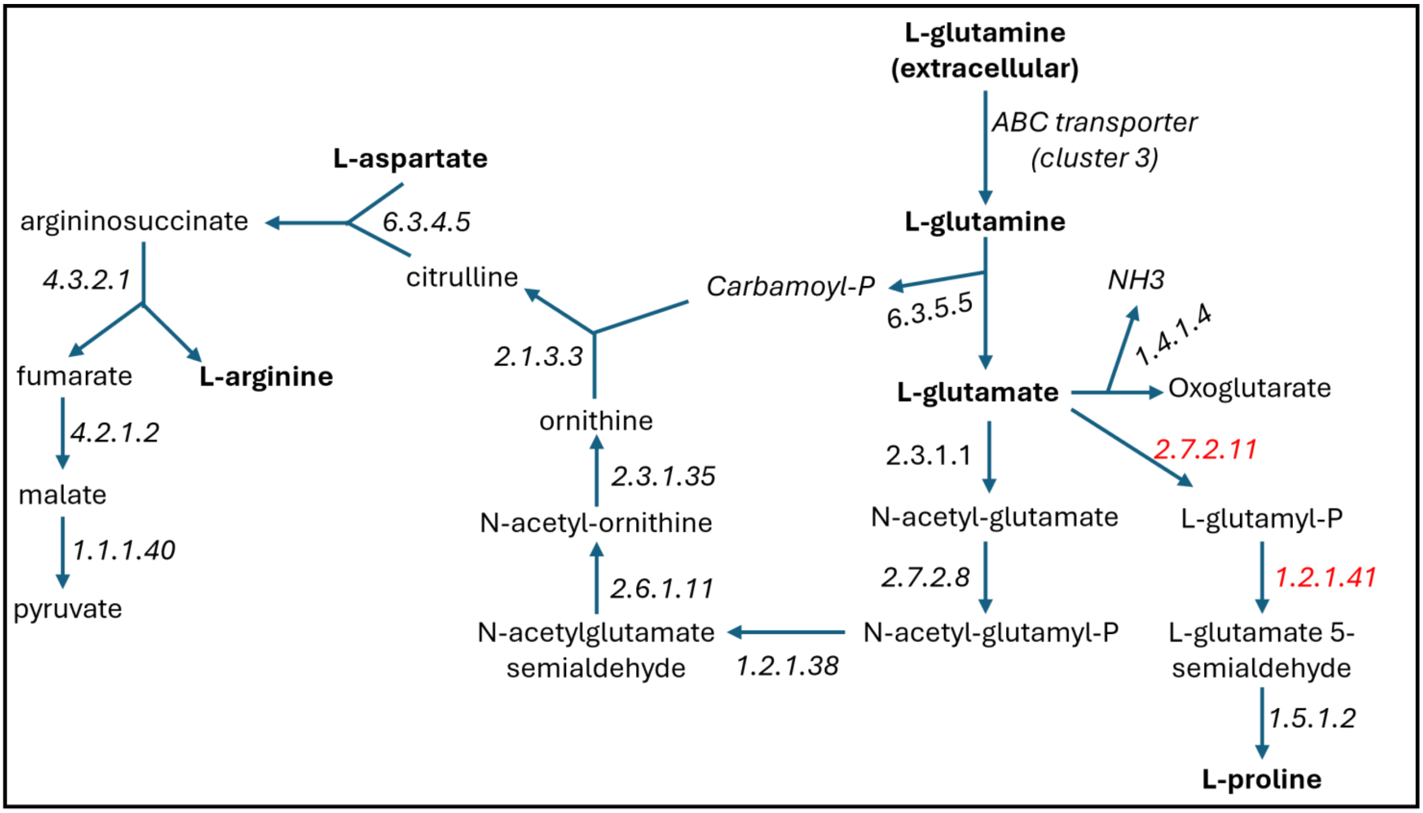

Supplemental Figure S1. Proposed metabolic pathways from the OTU Oa-00002 contig set for synthesis of L-glutamate, L-arginine and L-proline using L-glutamine and L-aspartate as precursors. Codes shown represent the Enzyme Commission (EC) numbers of their corresponding enzymes. EC numbers shown in red font indicate that coding sequences for their corresponding enzymes were not found in the Oa-00002 contig set.

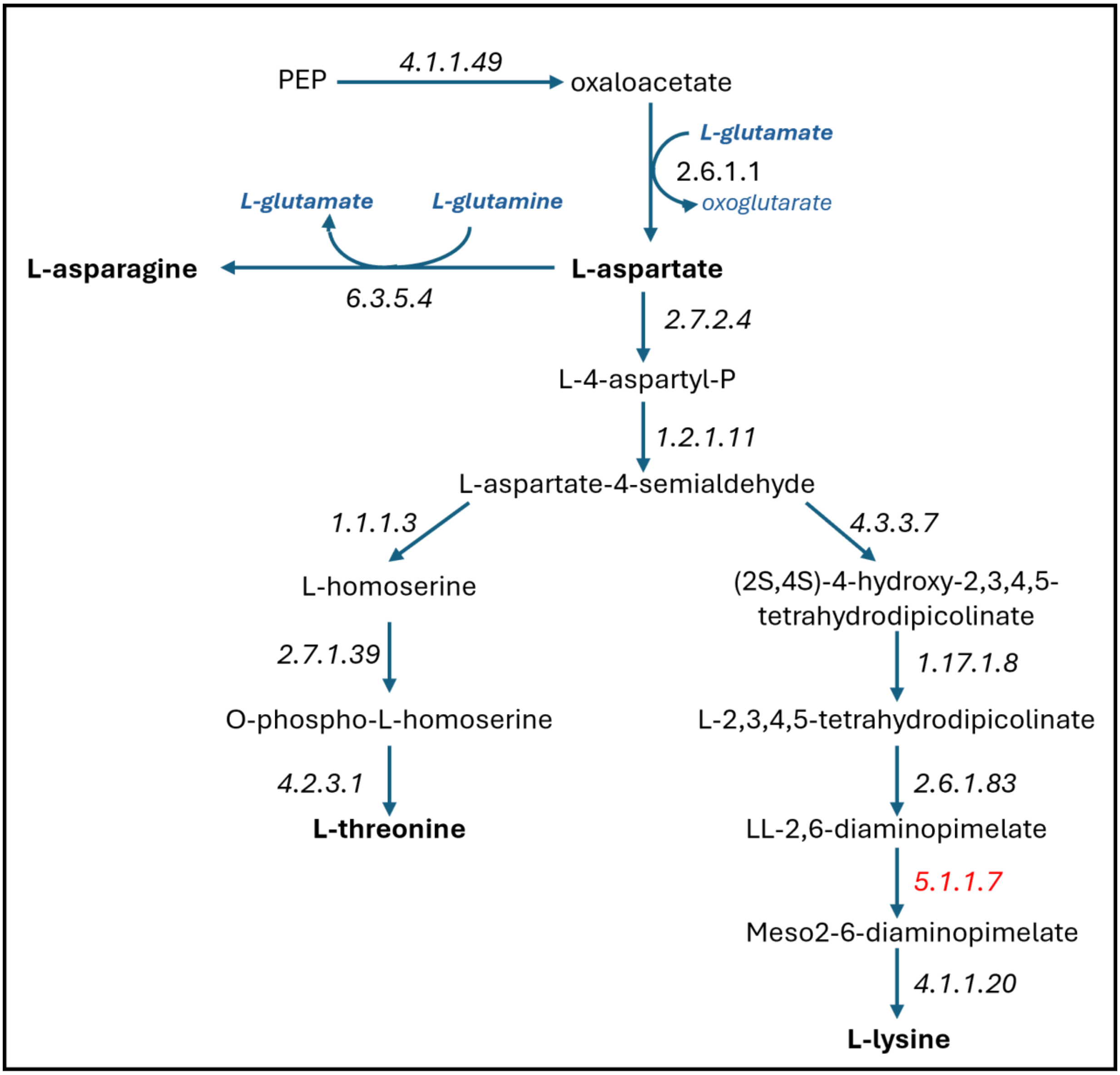

Supplemental Figure S2. Proposed metabolic pathways from the OTU Oa-00002 contig set for synthesis of L-aspartate, L-asparagine, L-threonine, and L-lysine using phosphoenol pyruvate (PEP, glycolysis intermediate) as precursor, as well as L-glutamate and L-glutamine as the amino group donors. Codes shown represent the Enzyme Commission (EC) numbers of their corresponding enzymes. The EC number in red font indicates that coding sequences for this enzyme were not found in the Oa-00002 contig set.

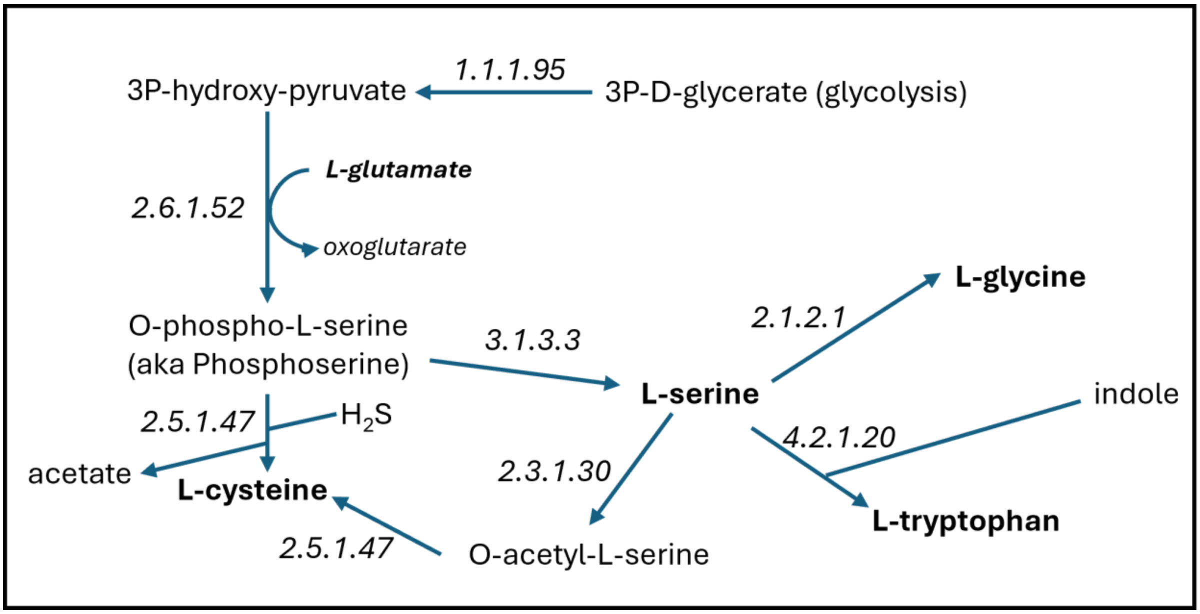

Supplemental Figure S3. Proposed metabolic pathways from the OTU Oa-00002 contig set for synthesis of L-cysteine, L-serine, L-glycine, and L-tryptophan using 3-phospho-D-glycerate (glycolysis intermediate) as precursor and L-glutamate as the amino group donor. Codes shown represent the Enzyme Commission (EC) numbers of their corresponding enzymes.

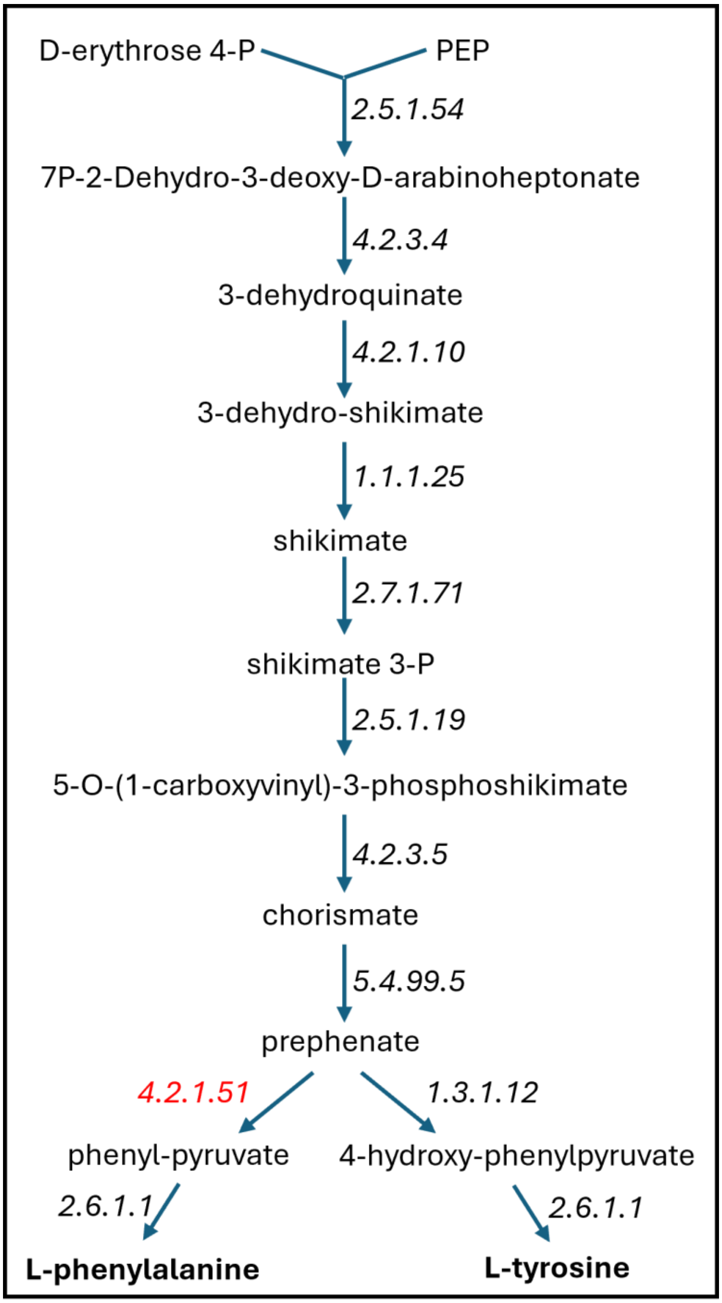

Supplemental Figure S4. Proposed metabolic pathway from the OTU Oa-00002 contig set for synthesis of L-phenylalanine and L-tyrosine by the shikimate pathway. Codes shown represent the Enzyme Commission (EC) numbers of their corresponding enzymes. The EC number in red font indicates that coding sequences for this enzyme were not found in the Oa-00002 contig set.

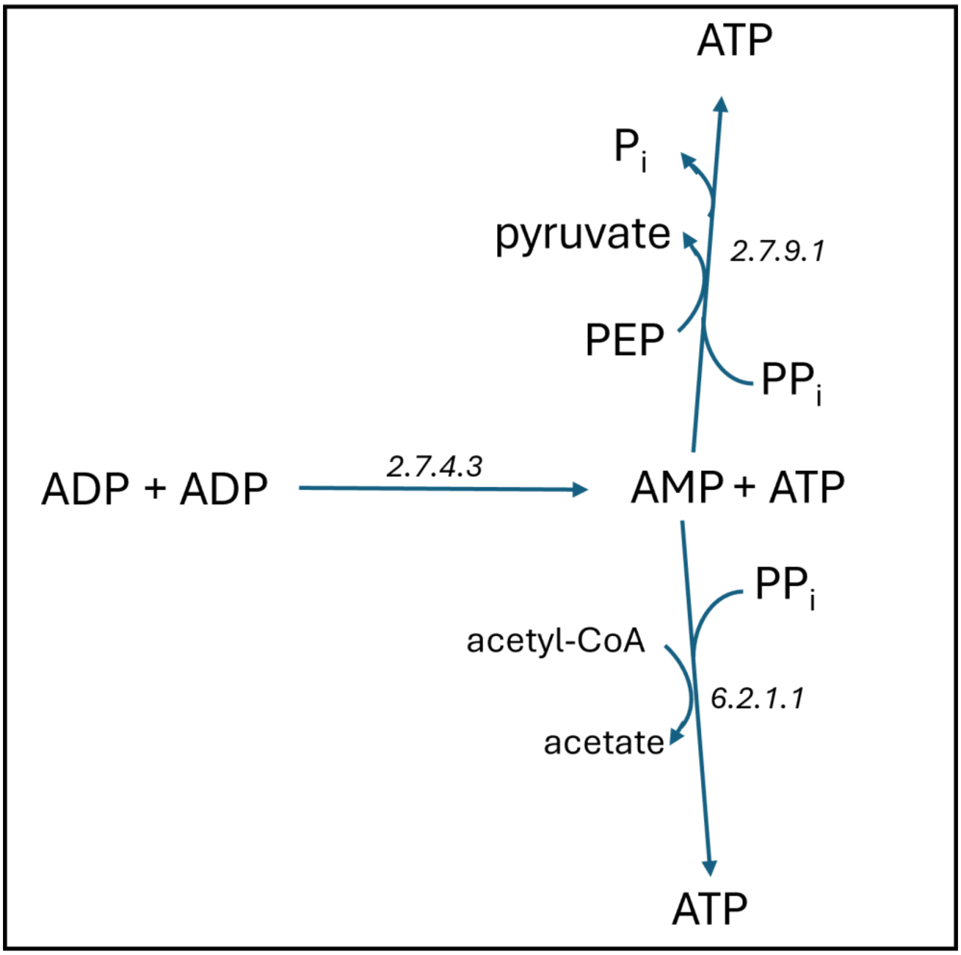

Supplemental Figure S5. Proposed reactions for production of ATP from AMP by substrate-level phosphorylation, as predicted by the Lachnospiraceae contig set. Each enzymes is represented by its respective Enzyme Commission (EC) number.
